# A comprehensive evaluation of microbial differential abundance analysis methods: current status and potential solutions

**DOI:** 10.1186/s40168-022-01320-0

**Published:** 2022-08-19

**Authors:** Lu Yang, Jun Chen

**Affiliations:** 1 Division of Computational Biology, Department of Quantitative Health Sciences, Mayo Clinic, Rochester, MN 55905 USA; 2 Center for Individualized Medicine, Mayo Clinic, Rochester, MN 55905 USA

**Keywords:** Microbiome, Metagenomics, Statistical methods, Differential abundance analysis, False discovery rate, Compositional effects, Zero inflation, Benchmarking

## Abstract

**Background:**

Differential abundance analysis (DAA) is one central statistical task in microbiome data analysis. A robust and powerful DAA tool can help identify highly confident microbial candidates for further biological validation. Numerous DAA tools have been proposed in the past decade addressing the special characteristics of microbiome data such as zero inflation and compositional effects. Disturbingly, different DAA tools could sometimes produce quite discordant results, opening to the possibility of cherry-picking the tool in favor of one’s own hypothesis. To recommend the best DAA tool or practice to the field, a comprehensive evaluation, which covers as many biologically relevant scenarios as possible, is critically needed.

**Results:**

We performed by far the most comprehensive evaluation of existing DAA tools using real data-based simulations. We found that DAA methods explicitly addressing compositional effects such as ANCOM-BC, Aldex2, metagenomeSeq (fitFeatureModel), and DACOMP did have improved performance in false-positive control. But they are still not optimal: type 1 error inflation or low statistical power has been observed in many settings. The recent LDM method generally had the best power, but its false-positive control in the presence of strong compositional effects was not satisfactory. Overall, none of the evaluated methods is simultaneously robust, powerful, and flexible, which makes the selection of the best DAA tool difficult. To meet the analysis needs, we designed an optimized procedure, ZicoSeq, drawing on the strength of the existing DAA methods. We show that ZicoSeq generally controlled for false positives across settings, and the power was among the highest. Application of DAA methods to a large collection of real datasets revealed a similar pattern observed in simulation studies.

**Conclusions:**

Based on the benchmarking study, we conclude that none of the existing DAA methods evaluated can be applied blindly to any real microbiome dataset. The applicability of an existing DAA method depends on specific settings, which are usually unknown a priori. To circumvent the difficulty of selecting the best DAA tool in practice, we design ZicoSeq, which addresses the major challenges in DAA and remedies the drawbacks of existing DAA methods. ZicoSeq can be applied to microbiome datasets from diverse settings and is a useful DAA tool for robust microbiome biomarker discovery.

Video Abstract

**Supplementary Information:**

The online version contains supplementary material available at 10.1186/s40168-022-01320-0.

## Background

The human microbiome has received tremendous attention in the past decade due to its potential important role in health and disease [1]. A variety of conditions and diseases such as obesity, inflammatory bowel disease, and colorectal cancer have been shown to be associated with changes in the human gut microbiome [2–4]. Gut microbiome has also been demonstrated to be predictive of the cancer treatment outcome, spurring enthusiasm among cancer researchers in pursuit of a new cancer treatment paradigm [5, 6]. With the aid of high-throughput genomic sequencing technologies, such as 16S rRNA gene-targeted amplicon sequencing and shotgun metagenomic sequencing, the microbiome composition can be easily profiled and analyzed [7]. After processing the sequence reads using a bioinformatic pipeline of choice such as DADA2 [8] for 16S-targeted sequencing and MetaPhlAn2 [9] for shotgun metagenomic data, an abundance table, which records the frequencies of the detected microbial taxa, is generated. Downstream statistical analyses are then performed based on the taxa abundance table, together with the metadata capturing the sample-level characteristics. One central statistical task is differential abundance analysis (DAA), which aims to identify the microbial taxa whose abundance covaries with a variable of interest. The identified microbial taxa could offer biological insights into disease mechanisms and potentially be explored as biomarkers for disease prevention, diagnosis, and treatment [10]. A robust and powerful DAA tool is thus critically needed to yield reliable microbiome biomarkers, increase the reproducibility across microbiome studies, and ultimately reduce the development cost.

Due to the complex data characteristics of microbiome sequencing data, differential abundance analysis of microbiome data faces many statistical challenges [11, 12]. Firstly, the microbiome abundance data are highly variable, and the abundance of a specific taxon could range over several orders of magnitude. Such large variability deteriorates statistical power, calling for powerful methods which could appropriately model the variance of the data. Secondly, the microbiome abundance data are zero inflated [12–14]. In a typical microbiome dataset, more than 70% of the values are zeros. Zeros could be due to either physical absence (structural zeros) or insufficient sampling effort (sampling zeros) [13, 14]. The different natures of zeros require careful treatment of the zeros in order to reach robust statistical inference [13, 14]. For those low-abundance taxa, when their abundance falls below the detection limit, they will appear absent in the data. Therefore, the presence/absence of a low-abundance taxon [13, 14] depends highly on the total read count (sequencing depth). This has significant implications for differential abundance analysis. When the sequencing depth is correlated with the variable of interest, those low-abundance taxa may appear differentially abundant even after the read counts have been normalized [15]. Thirdly, microbiome data are compositional [15–17]. All we know are the relative abundances since the total read count does not reflect the microbial load at the sampling site [17, 18]. Increase or decrease in the (absolute) abundance of one taxon at the sampling site will lead to apparent changes in the relative abundances of other taxa in the sample. Such compositional effect makes identification of the “driver” taxa particularly challenging due to missing information on the total microbial load. Although all sequencing data are compositional in nature [19], the existence of several highly abundant taxa amid a large number of low-abundance taxa makes the compositional effect more pronounced for microbiome data.

Without any assumption, DAA for compositional data is not well defined. Consider a hypothetical community with four species, whose baseline absolute abundances at the sampling site are 7, 2, 6, and 10 million cells per unit volume. After experimental treatment, the abundances become 2, 2, 6, and 10 million cells per unit volume, where only the first species is differential. The compositions before and after treatment are (28%, 8%, 24%, and 40%) and (10%, 10%, 30%, and 50%), respectively. Now assume that the absolute abundances for the four species before treatment are known (7, 2, 6, 10), the observed composition after treatment can be explained equally well by (2, 2, 6, 10), (7, 7, 21, 35), or (20, 20, 60, 100) million cells per unit volume after treatment. Therefore, based on the compositional data alone, it is equally possible that there are one, three, or four differential taxa. However, if we assume the signal is sparse (i.e., the number of differential taxa is small), we may conclude that the first species being differential is the most likely scenario. The sparsity assumption has been implicitly assumed for those methods addressing the compositional effects.

Over the past decade, quite a few DAA methods have been developed. These methods mainly differ in their way to address zero inflation and compositional effects. To address zero inflation, both over-dispersed count models and zero-inflated mixture models/hurdle models have been proposed. In over-dispersed count model, the counts are modeled by a parametric model with an overdispersion parameter, which controls the variability of the data as well as the level of sparsity. Examples include the negative binomial model (edgeR [20] and DESeq2 [21]), beta-binomial model (corncorb [22]), and quasi-Poisson model [23]. These count models implicitly assume that all zeros are sampling zeros due to insufficient sequencing depth. While this assumption is reasonable for the vast majority of low-abundance taxa [14], zeros for those abundant taxa may not be solely explained by under-sampling [24]. In contrast, mixture models, which include a mixture component at zero, are more flexible; it assumes both sampling and structural zeros exist in the data. The extra parameter for the structural zero component significantly increases the modeling capability for zero-inflated counts. However, the drawbacks of mixture models are the increased computational burden and potential loss of power due to overfitting when there is no truly zero inflation, i.e., the zero component is not necessary. Overfitting could also lead to computational instability since there could be multiple optima in the parameter space. Examples of zero-inflated mixture models include zero-inflated log-normal/normal mixture model (metagenomeSeq [25] and RAIDA [26]), zero-inflated beta-binomial model (ZIBB [27]), and zero-inflated negative binomial model (Omnibus test [24]). RioNorm2 [28] uses a data-driven approach to choose between zero-inflated Poisson model and zero-inflated negative binomial model. As an alternative to mixture models, hurdle models [29, 30] have also been proposed to perform DAA. Hurdle models lump the sampling and structural zeros together in the zero component and do not distinguish between these two types of zeros. Additionally, for methods working on proportion data, Bayesian methods have been used to impute the zeros, accounting for sampling variability and sequencing depth variation. For example, ALDEx2 [31] infers the underlying proportions by assuming an uninformative prior Dirichlet distribution on the proportions and a multinomial sampling process for the observed counts. eBay [32] uses an Empirical Bayes approach with an informative prior, which is estimated based on the data, to improve the estimation efficiency. On the other hand, MaAsLin2 [33] and ANCOM-BC [34] use the pseudo-count approach to impute the zeros. When a common pseudo-count is added to all counts, the process is equivalent to a Bayesian approach assuming a non-informative prior. Finally, for methods without the involvement of log transformation, zeros may also be left untreated as in LDM [35] and DACOMP [36].

Compositional effects are another major challenge facing DAA [15–19]. The severity of compositional effects depends on the diversity of the microbial community, the percentage of differential taxa, and their abundances, effect sizes, and directions of change. Different strategies have been used to address compositional effects. These strategies can be roughly divided into four categories. The first category is based on robust normalization (Table S1), where a normalizing factor or size factor is calculated to capture the sequencing effort for the non-differential part as much as possible, assuming sparsity signals [37]. The normalizing factor can then be included as an offset in count-based models or be used as a divider to produce normalized abundance data. Compared to the total sum scaling (TSS) normalization, robust normalization is less susceptible to compositional effects when a moderate number of taxa are differential [37]. Robust normalization has been used in edgeR [20], DESeq2 [21], metagenomeSeq [25], ALDEx2 [31], and Omnibus test [24], where the Trimmed mean of *M*-values (TMM), relative log expression (RLE), cumulative sum scaling (CSS), centered log-ratio transformation (CLR), and geometric mean of pairwise ratios (GMPR) [37] are used, respectively. The Wrench [38] normalization corrects the compositional bias by an empirical Bayes approach, which has been recommended in metagenomeSeq [39]. The second category uses the reference taxa approach, which aims to find one taxon or a set of taxa that are relatively invariant with respect to the condition of interest. The abundance ratios to the reference taxon/taxa are then used to perform DAA. RioNorm2 [28] relies on a network-based normalization to find the relatively invariant taxa. DACOMP [36] selects a set of reference taxa that are least likely to be differential before DAA, while RAIDA [26] finds one reference taxon that makes the least discoveries in DAA. The differential ranking method [17] utilizes a similar reference taxa idea. The third category is based on analyzing the pattern of pairwise log ratios as implemented in ANCOM [18]. This strategy relies on the fact that the log ratios to other taxa for those non-differential taxa are mostly non-differential, while the log ratios for those differential taxa are all differential, assuming distinct effect sizes. Therefore, by analyzing the pattern of pairwise log ratios, the differential taxa can be recovered with high confidence. DACOMP [36] also uses this approach to select the reference taxa. The last category exploits the novel bias-correction idea. ANCOM-BC [34] uses this approach to estimate an (unknown) sample-specific offset term to correct the bias caused by an unequal sampling effort due to compositional effects.

A wild choice of DAA methods dazzles the end users. Numerous questions arise regarding the best DAA method for one’s specific dataset. To date, no consistent recommendations have been clearly provided to end users [15, 40, 41] and a comprehensive benchmarking study of existing methods is critically needed. In our opinion, an ideal DAA method should possess the following properties:

1. Scalable: It should be able to scale up to a large number of taxa and samples, given the increased availability of large datasets [42, 43].
2. Flexible: It should be able to adjust covariates and accommodate different study designs. Confounders are common for microbiome studies [44–48], and adjusting confounder is necessary to reach a valid conclusion.
3. Robust: It should control for false positives under all relevant scenarios. The actual type 1 error rate should be close to the nominal level. This is the key to the reproducibility of microbiome studies.
4. Powerful: The power to identify true positives should not be sacrificed to preserve the type 1 error rate.

Although several evaluation studies were published years ago [41, 49], the fast development in this field calls for a re-evaluation of the old and new DAA methods in order to offer a practical guidance to the field. Therefore, the aim of the study is to perform a comprehensive evaluation of existing DAA methods, identify their strength and weakness, recommend the optimal procedure to the field if any, and develop an alternative if no DAA methods can satisfy all the aforementioned properties.

The contribution of the study is threefold. First, we designed a real data-based semiparametric simulation framework, which facilitates a more realistic evaluation of the performance of DAA methods; second, we conducted by far the most comprehensive benchmarking study and dissected the performance of existing methods; and finally, realizing no methods evaluated possess the optimal performance, we developed an optimized procedure, ZicoSeq, which combines the strength of DACOMP (good false-positive control) and LDM (high power). We implemented our semiparametric simulation framework and ZicoSeq in our Comprehensive R Archive Network (CRAN) *GUniFrac* package.

## Methods

### A semiparametric simulation framework

Traditional simulations are usually based on parametric models such as Dirichlet-multinomial model [50, 51] and logistic normal multinomial model [41]. The sample space is thus determined by a small set of parameters. Due to the complexity of the microbiome data, existing parametric models may fail to capture the full complexity of the data. To correct the limitation of parametric models, our semiparametric simulation framework draws random samples from a large reference dataset (nonparametric part) and uses these reference samples as templates to generate new samples (parametric part). Specifically, for each drawn reference sample, we infer the underlying composition based on a Bayesian model and then add covariate/confounder effects to the composition vector, based on which a new sequencing sample is generated. Therefore, our method circumvents the difficulty in modeling the intersubject variation of the microbiome composition.

The basic steps of the semiparametric simulation framework are depicted in Fig. S1. Specially, we use the following steps to simulate the data:

1. Build a reference dataset. The reference dataset is a collection of microbiome sequencing samples from a study population at a specific sampling site. It should be large enough to capture the main compositional variation in the population of interest. Microbiome datasets from those large-scale population-level studies such as Human Microbiome Project (HMP) [42] and American Gut Project (AGP) [43] are all good choices. The reference datasets used in the simulation are the human stool and vaginal microbiome datasets from HMP with basic filtering to remove extremely rare taxa (prevalence < 10% or max proportion < 0.002), resulting in 295 samples and 2094 taxa, and 381 samples and 781 taxa for stool and vaginal datasets, respectively. The human stool and vaginal microbiome are chosen to represent a high- and low-diversity microbial community, respectively.
2. Obtain posterior samples of the underlying composition based on an empirical Bayes model.
  - Assume an informative Dirichlet prior for the underlying composition. Estimate the Dirichlet hyperparameters (γ_*j*_) based on the observed counts (*C*_*ij*_ ,1 ≤ *i* ≤ *n*, 1 ≤ *j* ≤ *m*) using the maximum likelihood estimation (R package “dirmult”). The posterior distribution of the underlying composition for sample *i* is then a Dirichlet distribution with parameter ${\upgamma}_{ij}^{\prime }={C}_{ij}+{\upgamma}_j\ \left(1\le j\le m\right)$.
  - Obtain a posterior sample of the underlying composition for each reference sample (*P*_*ij*_, 1 ≤ *i* ≤ *n*, 1 ≤ *j* ≤ *m*) based on the posterior Dirichlet distribution.
3. Generate the absolute abundance (${C}_{ij}^{\prime}\Big)$ by multiplying a factor *S*_*i*_ representing the microbial load at the ecological site, i.e., ${C}_{ij}^{\prime }={P}_{ij}{S}_i,$where log(*S*_*i*_)~*N*(0, 1) without loss of generality.
4. Generate the confounder *Z*_*i*_~*N*(0, 1) and the covariate of interest *X*_*i*_= $\sqrt{\frac{R^2}{1-{R}^2}}{Z}_i+N\ \left(0,1\right),$where *R* is the desired correlation between the confounder and the covariate of interest. Binary *X*_*i*_ can be generated by dichotomizing *X*_*i*_ using some cutoff value to achieve the specified group sizes.
5. Add covariate (*X*_*i*_) and confounder (*Z*_*i*_) effect to the absolute abundance by ${C}_{ij}^{\prime \prime }={C}_{ij}^{\prime}\exp \left({a}_j{X}_i+{b}_j{Z}_i+{\epsilon}_{ij}\right),$where *a*_*j*_ and *b*_*j*_ are coefficients controlling the effect size and ${\epsilon}_{ij}\sim N\left(0,{\sigma}_{\epsilon}^2\right)$ is the random error. Non-differential taxa are simulated by setting the corresponding coefficients to 0.
6. Calculate the new composition ${P}_{ij}^{\prime}\left(1\le i\le n,1\le j\le m\right)$based on ${C}_{ij}^{\prime \prime }.$Generate the sequencing depth *D*_*i*_ (1 ≤ *i* ≤ *n*) based on a negative binomial distribution. For *b*th simulated dataset (1 ≤ *b* ≤ *B*), generate the read counts ${C}_{ij}^b\ \left(1\le j\le m\right)$for sample *i* based on a multinomial distribution with parameters $\left({D}_i,{P}_{ij}^{\prime}\right)$.

When assessing the model fit of the semiparametric approach, we used cross-validation, where half of the real data were used as the training set to generate simulated data and the other half of the real data served as the test set, upon which the simulated data were compared to.

### Evaluation of differential abundance analysis methods based on simulations

#### Simulation settings

To comprehensively evaluate the performance of DAA methods, we simulate various settings covering a wide range of signal structures (Table 1). We focus the evaluation on two-group comparison with equal group sizes (binary X_i_). We study the performance for both low- and high-diversity data and investigate both the balanced and unbalanced differential settings, where the differential taxa have a random (“balanced”) or the same direction of change (“unbalanced”). The unbalanced setting creates strong compositional effects and is statistically more challenging than the balanced setting. Under the balanced setting, we explore different signal structures (signal density and differential mode). We study three levels of signal densities (i.e., the percentage of differential taxa): 5%, 10%, and 20%, representing “low,” “medium,” and “high” densities, and two differential modes (“abundant” and “rare”), depending on whether the signals come from abundant or rare taxa. In the “abundant” and “rare” differential mode, the differential taxa are drawn from the upper and the lower quartile of the abundance distribution, respectively. These two differential modes allow us to further dissect the performance of the DAA methods. When the differential taxa are abundant, the major challenge is proper false-positive rate control since these abundant taxa could create stronger compositional effects. When the differential taxa are less abundant or rare, the major challenge is low statistical power since rare taxa tend to have more sampling variability. Under the unbalanced setting, we only study the “abundant” differential mode: this is used to create ultra-strong compositional effects to test the limits of the evaluated methods in addressing compositional effects. Under each setting/signal structure, we study the effect of sample size (*n* = 25, 50, and 100 per group) and the taxa number (*m* = 50 and 500, roughly representing family- and species-level data). When confounders are included, we simulate one continuous confounder, and the correlation between covariate and confounder is set to 0.6. In these settings, the sequencing depths are generated from the same negative binomial distribution with mean depth 10,000, and the sequencing depth is similar between the two groups. To study the effect of the sequencing depth confounding, we also let the mean sequencing depth differ by four- or ninefold between the groups.

**Table 1:** Configurations of the simulation settings used in the evaluation of differential abundant analysis methods

Setting				Sample size (***n***)			Taxa number (***m***)		Depth confounding			Signal density			Differential mode	
				50	100	200	50	500	No	Yes (-fold)		Low	Medium	High	Abundant	Rare
										4	9	5%	10%	20%		
Binary	Global null		1	✓		✓	✓	✓	✓							
	Balanced	Basic setting	2		✓			✓	✓			✓	✓	✓	✓	✓
		Small *n*	3	✓		✓		✓	✓			✓	✓	✓	✓	✓
		Small *m*	4		✓		✓		✓			✓	✓	✓	✓	✓
		Depth confounding	5		✓			✓		✓	✓	✓	✓	✓	✓	✓
	Unbalanced	Basic setting	6		✓			✓	✓			✓	✓	✓	✓	
		Small *n*	7	✓				✓	✓			✓	✓	✓	✓	
		Small *m*	8		✓		✓	✓	✓			✓	✓	✓	✓	
Binary + confounder	Balanced	Basic setting	9		✓			✓	✓			✓	✓	✓	✓	✓
	Unbalanced	Basic setting	10		✓			✓	✓			✓	✓	✓	✓	

#### Differential abundance analysis methods evaluated

We evaluate the widely used and recently developed DAA methods including Aldex2 [31], eBay [32], DACOMP [36], ANCOM-BC [34], metagenomeSeq [25], generalized linear model with quasi-Poisson family [23], Wilcoxon rank-sum test, DESeq2 [21], edgeR [20], LDM [35], Omnibus test [24], MaAsLin2 [33], RAIDA [26], and corncob [22]. The summary of the methods is given in Table 2 and Table S2. For Aldex2, eBay, and DACOMP, we choose the Wilcoxon rank-sum test in these packages and label them as “Aldex2(Wilcox)”, “eBay(Wilcox),” and “DACOMP,” respectively. For datasets with confounders, we use the “glm” test in Aldex2 and label it as “Aldex2(glm).” For ANCOM-BC, we set “conserve =TRUE,” since it is recommended if the sample size is small, and/or the number of differentially abundant taxa is believed to be large as indicated in the software tutorial. For metagenomeSeq, we use “fitFeatureModel” with the recommended Wrench normalization method as described in the package tutorial [39]. We label the resulting procedure as “MSeq(Wrench).” We also include the traditional generalized linear model (glm) with a quasi-Poisson family and a log link function. To account for library size variation, we calculate the geometric mean of pairwise ratios (GMPR) size factor [37] and use the log(GMPR size factor) as the offset. Wald test is used for significance testing. We label this procedure as “GMPR + glm.” For Wilcoxon rank-sum test, we compare three different normalization strategies including total sum scaling (TSS), rarefaction, and GMPR normalization. They are labeled as “TSS + Wilcox,” “Rarefy + Wilcox,” and “GMPR + Wilcox,” respectively. edgeR and DESeq2, which have been widely used for microbiome data [40], are also compared. Instead of using their native size factor, which is not appropriate for microbiome data due to zero inflation, we use the GMPR size factor instead, and the resulting procedures are denoted as “GMPR + DESeq2” and “GMPR + edgeR.”

**Table 2:** Differential abundant analysis methods evaluated in this study

Method	Addressing compositional effects	Handling zeros	Model	Covariate/confounder adjustment
GMPR + Wilcox	GMPR	None	Wilcoxon rank-sum test	✗
TSS + Wilcox	Total sum scaling (TSS)			✗
Rarefy + Wilcox	Rarefaction (TSS equivalent)			✗
GMPR + DESeq2	Geometric mean of pairwise ratios (GMPR)	Model (overdispersion)	Negative binomial model	✓
GMPR + edgeR	GMPR	Model (overdispersion)	Negative binomial model	✓
Wrench + MSeq	Wrench	Model (zero inflation)	Zero-inflated log-normal model	✗
RAIDA	Reference	Model (zero inflation)	Zero-inflated log-normal model	✗
ANCOM-BC	Bias correction	Pseudo-count	Log-linear model	✓
DACOMP	Reference	None	Wilcoxon rank-sum test	✗
LDM	TSS	None	Linear model	✓
Omnibus	GMPR	Model (zero inflation)	Zero-inflated negative binomial model	✗
Aldex2(Wilcox)	Centered log-ratio transformation (CLR)	Bayes	Wilcoxon rank-sum test	✗
Aldex2(glm)			Generalized linear model (GLM)	✓
GMPR + glm	GMPR	Model (overdispersion)	GLM (quasi-Poisson)	✓
Corncob	TSS	Model (overdispersion)	Beta-binomial model	✓
MaAsLin2	TSS	Pseudo-count	Log-linear model	✓
eBay(Wilcox)	CLR	Empirical Bayes	Wilcoxon rank-sum test	✗
ZicoSeq	Reference	Empirical Bayes	Linear model	✓

For all simulated datasets, taxa with prevalence less than 10% or the maximum proportion less 0.2% are excluded from testing as is usually done in practice. For consistency, all filtering steps in the evaluated methods are disabled, and the same preprocessed datasets are used as the input to all methods.

#### Performance evaluation

We evaluate the performance of DAA methods based on their ability to control for false positives and their power to detect the true positives after applying false discovery rate (FDR) control (BH procedure [52]) at the 5% target level. False-positive control was assessed based on the observed FDR, which is the false discovery proportion (FDP) averaged over 100 simulation runs (1000 simulation runs for the global null). Power was assessed based on the average true positive rate (TPR). FDP and TPR are defined as follows:

$$FDP=\frac{FP}{TP+ FP}, TPR=\frac{TP}{TP+ FN}$$

where FP, TP, and FN are the number of false positives, true positives, and false negatives, respectively. To facilitate assessment and interpretation, we use a scoring system to summarize the performance across settings (Table S3):

##### False-positive control scoring system

Observed FDR ∈ (0, 0.05), (0.05, 0.1), (0.1, 0.2), and (0.2, 1) scores 3 stars (***, blue), 2 stars (**, yellow), 1 star (*, red), and 0 star (“×”, gray), respectively. The total score is the number of stars the method receives for each setting.

##### Power scoring system

We rank the methods based on their average TPRs (higher rank, better power). The total score is the sum of the ranks for each setting.

##### Overall score

To produce an overall score, we first convert the total FDR and TPR scores into ranks (“TPR rank” and “FDR rank”) so that equal weights are put on false-positive control and power. These ranks are summed for each method to produce an “overall score.” The order of the methods displayed in the figures is based on the overall score.

#### Performance summary criteria

To have an overview of the performance of the evaluated DAA methods, we summarize the performance using different evaluation metrics (Fig. 6). For each metric, the performance of each method is considered either “good,” “intermediate,” or “poor” based on the criteria stated in Table S4. Stability is assessed based on the Spearman correlation of *p*-values for those common taxa when no filtering or strict filtering (prevalence < 40% or max proportion < 0.2%) was imposed.

### Evaluation of differential abundance analysis methods based on experimental datasets

The experimental datasets consist of 106 datasets retrieved from curatedMetagenomicData [53] (48 datasets), HMP16SData [54] (54 datasets), and others [17, 55] (4 datasets) (Table S5). For datasets with multiple groups, we split them into multiple two-group comparison datasets. Samples with less than 100 reads are excluded. Since most methods are sensitive to depth confounding, we rarefy the datasets if we detect a significant difference in sequencing depth between groups (Wilcoxon *p*-value < 0.05). Specifically, if the minimum sequencing depth of the dataset is larger than 30,000, we rarefy the dataset to its minimum depth; otherwise we rarefy to 30,000. Taxa with prevalence less than 10% or the maximum proportion less than 0.2% are excluded from testing to reduce the number of the tests. We apply the 16 DAA methods evaluated in simulations to these datasets, and taxa with FDR-adjusted *p*-values less than 0.05 are considered significant.

### ZicoSeq: an optimized procedure for differential abundance analysis

#### An omnibus F-statistic to capture diverse relationships between the covariates and the taxa abundance

Suppose the sequencing data consists of *n* samples and *m* taxa. Denote the *C*_*ij*_ (*i* = 1, ⋯, *n*; *j* = 1, ⋯, *m*) the observed count for taxon *j* in sample *i* and *N*_*i*_ = ∑_*j*_*C*_*ij*_ the number of total counts in sample *i*. Let *Y*_*ij*_ = *C*_*ij*_/*N*_*i*_ be the observed proportion for taxon *j* in sample *i*, and *μ*_*ij*_ is the true (unobserved) proportion. Let *X*_*n* × *p*_ the design matrix for the covariate(s) of interest and *Z*_*n* × *q*_ the design matrix for the covariates we need to adjust. For ease of notation, we assume the intercept is contained in *Z*. With some abuse of notation, we also use *X* and *Z* to represent the random variables. We assume the following linear model for taxon *j*:

$$g\left(\frac{\mu_{ij}}{\mu_i^C};\rho \right)={X}_i{\alpha}_j+{Z}_i{\beta}_j+{\varepsilon}_{ij}\left(\ i=1,\dots, n,j=1,\dots, m\right),$$

where *g* (; *ρ*) is a transformation function with a parameter *ρ*, which allows flexible modeling of the relationship between the taxa abundance and the covariates, and ${\mu}_i^c$ is the cumulative proportion of a reference set of taxa, which are assumed to be non-differential to *X*, *X*_*i*_, and *Z*_*i*_ are the *i*th row vector of the design matrices, *α*_*j*_ and *β*_*j*_ are the regression coefficients for *j*th taxa (column vectors), and *ε*_*ij*_ is the error term with mean 0. Here, we use the reference approach to address compositional effects, similar in spirit to the strategy used in DACOMP [36]. Under the linear model setup, we use the traditional *F*-statistic to assess the association between the taxon *j* and the covariate of interest:

$${F}_{\rho, j}=\frac{\left(\frac{y_{\rho, j}^T\left({H}_{X,Z}-{H}_Z\right){y}_{\rho, j}}{p}\right)}{\left(\frac{y_{\rho, j}^T\left(I-{H}_{X,Z}\right){y}_{\rho, j}}{n-p-q}\right)},$$

where ${y}_{\rho, j}=g\left(\frac{\mu_j}{\mu^c};\rho \right)$, *μ*_*j*_,and μ^c^ are column vectors for *μ*_*ij*_ and ${\mu}_i^C$across samples, *H*_*X*, *Z*_, *H*_*Z*_ are the projection matrices into the space spanned by (*X*, *Z*) and *Z*, respectively, and *I* is the identity matrix. Traditional models for taxa abundance data usually use a log link/transformation function, which implicitly assumes that the covariate has an exponential effect on the abundance. However, the log function puts too much weight on the rare taxa, whose measurements are subject to larger measurement errors. Moreover, in real scenarios, the actual relationship could be more complex than the log relationship, and the relationship could also be taxon-specific. We thus propose to use a power transformation function *g*(*x*; *ρ*) = *x*^*ρ*^, which is similar to the Box-Cox transformation [56], and could potentially capture a diverse relationship between the taxa abundance and the covariates by using different *ρs*. When *ρ* is extremely small, it approximates a log relationship. In order not to miss important associations by relying on a single power function, we could examine multiple power functions with different *ρs*. An omnibus *F*-statistic *F*_*o*, *j*_ can then be defined by taking the maximum of *F*-statistic for different *ρs*:

$${F}_{O,j}=\underset{\rho }{\max }\ \frac{\left(\frac{y_{\rho, j}^T\left({H}_{X,Z}-{H}_Z\right){y}_{\rho, j}}{p}\right)}{\left(\frac{y_{\rho, j}^T\left(I-{H}_{X,Z}\right){y}_{\rho, j}}{n-p-q}\right)}.$$

In the simulation, we used *ρ* = 0.5, which already produced satisfactory performance.

#### Permutation-based false discovery rate control preserving the correlation structure of the abundance data

Due to the use of multiple *ρ*s, the analytical distribution of *F*_*o*, *j*_ under the null is difficult to obtain. We propose to use a permutation-based false discovery rate (FDR) control procedure to identify significant taxa at a target FDR level. When there are covariates *Z*, permutation is not as straightforward as the case without *Z*. Multiple permutation strategies to account for *Z* have been compared in terms of type 1 error control and power [57]. Among these, the procedures by Freedman-Lane [58] and Smith [57] permutation strategies were found to be overall the best. Here, we use the Smith procedure [57], which can be described in the following basic steps:

- Regress *X* on *Z* to obtain the fitted values $\hat{X}$ and residuals $\hat{E}$.
- Permute the residuals $\hat{\mathrm{E}}$, denoted as ${\hat{E}}^b$ (*b* = 1, 2, ⋯, *B*), and add ${\hat{E}}^b$ to $\hat{X}$ to obtain *X*^*b*^.
- Calculate ${F}_{O,j}^b$ based on *X*^*b*^ and *Z*.

Since the permutation strategy does not use the abundance data, it effectively keeps the correlation structure of the taxa abundance data. For a given *F*_*O*, *j*_ cutoff, we estimate the FDR based on the permuted data sets. We select the *F*_*O*, *j*_ cutoff to achieve the desired FDR level using the steps below:

- Order *F*_*O*, *j*_ (large to small): *F*_*O*, (1)_, *F*_*O*, (2)_, …, *F*_*O*, (*m*)_
- Let ${F}_{O,k}^b$ be the omnibus *F*-statistic for taxon *k* in *b*th permutation (*k* = 1, …, *m*, *b* = 1, 2, ⋯, *B*).
- For a cutoff *F*_*O*, (*j*)_,we conservatively estimate the FDR by the following:
  $$\tilde{q}_{(j)}=\frac{\sum_{k,b}\#\left({F}_{O,k}^b\ge {F}_{O,(j)}\right)/B}{j}$$
- For a given FDR level α, we reject taxa with indices less than or equal to the following:

$${\mathrm{argmax}}_j\left(\tilde{q}_{(j)}\le \alpha \right)$$

#### Inference about the underlying true proportions using an empirical Bayes approach with an informative beta mixture prior

Instead of using an uninformative prior or a beta prior to infer the underlying true proportions, we propose to use an informative beta mixture prior as follows:

$${\mu}_{ij}\sim {\pi}_j Beta\left({a}_{j1},{b}_{j1}\right)+\left(1-{\pi}_j\right) Beta\left({a}_{j2},{b}_{j2}\right),i=1,\dots, n;j=1,\dots, m$$

for the underlying true proportions *μ*_ij_. The mixture model is motivated by the observation that some taxa show two modes in the abundance distribution [59]. The bimodal distribution could also result from a specific sampling scheme such as case-control design, where the cases and controls have different distributions. Moreover, excessive zeros could be efficiently modeled by using a mixture component close to 0. Even in those taxa with a unimodal distribution, the mixture distribution tends to fit the data better due to the increased modeling power with more parameters. With the mixture prior, we use the empirical Bayes (EB) approach to obtain the posterior distribution, from which we generate posterior samples. These posterior samples can then be used in the procedures stated above. The EB approach estimates the hyper-parameters of the mixture prior by maximizing the marginal likelihood of the data. Expectation-maximization (EM) algorithm can be used to obtain the estimates. With the hyper-parameter estimates ${\left({\hat{\pi}}_j,{\hat{a}}_{j1},{\hat{b}}_{j1},{\hat{a}}_{j2},{\hat{b}}_{j2}\right)}_{j=1,2,\dots m}$, we sample *μ*_*ij*_ from the posterior distribution:

$${\mu}_{ij}^{\ast}\sim {\hat{\pi}}_{ij} Beta\left({C}_{ij}+{\hat{a}}_{j1},{N}_i-{C}_{ij}+{\hat{b}}_{j1}\right)+\left(1-{\hat{\pi}}_{ij}\right) Beta\left({C}_{ij}+{\hat{a}}_{j2},{N}_i-{C}_{ij}+{\hat{b}}_{j2}\right),$$

where

$${\hat{\pi}}_{ij}=\frac{{\hat{\pi}}_j Beta\left({C}_{ij}+{\hat{a}}_{j1},{N}_i-{C}_{ij}+{\hat{b}}_{j1}\right)}{{\hat{\pi}}_j Beta\left({C}_{ij}+{\hat{a}}_{j1},{N}_i-{C}_{ij}+{\hat{b}}_{j1}\right)+\left(1-{\hat{\pi}}_j\right) Beta\left({C}_{ij}+{\hat{a}}_{j2},{N}_i-{C}_{ij}+{\hat{b}}_{j2}\right)}.$$

Figures S2 and 3 show the fit of the estimated beta mixture prior to the observed proportions for several representative taxa from two real datasets. We can see the beta mixture prior fits better than the beta prior based on the COMBO [60] (*n* = 98) and AGP [43] (*n* ≈ 10, 000) datasets.

The posterior inference of the underlying true proportions can be regarded as a new approach for normalization. When the sequencing depth is associated with the variable of interest, using the posterior proportions instead of the observed proportions reduces type 1 error inflation for rare taxa. Figure S4a shows the *p*-value distributions based on Wilcoxon rank-sum test (10,000 runs) using different normalization methods when a low-abundance taxon (0.4% relative abundance, 25% physical absence) is not differentially abundant between groups (*n* = 100), but the sequencing depth differs by tenfold (500 vs 5000). Our approach controls the type 1 error at the nominal level, similar to the rarefaction approach (Fig. S4a). In contrast, the test based on the observed proportions has severe type 1 error inflation. Using the beta prior reduces the type 1 error inflation but could not bring it down to the nominal level. Therefore, our posterior inference strategy addresses the sequencing depth variation effectively by exploiting the full distributional information in the data. On the other hand, the control for false positives does not affect the power much as shown in Fig. S4b when the abundance of the same taxon (0.4% relative abundance, 25% physical absence) increases by 25% in one group. As expected, our approach is more powerful than the rarefaction approach due to using more information in the data. For abundant taxa, the new approach does not inflate the type 1 error or significantly decreases the power. Figure S5 shows the *p*-value distributions for an abundant taxon (9% relative abundance, 25% physical absence) under the null (Fig. S5a) and the alternative (Fig. S5b).

We therefore use the posterior proportions instead of the observed proportions in *F*_*O*, *j*_. To reduce the variability, we draw *K* posterior samples (default: 25) and derive a new test statistic averaging over *F*_*O*, *j*_.

$${F^\ast}_{O,j}=\max\nolimits_\rho\frac1K\sum\nolimits_{k=1}^K\frac{\left(\frac{{y_{\rho,j}^k}^T\left(H_{X,Z}-H_Z\right)y_{\rho,j}^k}p\right)}{\left(\frac{{y_{\rho,j}^k}^T\left(I-H_{X,Z}\right)y_{\rho,j}^k}{n-p-q}\right)}.$$

where ${y}_{\rho, j}^k$ is defined based on the *k*th posterior sample.

#### Reference taxa selection based on the pairwise log ratios

Motivated by the idea of DACOMP [36] and ANCOM [18], we address the compositional effects using the reference taxa approach. The reference taxa are assumed to be less likely to be differential with respect to the covariate of interest. Based on the observation that the log ratios to other taxa for a differential taxon are mostly differential with respect to the covariate of interest while the log ratios for a non-differential taxon are mainly non-differential, we select the reference taxa based on pairwise log ratios. To accommodate the covariates *Z*, we regress each pairwise log ratio (add 1 to all counts to avoid 0 s) on *Z* using linear regressions and obtain the variance estimate for the error term. The error variances for those log ratios involving the differential taxa are expected to be larger than those non-differential taxa since the error term also contains the effect from the covariate of interest. For each taxon, we then take the median of the error variance estimates based on the log ratios to all other taxa and use the median statistic to rank the taxa. Finally, we select 50% taxa with the lowest error variances as the reference set. This approach uses a similar idea of DACOMP but is more flexible and can address covariates. To further improve the robustness of ZicoSeq for strong compositional effects, we exclude taxa with the lowest *p*-values (default: 20%) in the reference set and repeat running ZicoSeq for several iterations (default: 6). The 50% and 20% thresholds are determined empirically, and they generally lead to satisfactory performance in most settings. Some deviations from these two default thresholds only affect the results slightly (see Fig. S6, where we select 40% taxa with the lowest error variances as the reference set and further exclude 10% most significant taxa from the reference set in each iteration).

## Results

### A semiparametric simulation framework for realistic microbiome data generation

Our semiparametric framework starts with randomly drawing samples from a large reference dataset (e.g., data from the Human Microbiome Project (HMP)) [42], and these reference samples then serve as templates to generate new samples. For each reference sample, we infer its true composition, and the covariate/confounder effects are then added parametrically (“Methods”, Fig. S1). We compare the sample- and taxon-level characteristics of the microbiome data generated by our semiparametric approach to those by the Dirichlet-multinomial (DM) model. Sample-level characteristics are assessed by the percentage of zeros (sparsity), alpha diversity (Shannon diversity index), and β-diversity (Bray-Curtis distance). Taxon-level characteristics are assessed by taxa prevalence, mean and variance of the taxa relative abundance, and between-taxa correlation of the relative abundances.

For sample-level characteristics, the distribution of sample sparsity (Fig. S7a) of the simulated data by our semiparametric approach is close to that of the real dataset. In contrast, DM produces a significantly lower sparsity level suggesting DM tends to underestimate the sparsity. The distribution of the Shannon diversity index by the semiparametric approach also resembles that of the real data, while DM results in a slightly higher Shannon diversity index (Fig. S7b). We also compare the β-diversity (Bray-Curtis distance) of the simulated data to that of the real data based on the first two principal coordinates from principal coordinate analysis [61] (Fig. S7c). A clear overlap between the data simulated by the semiparametric approach and the real data indicates that the inter-sample relationship is well preserved by the proposed approach. In contrast, DM-simulated data lack the variability, and the distance between samples is significantly smaller than that in the real data.

For taxon-level characteristics, the distribution of the taxa prevalence in the simulated data by the semiparametric approach is similar to that of the real data, while the DM-simulated data has a slightly higher taxa prevalence (Fig. S7d). The semiparametric approach also captures well the distribution of the mean and variance of the taxa relative abundance observed in the real data (Fig. S7 e–f). In contrast, the corresponding distribution for the DM-simulated data significantly deviates from the real data, especially for vaginal data (Fig. S7e). Specifically, DM tends to overestimate the mean abundance for those less abundant taxa and underestimates the mean abundance for those abundant taxa (Fig. S8). Thus, DM-simulated data have a higher evenness, explaining a larger Shannon diversity index observed in the sample-level characteristics. In terms of the variance of the taxa relative abundance, the DM model severely underestimates the variance (Fig. S7f), indicating that a common dispersion parameter for all taxa is far from realistic. The heat map based on the taxa relative abundance data also shows a high similarity between the semiparametric approach-simulated data and the real data (Fig. S9). For between-taxa correlations of the relative abundances, the semiparametric approach largely preserves the correlation structure observed in real data; the distribution of the Spearman correlation coefficients shows a high agreement with that of the real data (Fig. S7g). However, the correlation structure in the DM-simulated data is very different, the range of the correlation coefficients is much narrower, and the distribution is almost symmetrical around 0 with slightly more negative values. For real data, the distribution of the correlation coefficients has more positive values for both the stool and vaginal data, and the distribution is bimodal for the vaginal data. Therefore, we conclude that semiparametric approach could capture both the first- (prevalence, mean, and variance) and second-order (correlation) characteristics observed in the real microbiome data.

### A benchmark study of differential abundance analysis methods using the semiparametric simulation framework

Next, we evaluate the performance of DAA methods using the proposed semiparametric simulation framework. We select methods from well-known labs and methods that have shown competitive performance. A total of 16 methods are included in the evaluation (Table 2, Table S2). We focus on the two-group comparison problem since all the DAA methods could be applied to this setting. To dissect DAA methods’ performance, we simulate data from both a high-diversity community (stool) and a low-diversity community (vaginal) and include three levels of signal densities (“low,” “medium,” “high”) and two differential modes (“abundant” and “rare”) depending on whether the differential taxa are relatively rare or abundant. To study the robustness of DAA methods to compositional effects, we simulate both “balanced” and “unbalanced” changes depending on whether the direction of change is random or the same. False-positive control (observed false discovery rate, FDR) and power (true positive rate, TPR) after false discovery rate (FDR) control at 5% level are used to measure the performance. The configurations of the studied settings are summarized in Table 1.

### Performance of differential abundance analysis methods under the global null setting

**Fig. 1:**
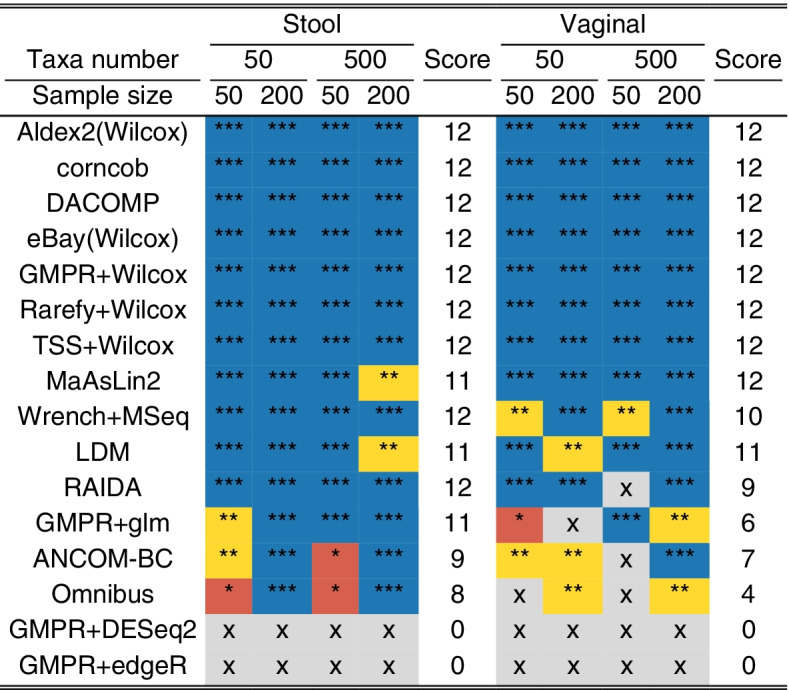
Performance of differential abundance analysis methods under the global null setting. Performance is assessed by the observed false discovery rate (FDR) level calculated as the percentage of the 1000 simulation runs making any false discoveries. The blue, yellow, red, and gray colors indicate the observed FDR level in (0, 0.5), (0.05–0.1), (0.1, 0.2), and (0.2, 1), respectively. Blue, yellow, red, and gray receive three (***), two (**), one (*), and zero (X) stars, respectively. The last column “score” indicates the total number of stars (*) each method receives

We first study the global null setting, where there are no differential taxa between the two groups (setting 1). In this case, FDR is equivalent to the family-wise error rate (FWER), which is the probability of making any false claims in multiple testing. We compare the FDR control of different DAA methods at 5% nominal level (Fig. 1, Fig. S10). For stool data, most methods could control the FDR close to the target level (Fig. 1, left, Fig. S10). Omnibus, ANCOM-BC, and GMPR + glm show some FDR inflation (5–20%) when the sample size is small (*n* = 50). In contrast, GMPR + edgeR and GMPR + DESeq2 could not control the FDR properly (FDR > 20%) (Fig. 1, Fig. S10). The false-positive control becomes even worse when the native normalization methods are used (RLE and TMM for DESeq2 and edgeR, respectively) (Fig. S11). For vaginal data, more methods fail to control the FDR within 20% (Fig. 1, right, Fig. S10). Wrench + MSeq, GMPR + glm, RAIDA, Omnibus, and ANCOM-BC all show decreased performance. Particularly, Omnibus and ANCOM-BC do not control the FDR for small sample sizes (*n* = 50), while GMPR + glm does not perform well when the taxa number is small (*m* = 50).

### Performance of differential abundance analysis methods under balanced changes

We next study the performance when there are differential signals between the groups (*n* = 100, *m* = 500, setting 2). We first evaluate the performance where the changes are balanced, i.e., the abundance of the differential taxa increases or decreases in one group randomly. In this setting, the compositional effects are considered to be very moderate since the effects of those differential taxa tend to balance out.

**Fig. 2:**
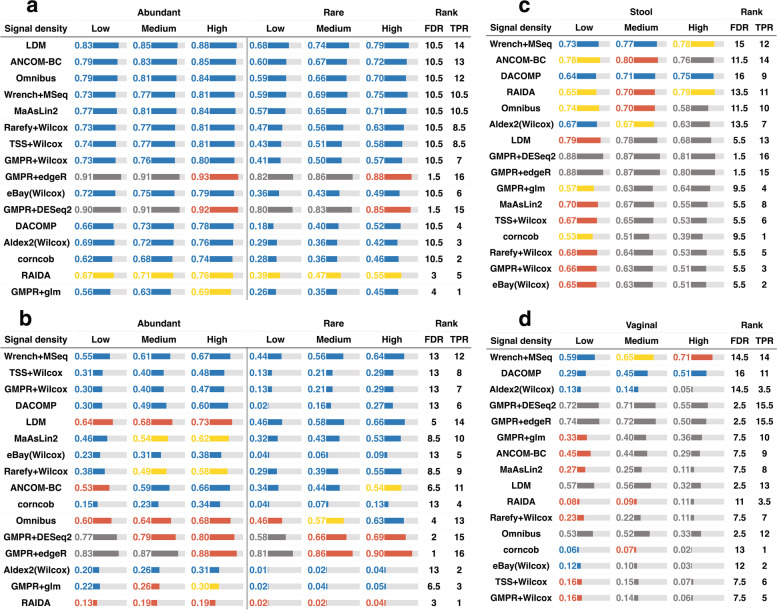
Performance of differential abundance analysis methods under the balanced change setting for **a** stool and **b** vaginal data and unbalanced change setting for **c** stool and **d** vaginal data (sample size = 100, taxa number = 500). Performance is assessed by the observed false discovery rate (FDR) level and average true positive rate (TPR). The color of the bar indicates the FDR control performance. The blue color indicates that the method controls the FDR at the 5% target level (the 95% confidence interval covers 5%). Yellow, red, and gray colors indicate the observed FDR level in (0.05–0.1), (0.1, 0.2), and (0.2, 1), respectively. The length of the bar is proportional to the TPR, and the actual TPR is shown in the bar. FDR and TPR ranks are based on the average FDR and TPR score across signal densities and differential modes. The order of the method is arranged based on the sum of the FDR and TPR ranks

For stool data, all the methods, except GMPR + edgeR, GMPR + DESeq2, and RAIDA, could control the FDR at the target level across signal densities and differential modes (Fig. 2a, Fig. S12a). In terms of statistical power, LDM is the most powerful, followed by ANCOM-BC, Omnibus, Wrench + MSeq, and MaAsLin2. The three variants of Wilcoxon rank-sum test with different normalization strategies (Rarefy + Wilcox, TSS + Wilcox, GMPR + Wilcox) perform equally well and are only slightly less powerful than the most powerful methods. In contrast, eBay(Wilcox), DACOMP, Aldex2(Wilcox), corncob, and GMPR + glm are less powerful especially when the differential taxa are rare.

For vaginal data, the FDR control performance decreases substantially for many methods (Fig. 2b, Fig. S12b). Although LDM, Omnibus, and ANCOM-BC remain powerful, FDR inflation has been observed under some settings, particularly when the differential taxa are abundant. In contrast, Wrench + MSeq is overall the best; it controls the FDR at the target level across settings, and the power is among the highest. GMPR + Wilcox, DACOMP, and TSS + Wilcox also control the FDR at the target level, but their power is very low for rare differential taxa. Interestingly, rarefaction (Rarefy + Wilcox) significantly improves the power to detect rare differential taxa, while the power to detect abundant differential taxa remains similar, indicating that rarefaction can reduce the variability in detection power due to uneven sequencing depth for those rare taxa.

### Performance of differential abundance analysis methods under unbalanced changes

When the compositional effects are moderate as in the balanced change scenario, most methods have satisfactory FDR control. Next, we study the performance of DAA methods under strong compositional effects (*n* = 100, *m* = 500) (setting 6). This is achieved by simulating unbalanced changes, i.e., the abundance of differential taxa increases in one group only and letting the differential taxa be relatively abundant. Such extreme scenarios may not be common in practice, but it could be used to test the limit of DAA methods.

For stool data, most methods do not control for false positives across signal densities, and the FDR control performance worsens as the signal becomes denser (Fig. 2c, Fig. S12c). For those methods based on TSS normalization or equivalent (LDM, MaAsLin2, TSS + Wilcox, Rarefy + Wilcox, GMPR + Wilcox, and eBay(Wilcox)), their FDR control is acceptable (< 20%) only when the signal density is low. In contrast, methods that explicitly address compositional effects (Aldex2(Wilcox): CLR, Omnibus/GMPR + glm: GMPR, Wrench + MSeq: Wrench, RAIDA/DACOMP: reference taxa, ANCOM-BC: bias correction) indeed have improved FDR control performance. However, as the signal density increases to 20%, only Wrench + MSeq, DACOMP, and RAIDA could control the FDR within a reasonable range (< 10%). Among these methods, DACOMP and Wrench + MSeq offer the strongest FDR control with DACOMP being the only method that controls the FDR across signal densities. Both Wrench + MSeq and DACOMP are powerful in this setting. For vaginal data, FDR control further deteriorates for most methods (Fig. 2d, Fig. S12d). Overall, Wrench + MSeq and DACOMP still outperform other methods. While DACOMP is less powerful than Wrench + MSeq, its FDR control performance is superior.

### Impact of the sample size and the number of taxa

For pilot microbiome studies, the sample size is usually small. It is interesting to see how the DAA methods perform when the sample size is small. We thus simulate datasets with a sample size of 50 (25 in each group) (settings 3 and 7). As we decrease the sample size, we see a significant decrease in power as expected (Fig. S13). When the changes are balanced, we see a significant decrease in FDR control performance for ANCOM-BC and Omnibus (Fig. S13 a–b). In contrast, LDM controls the FDR across signal densities and is the most powerful method for both stool and vaginal data. Wrench + MSeq also performs well, but the power is slightly lower than LDM for stool data. When the changes are unbalanced, Wrench + MSeq remains robust and powerful across settings and has overall the best performance (Fig. S13 c–d). While DACOMP controls the FDR at the target level across signal densities for both vaginal and stool data, its power for vaginal data is extremely low.

DAA has also been performed at higher taxonomic levels such as the family and genus level to identify clustered signals. As the number of analyzed taxa becomes smaller, the compositional effect becomes stronger. To study the impact of a small taxa number, we perform additional simulations by including only 50 most abundant taxa in DAA (settings 4 and 8, Fig. S14). When the changes are balanced, many methods have deteriorated FDR control performance, compared to their performance with 500 taxa (Fig. S14 a–b). FDR inflation is more severe when the differential taxa are abundant. In particular, LDM, Omnibus, and GMPR + glm could not control the FDR properly (> 20%) for vaginal data (Fig. S14b). The performance of Wrench + MSeq is not as remarkable in this setting; some FDR inflation has been observed for both stool and vaginal data, and the power is surprisingly low for stool data when the differential taxa are abundant. Overall, ANCOM-BC and Aldex2(Wilcox) are the two recommended methods in this setting. When the changes are unbalanced, FDR control becomes even more challenging (Fig. S14 c–d). For stool data, only RAIDA and ANCOM-BC could control the FDR under a reasonable level (no gray color, < 20%) when the signal density is high. Their power is also among the highest. For vaginal data, Wrench + MSeq, DACOMP, and RAIDA have the overall best FDR control performance. Among the three, DACOMP is the only method that controls the FDR at the target level across signal densities. In terms of power, Wrench + MSeq and DACOMP are substantially more powerful than RAIDA.

### ZicoSeq: an optimized procedure for differential abundance analysis of zero-inflated compositional sequencing data

According to the evaluation above, we found that none of the existing DAA methods is robust and powerful across settings. For example, those TSS-based methods such as TSS + Wilcox, Rarefy + Wilcox, LDM, MaAsLin2, and corncob do not control the FDR well under strong compositional effects, ANCOM-BC and Omnibus have severe FDR inflation under small sample sizes or strong compositional effects, and Aldex2(Wilcox) and DACOMP tend to be less powerful for rare differential taxa. Therefore, there is no optimal method that can be applied in all settings, and the best method depends on the specific setting. In practice, we do not know a priori which specific setting the real data belongs to. This makes the selection of the suitable DAA method difficult for end users. Although Wrench + MSeq is overall the most robust and powerful, it is not flexible; currently, it only supports two-group comparison and cannot adjust for covariates. This is a major drawback since microbiome studies are subject to many confounders [44–48], and confounder adjustment is necessary to reach a valid conclusion. In addition, some aberrant behavior has been noted under small numbers of taxa. Therefore, an optimized procedure to perform DAA is still highly desirable.

Based on the observation that DACOMP offers the best FDR control under strong compositional effects and LDM has the highest power when the compositional effect is moderate, we design an optimized procedure, ZicoSeq, drawing the respective strength of DACOMP and LDM. Specifically, we use a similar reference-based normalization strategy in DACOMP to address compositional effects and select the reference taxa based on pairwise log ratios. To perform association testing, we follow LDM by using a linear model-based permutation test. Permutation test, which assesses the statistical significance by permutations, depends on fewer assumptions and is expected to be more robust to model misspecification. To address zero inflations, we develop a new zero imputation method exploiting the full distributional information in the abundance data. The method assumes that the underlying proportion follows a beta mixture distribution and uses an empirical Bayes approach to draw posterior samples of the underlying true proportions (“Methods”). The test statistic is then averaged over the posterior samples.

### ZicoSeq robustly and powerfully detects differential taxa across settings

**Fig. 3:**
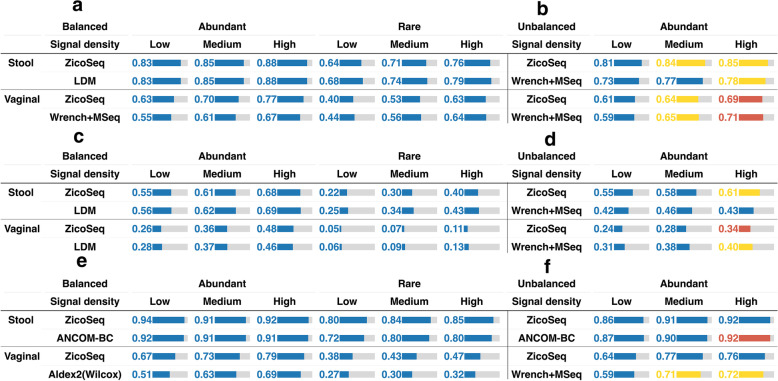
Comparison of ZicoSeq to the top-scoring method under different settings for stool and vaginal data. **a** Balanced and **b** unbalanced change setting (sample size = 100, taxa number = 500). **c** Balanced and **d** unbalanced change setting (sample size = 50, taxa number = 500). **e** Balanced and (f) unbalanced change setting (sample size = 100, taxa number = 50). Performance is assessed by the observed false discovery rate (FDR) level and average true positive rate (TPR). The color of the bar indicates the FDR control performance. The blue color indicates that the method controls the FDR at the 5% target level (the 95% confidence interval covers 5%). Yellow, red, and gray colors indicate the observed FDR level in (0.05–0.1), (0.1, 0.2), and (0.2, 1), respectively. The length of the bar is proportional to the TPR, and the actual TPR is shown in the bar

We apply ZicoSeq to the same simulated datasets used to evaluate the performance of existing DAA methods. For the global null setting, ZicoSeq effectively controls the FDR at the targeted level for both vaginal and stool data (Fig. S15). We then compare the performance of ZicoSeq to the top-ranking method in various differential settings (Fig. 3, Fig. S16). When the changes are balanced (Fig. 3a, Fig. S16a), ZicoSeq controls the FDR across settings for both stool and vaginal data. The power of ZicoSeq is similar to that of LDM, but ZicoSeq offers better FDR control than LDM for vaginal data when the differential taxa are abundant. When the changes are unbalanced (Fig. 3b, Fig. S16b), we do observe some FDR inflation for ZicoSeq, but the overall performance is comparable to Wrench + MSeq. When the sample size is small (*n* = 50), ZicoSeq remains as powerful as LDM when the changes are balanced and is comparable to Wrench + MSeq when the changes are unbalanced (Fig. 3 c–d, Fig. S16 c–d). A larger sample size (*n* = 1000) retains the same trend (Fig. S17). Remarkably, when the number of tested taxa is small (*m* = 50), ZicoSeq controls the FDR even under the unbalanced change setting, and its power is similar to the most powerful method (Fig. 3 e–f, Fig. S16 e–f). Based on these results, we conclude that ZicoSeq is more robust than existing methods, and its performance is always close to or slightly better than the best-performing method.

### ZicoSeq improves over existing methods in the presence of confounders

**Fig. 4:**
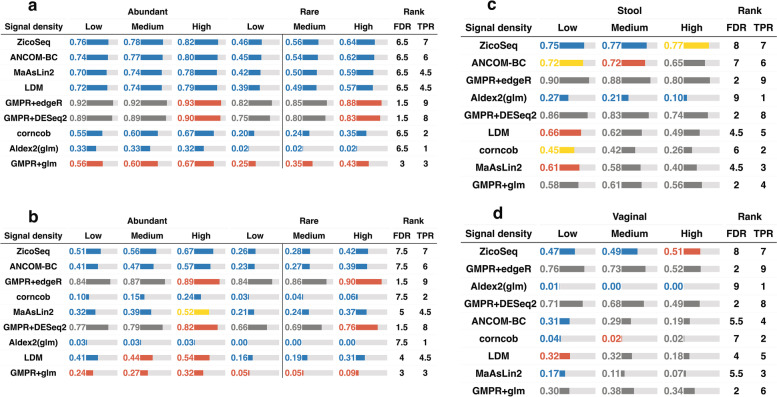
Performance of ZicoSeq in the presence of confounders under balanced change setting for **a** stool and **b** vaginal data and unbalanced change setting for **c** stool and **d** vaginal data (sample size = 100, taxa number = 500). Performance is assessed by the observed false discovery rate (FDR) level and average true positive rate (TPR) in comparison with methods capable of covariate adjustment. The color of the bar indicates the FDR control performance. The blue color indicates that the method controls the FDR at the 5% target level (the 95% confidence interval covers 5%). Yellow, red, and gray colors indicate the observed FDR level in (0.05–0.1), (0.1, 0.2), and (0.2, 1), respectively. The length of the bar is proportional to the TPR, and the actual TPR is shown in the bar. FDR and TPR ranks are based on the average FDR and TPR score across signal densities and/or differential modes. The order of the method is arranged based on the sum of the FDR and TPR ranks

Although all the existing DAA methods can be applied to the two-group comparison problem, some methods including Wrench + MSeq are unable to adjust covariates. ZicoSeq is based on linear models, and covariate adjustment is straightforward in its framework. We next compare the performance of ZicoSeq to those DAA methods capable of adjusting covariates when there are confounders. We simulate one continuous confounder (“Methods”), which is correlated with both the group membership and the abundances of a random subset of taxa (settings 9 and 10). We compare ZicoSeq to GMPR + DESeq2, GMPR + edgeR, GMPR + glm, Aldex2(glm), ANCOM-BC, corncorb, LDM, and MaAsLin2 (Fig. 4, Fig. S18). When the changes are balanced (Fig. 4 a–b, Fig. S18 a–b), most methods could control the FDR well, except GMPR + edgeR, GMPR + DESeq2, and GMPR + glm. Among methods that control the FDR, ZicoSeq, ANCOM-BC, MaAsLin2, and LDM are the most powerful for stool data, while ZicoSeq and ANCOM-BC are the most powerful for vaginal data. Aldex2(glm) and corncob, on the other hand, are much less powerful. For LDM and MaAsLin2, their performance deteriorates for vaginal data. When the changes are unbalanced (Fig. 4 c–d, Fig. S18 c–d), Aldex2(glm) has the best FDR control performance, but its power is extremely low. In contrast, ZicoSeq offers reasonable FDR control across settings for both stool and vaginal data and is substantially more powerful than Aldex2(glm). Other methods do not control FDR properly when the signal density is medium/high for both stool and vaginal data. Therefore, when there are confounders, ZicoSeq stands out among its competitors.

### ZicoSeq controls for false positives when the sequencing depth differs between groups

**Fig. 5:**
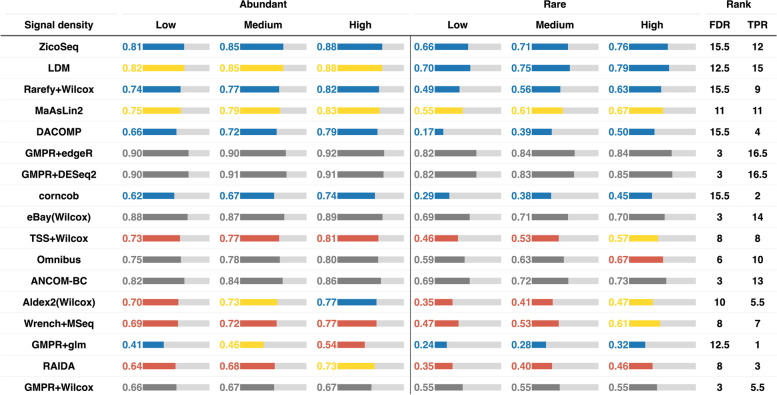
Performance of ZicoSeq when the sequencing depth differs by fourfold between the groups. The results are based on stool data under the balanced setting (sample size = 100, taxa number = 500). Performance is assessed by the observed false discovery rate (FDR) level and average true positive rate (TPR) in comparison with all evaluated methods. The color of the bar indicates the FDR control performance. The blue color indicates that the method controls the FDR at the 5% target level (the 95% confidence interval covers 5%). Yellow, red, and gray colors indicate the observed FDR level in (0.05–0.1), (0.1, 0.2), and (0.2, 1), respectively. The length of the bar is proportional to the TPR, and the actual TPR is also shown in the bar. FDR and TPR ranks are based on the average FDR and TPR score across signal densities and differential modes. The order of the method is arranged based on the sum of the FDR and TPR ranks

In microbiome sequencing, when the samples are not fully randomized, the sequencing depth likely differs between groups [15]. This can happen, for instance, when different groups of samples are placed on different sequencing plates. As the detection probability depends highly on the sequencing depth, such depth confounding could lead to potential false positives if not appropriately taken care of [49]. We thus simulate two groups of samples whose sequence depth differs by fourfold (setting 5). From Fig. 5 and Fig. S19, we can see that most evaluated methods have impaired FDR control in the presence of sequencing depth confounding. Rarefaction effectively controls FDR when Wilcox rank-sum test is used. DACOMP and corncob also control the FDR at the target level, while LDM and MaAsLin2 control the FDR within 10%. Other methods have severely inflated FDRs. When the sequencing depth difference increases to ninefold (Fig. S20), DACOMP starts to have inflated FDR. In contrast, Rarefy + Wilcox and corncob are still able to control the FDR at the target level. ZicoSeq, by using the new zero-imputation approach, effectively controls the FDR without the need for rarefaction, and its power is among the highest.

### Computational efficiency, stability, and performance summary

With the increasing scale of microbiome studies [42, 43], a computationally efficient DAA procedure is more likely to be adopted by the field. We thus compare the computational speeds of the evaluated DAA methods (Fig. S21). For the majority of the DAA methods, computation will not be a hurdle for their adoption. For a typical microbiome dataset (*n* = 100, *m* = 500), most of them can complete the analysis within 1 min on our computer system (×86_64-pc-linux-gnu (64 bit) Red Hat Enterprise Linux Server 7.9, Intel(R) Xeon(R) CPU E5-2698 v4 @ 2.20GHz, 8GB running memory), with LDM requiring longer computation than others (146.1s vs 1.2–57.8 s). For large sample sizes, ZicoSeq can complete the analysis at an average of 5 and 25 min for *n* = 1000 and 5000, respectively (Fig. S22). Based on the Green Algorithms (green-algorithms.org v2.1 [62]) and the geographic location of Minnesota, USA, ZicoSeq has a carbon footprint of 0.06 g CO2e, 0.59 g CO2e, and 3.16 g CO2e for *n* = 100, 1000, and 5000, respectively.

We also evaluate the stability of the DAA methods. Ideally, a stable DAA method should produce similar results regardless of the filtering criterion used, i.e., we would expect highly similar *p*-values for those common taxa regardless of whether we exclude 20% or 40% less prevalent taxa. To test for stability, we calculate the average Spearman correlation of the *p*-values based on two filtering criteria (0% vs 40% prevalence filtering) for each method. Most methods produce highly correlated *p*-values (mean Spearman *ρ* range: 0.93–1, Fig. S23) except DACOMP and RAIDA, which appear to be less stable than the other methods (mean Spearman *ρ*: 0.52 for both RAIDA and DACOMP).

**Fig. 6:**
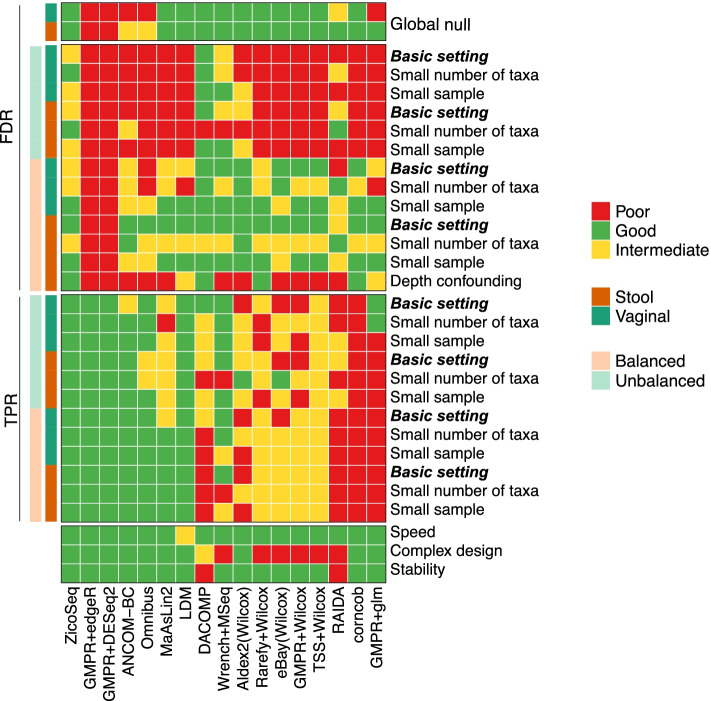
Performance summary of differential abundance analysis methods based on various evaluation metrics. The first and second parts include metrics for false-positive control (false discovery rate, FDR) and power (true positive rate, TPR), respectively. The third part consists of general metrics related to the usability of the method. For each metric, the performance is categorized into “poor,” “good,” and “intermediate” (Table S3). “Basic setting,” “small number of taxa,” and “small sample” refer to the setting with 100 samples and 500 taxa, 100 samples and 50 taxa, and 50 samples and 500 taxa, respectively

Finally, we summarize the DAA performance using different metrics based on our simulation studies (Fig. 6). For each evaluation metric, we classify each method as “good,” “intermediate,” or “poor” (Table S4). Although it is difficult to capture the full complexity of the evaluation based on a crude categorization, the heat map in Fig. 6 provides a convenient way to convey the major findings in the simulation studies. We can see that DACOMP offers the best FDR control, while LDM is among the most powerful. ZicoSeq, on the other hand, has overall the best performance; its FDR control is satisfactory across settings (no “red”), and the power is as high as LDM (all “blue”).

### Detection pattern on real datasets

It is informative to see how these methods perform on real datasets. We thus compare the evaluated DAA methods on 106 experiment datasets with binary outcomes collected from different sources (sample size range: 15–1688, taxa number range: 52–2281, Table S5). Since the ground truth is unknown, we focus on the detection pattern and to see if the pattern reflects what we have observed in simulations.

**Fig. 7:**
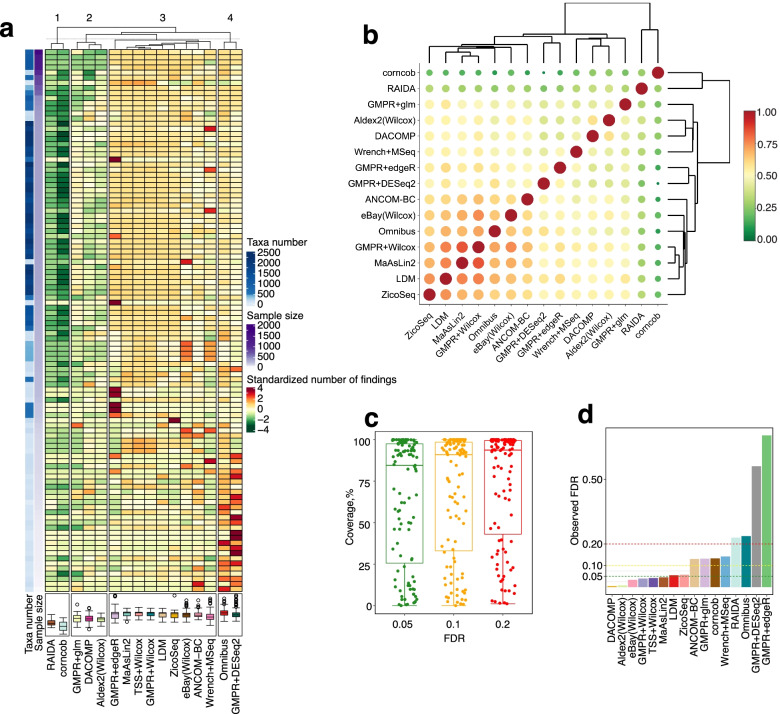
Evaluation of differential abundance analysis (DAA) methods based on 106 experimental datasets. **a** Heat map showing the numbers of significant taxa discovered by each DAA method in each dataset. Each row represents one dataset. The sidebars on the left show the sample size and taxa number for each dataset. The color scale for detection power is based on the standardized (scaled and mean centered) number of findings for each dataset. Datasets are hierarchically clustered based on Euclidean distance with the complete linkage. Box plots at the bottom show the distribution of the standardized number of findings across all datasets for each method. **b** Overlap of significant taxa (5% FDR) between DAA methods. Color and dot size indicate the percentage of overlap. Methods are hierarchically clustered based on Euclidean distance with the complete linkage. **c** The distribution of the percentage of taxa detected by at least one method at FDR 5%, 10%, and 20%. **d** The distribution of the observed false discovery rate (FDR) across the 106 real datasets when the group labels are randomly shuffled. The observed FDR level is calculated as the percentage of the 1000 repetitions making any false discoveries

We find that the number of differential taxa detected by DAA methods varies tremendously (Fig. 7a). Hierarchical clustering based on the number of detected differential taxa vaguely groups the 16 methods into 4 main groups. RAIDA, corncob, GMPR + glm, DACOMP, and Aldex2(Wilcox) (groups 1 and 2) tend to find less significant taxa than other methods, while GMPR + DESeq2 and Omnibus (group 4) are on the opposite side. The results are overall similar to those in the simulation studies, where we found that GMPR + DESeq2 is usually the most powerful among the evaluated methods, and RAIDA, corncob, GMPR + glm, DACOMP, and Aldex2(Wilcox) tend to be less powerful. Group 3 consists of the rest nine methods including ZicoSeq.

Next, we study the overlap of the significant taxa between methods across the 106 datasets (Fig. 7b). The average overlap with other methods ranges from 0.31 (RAIDA) to 0.58 (GMPR + Wilcox) at 5% FDR. We also see a cluster of methods, which have relatively large overlaps with each other (lower left corner). Overall, the overlaps are considered to be moderate, and it is expected that different methods will produce quite discordant results. The median percentages of taxa detected by at least one method are 85%, 91%, and 94%, for 5%, 10%, and 20% FDR, respectively (Fig. 7c). The surprisingly high coverage of taxa detected by at least one method raises concerns about potential cherry-picking if one does not declare the DAA tools they have tried in advance. It is very likely to find the taxa in favor of one’s hypothesis after trying out multiple DAA tools. Therefore, in order to increase the reproducibility of microbiome research, it is imperative for the field to have a consensus DAA tool, which is robust and powerful across settings so the end users could use it without the need for choosing the “best” tool themselves.

Finally, we evaluate the FDR control of DAA methods under the global null by shuffling the outcome labels for the 106 datasets. Using the 5% FDR cutoff, an ideal DAA method should control the FDR at or under that level. As a result, most methods perform well with a reasonable observed FDR and a small number of detected taxa (Fig. 7d, Fig. S24). However, GMPR + edgeR and GMPR + DESeq2 show the highest false-positive rates as indicated by larger numbers of significant taxa and highly observed FDRs. Among the rest methods, RAIDA and Omnibus have significantly elevated FDR levels, while Wrench + MSeq, corncob, GMPR + glm, and ANCOM-BC show slight inflation. These results generally agree with the simulation for the global null setting.

## Discussion

Differential abundance analysis (DAA) is one of the most fundamental statistical tools for microbiome data analysis [63]. Given the importance of this topic, numerous DAA tools have been proposed addressing the statistical challenges facing microbiome data such as zero inflation and compositional effects [17, 18, 24–26, 28, 31]. Recently, there have been a surge of new statistical methods including LDM, DACOMP, corncob, MaAsLin2, and ANCOM-BC. Although each method has demonstrated its superior performance to its predecessors using its own evaluation framework, it is unknown which method should be used in practice. Trying multiple DAA methods and selecting the method in favor of one’s own hypothesis increase the risk of false findings and reduce the reproducibility of the study [64]. Based on the 106 real datasets, we show that the median percentage of taxa detected at least by one DAA method (coverage) could be as high as 85% when the 5% target FDR level was used (Fig. 7c). Relaxing the target FDR level, the coverage can go even higher. For some datasets, the coverage could be 100%, meaning that one can always find a DAA tool, which declares a random taxon to be differential. Therefore, it is imperative for the field to reach some consensus about the optimal DAA tool or procedure. To achieve this goal, a comprehensive evaluation, which covers as many biologically relevant scenarios, is critically needed.

In this study, we performed a comprehensive assessment of the performance of the major existing DAA methods using the proposed semiparametric simulation framework. We show that the semiparametric simulation framework was able to recapitulate the essential sample- and taxon-level characteristics of the real data and was suitable for benchmarking the performance of DAA methods. Due to potential distinct characteristics of microbiome data from different sampling sites, we simulated data from both a high-diversity community (stool) and a low-diversity community (vaginal). To dissect the performance of DAA methods, we studied diverse simulation settings. We found that the false-positive control was still a major issue for most methods, especially when the compositional effects were strong and the community diversity was low. The two methods developed for RNA-Seq data, DESeq2, and edgeR had the worst FDR control and thus were not recommended for DAA. Those methods based on total sum scaling (TSS) such as MaAsLin2, corncob, and LDM were more susceptible to FDR inflation due to compositional effects. ANCOM-BC, Aldex2, and Omnibus test did improve over those TSS-based methods in FDR control, but their performance under strong compositional effects was still not satisfactory. Both ANCOM-BC and Omnibus test did not work well under a small sample size. Although DACOMP offered the best FDR control, its power was low under many settings, especially for rare taxa. metagenomeSeq with the wrench normalization controlled the FDR well across settings, and the power was also decent, but currently, it only supports two-group comparison, which limits its practical use in real data analysis. When the sequencing depth differed between groups, most methods failed to control the FDR, indicating rarefaction may be still needed for these methods. Based on the evaluation, we conclude that the existing methods still fall short of being simultaneously robust, powerful, and flexible, and each method only works under specific settings.

To obtain a list of highly confident differential taxa, one natural idea is to use ensembling, i.e., running multiple methods and using consensus to select the differential taxa. We explored the feasibility of this strategy by declaring differential taxa at different consensus levels (20%, 40%, 60%, and 80%). Figure S25 shows that the ensemble method still could not control the FDR under strong compositional effects unless a very high consensus level (80%) was used. However, in this case, the power was very low. Another idea is to select the best-performing method according to the data characteristics and potential signal structure (signal density, effect size, abundance of the affected taxa, and their direction of change). However, in practice, it is challenging to identify the specific setting where a DAA method is optimal.

We thus designed a new procedure, ZicoSeq, which draws on the strength of the existing methods, to meet the analysis needs. In the simulation, we found that DACOMP had the best FDR control under strong compositional effects, while LDM was generally the most powerful (Fig. 6). We thus adopted the reference-based approach (DACOMP) to address the compositional effects and a linear model-based permutation test (LDM) to conduct association testing. Different from the procedure in DACOMP, our reference-based approach could adjust for covariates when selecting the reference taxa. In the permutation test, we used the Smith permutation instead of the Freedman-Lane permutation as implemented in LDM for faster computation [57]. In addition, we proposed a novel zero imputation method based on beta mixture prior, exploiting the distributional characteristics of the abundance data. We show that ZicoSeq was overall more robust and powerful than existing methods; its FDR control and power were all close to or slightly better than the top-ranking method across settings. Therefore, microbiome researchers can apply ZicoSeq to their datasets without worrying about a potential high false-positive rate or low power of a specific method for their datasets. Our new zero-imputation method, which takes into account the sampling variability and sequencing depth variation, provides a new way of addressing excessive zeros. In the presence of depth confounding, ZicoSeq was the only method that could control the FDR at the target level while maintaining high power without the need for rarefaction. ZicoSeq is also flexible. Due to the use of linear models, covariate adjustment in ZicoSeq is straightforward. ZicoSeq also allows omnibus testing by using different transformations of the abundance data. Omnibus testing may improve the power when there are diverse relationships between the differential taxa and the covariate of interest. It will be an interesting research topic to determine the appropriate transformation functions for a specific dataset. The permutation-based FDR control procedure in ZicoSeq keeps the correlation structure among the taxa abundance data during permutations and thus is adaptive to the correlation structure in the data. The traditional BH-based FDR control, on the other hand, assumes independence among the hypotheses and is shown to be conservative when there are positive correlations [65]. Although the posterior sampling and permutation are used, ZicoSeq is still computationally efficient; it could complete the analysis of a typical dataset (e.g., *n* = 100, *m* = 500) within seconds.

There are limitations for ZicoSeq. First, the beta mixture-based imputation procedure was implemented for each taxon and did not impose the sum-to-one constraint by jointly considering all taxa. Although this simple approach works well in practice, a more sophisticated method, which considers the compositional constraint, may further improve the imputation performance. Second, mild FDR inflation was still observed when the compositional effects were strong. To design a better reference selection strategy or come up with a new way to address compositional effects is an interesting direction to pursue. Third, the current implementation does not consider the phylogenetic relatedness among the taxa. Phylogenetically related taxa usually share biological traits, and their association pattern with the covariate of interest is expected to be similar [66–68]. Such prior knowledge may be leveraged to improve the power of ZicoSeq as demonstrated in our phylogeny-based FDR control procedure [69]. Fourth, due to the use of data transformation and permutation, ZicoSeq is not as interpretable as those parametric methods, whose coefficient can usually be interpreted as the log fold change in response to one unit change of the covariate. Finally, the current implementation can only be applied to independent samples. Given the increasing popularity of longitudinal microbiome studies and studies involving repeated measurements, correlated microbiome data are now prevalent [70]. Thus, a DAA tool for correlated microbiome data is highly desirable.

During the review of the manuscript, several new methods for microbiome differential abundance testing methods were published including LinDA [71], fastANCOM [72], and ZINQ [73]. It is thus interesting to compare ZicoSeq to these methods. Fig. S26 summarizes the results under Settings 2&6 (Table 1). We can see that ZicoSeq still has a competitive edge over these methods. 

In conclusion, the problem of differential abundance analysis of microbiome data still has not been fully solved by existing methods. To meet the analytical needs and improve the reproducibility of microbiome research, we present a more robust and powerful procedure for differential abundance analysis.

## Conclusions

We performed the most comprehensive benchmarking study of DAA methods to date and found that none of the DAA methods was simultaneously robust, powerful, and flexible. The applicability of an existing DAA method depends on specific settings, which are usually unknown a priori. To circumvent the difficulty of selecting the best DAA tool that suits one’s dataset, we develop ZicoSeq, which remedies the drawbacks of existing methods. ZicoSeq can be applied to DAA of microbiome data from diverse settings.

## Supplementary Information

**Additional file 1: Figure S1**. Basic steps of the proposed semiparametric simulation framework. **Figure S2**. The fit of the estimated beta mixture prior for several representative taxa in the COMBO (*n* = 98) dataset in comparison to the beta prior. **Figure S3**. The fit of the estimated beta mixture prior for several representative taxa in the American Gut Project (≈ 10, 000) dataset. **Figure S4**. *P*-value distributions based on 10,000 simulation runs (a) when the abundance of a rare taxon (0.4% relative abundance, 25% physical absence) is the same between two groups, (b) when the abundance of the same taxon (0.4% relative abundance, 25% physical absence) increases by 25% in one group. **Figure S5**. *P*-value distributions based on 10,000 simulation runs (a) when the abundance of an abundant taxon (9% relative abundance, 25% physical absence) is the same between two groups, (b) when the abundance of the same taxon (9% relative abundance, 25% physical absence) increases by 25% in one group. **Figure S6**. Comparison of the FDR control and power using different thresholds to select the reference set under (a) balanced and (b) unbalanced settings (settings 9 and 10 shown in Table 2). **Figure S7**. Comparison of sample- and taxon-level characteristics between the semiparametric approach and Dirichlet-multinomial (DM) model simulated data. **Figure S8**. Dirichlet-multinomial model tends to (a) overestimate the mean abundance for those less abundant taxa and (b) underestimate the mean abundance of those abundant taxa in vaginal data. **Figure S9**. Heat maps showing the relative abundance data generated by Dirichlet-multinomial model and the proposed semiparametric approach, in comparison to the real data for (a) stool and (b) vaginal. **Figure S10**. Performance of differential abundance analysis methods under the global null setting, visualized using bar plots corresponding to Fig. 1. **Figure S11**. Performance comparison of DESeq2 and edgeR using its native normalization method (RLE and TMM) and the GMPR normalization under the global null setting. **Figure S12**. Performance of differential abundance analysis methods under the balanced change setting for (a) stool and (b) vaginal data, and unbalanced change setting for (c) stool and (d) vaginal data (sample size = 100, taxa number = 500), visualized using bar plots corresponding to Fig. 2. **Figure S13**. Performance of differential abundance analysis methods under a small sample size (sample size = 50, taxa number = 500). (a) Balanced change setting, stool data. (b) Balanced change setting, vaginal data. (c) Unbalanced change setting, stool data. (d) Unbalanced change setting, vaginal data. **Figure S14**. Performance of differential abundance analysis methods under a small number of taxa (sample size = 100, taxa number = 50). **Figure S15**. Performance of ZicoSeq under the global null setting for stool and vaginal data with different numbers of samples and taxa. **Figure S16**. Comparison of ZicoSeq to the top-scoring method under different settings for stool and vaginal data. (a) Balanced and (b) unbalanced change setting (sample size = 100, taxa number = 500). (c) Balanced and (d) unbalanced change setting (sample size = 50, taxa number = 50). **Figure S17**. Comparison of ZicoSeq to the top-scoring method in Fig. S16ab under different settings for stool and vaginal data at the sample size of 1000. **Figure S18**. Performance of ZicoSeq in the presence of confounders under balanced change setting for (a) stool, (b) vaginal data, and unbalanced change setting for (c) stool, (d) vaginal data (sample size = 100, taxa number = 500), visualized using bar plots corresponding to Fig. 4. **Figure S19**. Performance of ZicoSeq when the sequencing depth differs by 4-fold between the groups, visualized using bar plots corresponding to Fig. 5. **Figure S20**. Performance of ZicoSeq when the sequencing depth differs by 9-fold between the groups. **Figure S21**. Run times (x86_64-pc-linux-gnu (64-bit) Red Hat Enterprise Linux Server 7.9, Intel(R) Xeon(R) CPU E5-2698 v4 @ 2.20GHz, 8GB running memory) of the evaluated differential abundance analysis methods over simulation runs (unbalanced setting, vaginal data, 100 samples and 500 taxa). **Figure S22**. Run times (x86_64-pc-linux-gnu (64-bit) Red Hat Enterprise Linux Server 7.9, Intel(R) Xeon(R) CPU E5-2698 v4 @ 2.20GHz, 8GB running memory) of ZicoSeq over simulation runs when sample size increases to 1000 and 5000 (unbalanced setting, vaginal data, 500 taxa). **Figure S23**. Box plots showing the distribution of Spearman correlation of *p*-values between no filtered datasets and filtered datasets (prevenance less than 40% or minimal abundance less than 0.002 are excluded for analysis) based on unbalanced change setting for vaginal data. **Figure S24**. The average percentage of significant taxa at 5% FDR of the 106 real datasets when the group labels are randomly shuffled. **Figure S25**. Ensemble methods at a consensus level of 20%, 40%, 60% and 80% (denoted as “pct20”, “pct40”, “pct60” and “pct80”). **Figure S26**. Performance comparison to recently developed methods - LinDA, fastANCOM and ZINQ under settings 2&6. (a) Balanced, stool, (b) Balanced, vaginal, (c) Unbalanced Stool, (d) Unbalanced, vaginal. **Table S1**. Normalization methods reviewed in this study. **Table S2**. Package version and source link for the differential abundance analysis methods evaluated in this study. **Table S3**. Performance scoring system. **Table S4**. The evaluation metrics used in the performance summary. **Table S5**. Details of the experimental datasets

## Data Availability

The datasets and codes supporting the conclusions of this article are available in the https://github.com/chloelulu/DAA repository. The semiparametric simulation approach and the ZicoSeq procedure are implemented as “SimMSeq” and “ZicoSeq” function, respectively, in the CRAN *GUniFrac* package (https://CRAN.R-project.org/package=GUniFrac). All analyses are performed in R v4.0.3 on a ×86_64-pc-linux-gnu (64 bit) Red Hat Enterprise Linux Server 7.9 at the Mayo Clinic.
