# Supplementary material for: A comprehensive evaluation of microbial differential abundance analysis methods: current status and potential solutions
Source: Microbiome. 2022 Aug 19;10:130. doi: 10.1186/s40168-022-01320-0 (PMC9392415; doi:10.1186/s40168-022-01320-0)
Supplement: Supplementary file 2 — Additional file 1: Figure S1. Basic steps of the proposed semiparametric simulation framework. Figure S2. The fit of the estimated beta mixture prior for several representative taxa in the COMBO (n = 98) dataset in comparison to the beta prior. Figure S3. The fit of the estimated beta mixture prior for several representative taxa in the American Gut Project (≈ 10, 000) dataset. Figure S4. P-value distributions based on 10,000 simulation runs (a) when the abundance of a rare taxon (0.4% relative abundance, 25% physical absence) is the same between two groups, (b) when the abundance of the same taxon (0.4% relative abundance, 25% physical absence) increases by 25% in one group. Figure S5. P-value distributions based on 10,000 simulation runs (a) when the abundance of an abundant taxon (9% relative abundance, 25% physical absence) is the same between two groups, (b) when the abundance of the same taxon (9% relative abundance, 25% physical absence) increases by 25% in one group. Figure S6. Comparison of the FDR control and power using different thresholds to select the reference set under (a) balanced and (b) unbalanced settings (settings 9 and 10 shown in Table 2). Figure S7. Comparison of sample- and taxon-level characteristics between the semiparametric approach and Dirichlet-multinomial (DM) model simulated data. Figure S8. Dirichlet-multinomial model tends to (a) overestimate the mean abundance for those less abundant taxa and (b) underestimate the mean abundance of those abundant taxa in vaginal data. Figure S9. Heat maps showing the relative abundance data generated by Dirichlet-multinomial model and the proposed semiparametric approach, in comparison to the real data for (a) stool and (b) vaginal. Figure S10. Performance of differential abundance analysis methods under the global null setting, visualized using bar plots corresponding to Fig. 1. Figure S11. Performance comparison of DESeq2 and edgeR using its native normalization method (RLE and TMM) and the GMPR [file 40168_2022_1320_MOESM2_ESM.pdf]

Supplementary Figures and Tables for:

A comprehensive evaluation of microbial differential abundance analysis methods: current status and potential solutions

Lu Yang[1,2], Jun Chen[1,2*]

[1]Division of Computational Biology, Department of Quantitative Health Sciences, Mayo Clinic, Rochester, MN, 55905, U.S.A.

[2]Center for Individualized Medicine, Mayo Clinic, Rochester, MN, 55905, U.S.A.

Correspondence to Jun Chen (chen.jun2@mayo.edu).

Fig. S1 Basic steps of the proposed semiparametric simulation framework.

Fig. S2 The fit of the estimated beta mixture prior for several representative taxa in the COMBO (n = 98) dataset in comparison to the beta prior. The parameters of the beta mixture or beta distribution are estimated using maximum likelihood estimation assuming a binomial distribution of the count given the underlying proportion.

Fig. S3 The fit of the estimated beta mixture prior for several representative taxa in the American Gut Project ($\approx 10,000$) dataset. The parameters of the beta mixture or beta distribution are estimated using maximum likelihood estimation assuming a binomial distribution of the count given the underlying proportion.

Fig. S4 P-value distributions based on 10,000 simulation runs (a) when the abundance of a rare taxon (0.4% relative abundance, 25% physical absence) is the same between two groups, (b)

when the abundance of the same taxon (0.4% relative abundance, 25% physical absence) increases by 25% in one group.

Fig. S5 P-value distributions based on 10,000 simulation runs (a) when the abundance of an abundant taxon (9% relative abundance, 25% physical absence) is the same between two groups, (b) when the abundance of the same taxon (9% relative abundance, 25% physical absence) increases by 25% in one group.

Fig. S6 Comparison of the FDR control and power using different thresholds to select the reference set under (a) balanced and (b) unbalanced settings (settings 9 and 10 shown in Table 2). In the figure, "ref.pct 50%/ excl.pct 20%" (default thresholds) refers to the method using 50% taxa with the lowest error variances as the reference set and further excluding 20% taxa with the lowest p-values in the reference set in each iteration. "ref.pct 40%/ excl.pct 10%" refers to the method using 40% taxa with the lowest error variances as the reference set and further excluding 10% taxa with the lowest p-values in the reference set in each iteration. Error bars are mean±1.96×standard error.

Fig. S7 Comparison of sample- and taxon-level characteristics between the semiparametric approach and Dirichlet-multinomial (DM) model simulated data. The assessment consists of (a) the percentage of zeros (sparsity), (b) alpha diversity (Shannon diversity index), (c) β-diversity (Bray-Curtis distance), (d) the prevalence of taxa, (e) mean relative abundance of taxa, (f) variance of the relative abundance, and (g) between-taxa Spearman correlation of the relative abundances. In (ab, d-g), the histograms show the probability distributions, and the scatter plots compare the quantiles between the simulated data and real data. In (c), the two principal

coordinates (PCs) are generated via principal coordinate analysis based on the Bray-Curtis distance.

Fig. S8 Dirichlet-multinomial model tends to (a) overestimate the mean abundance for those less abundant taxa and (b) underestimate the mean abundance of those abundant taxa in vaginal data.

Fig. S9 Heat maps showing the relative abundance data generated by Dirichlet-multinomial model and the proposed semiparametric approach, in comparison to the real data for (a) stool and (b) vaginal.

Fig. S10 Performance of differential abundance analysis methods under the global null setting, visualized using bar plots corresponding to Fig. 1. Performance is assessed by the observed false discovery rate (FDR) level calculated as the percentage of the 1,000 simulation runs making any false discoveries. Error bars are mean±1.96×standard error.

Fig. S11 Performance comparison of DESeq2 and edgeR using its native normalization method (RLE and TMM) and the GMPR normalization under the global null setting.

Fig. S12 Performance of differential abundance analysis methods under the balanced change setting for (a) stool and (b) vaginal data, and unbalanced change setting for (c) stool and (d) vaginal data (sample size = 100, taxa number = 500), visualized using bar plots corresponding to Fig. 2. Error bars are mean±1.96×standard error. "Low", "Medium" and "High" refer to the signal densities, and "Abundant" and "Rare" refer to the differential mode (Table 1).

Fig. S13 Performance of differential abundance analysis methods under a small sample size (sample size = 50, taxa number = 500). (a) Balanced change setting, stool data. (b) Balanced change setting, vaginal data. (c) Unbalanced change setting, stool data. (d) Unbalanced change

setting, vaginal data. Performance is assessed by the observed false discovery rate (FDR) level and average true positive rate (TPR) in comparison to evaluated methods. The color of the bar indicates the FDR control performance. The blue color indicates that the method controls the FDR at the 5% target level (the 95% confidence interval covers 5%). Yellow, red and gray colors indicate the observed FDR level in (0.05-0.1], (0.1, 0.2], and (0.2, 1], respectively. The length of the bar is proportional to the TPR and the actual TPR is shown in the bar. FDR and TPR ranks are based on the average FDR and TPR score across signal densities and/or differential modes. The order of the method is arranged based on the sum of the FDR and TPR ranks.

Fig. S14 Performance of differential abundance analysis methods under a small number of taxa (sample size = 100, taxa number = 50). (a) Balanced change setting, stool data. (b) Balanced change setting, vaginal data. (c) Unbalanced change setting, stool data. (d) Unbalanced change setting, vaginal data. Performance is assessed by the observed false discovery rate (FDR) level and average true positive rate (TPR) in comparison to evaluated methods. The color of the bar indicates the FDR control performance. The blue color indicates that the method controls the FDR at the 5% target level (the 95% confidence interval covers 5%). Yellow, red and gray colors indicate the observed FDR level in (0.05-0.1], (0.1, 0.2], and (0.2, 1], respectively. The length of the bar is proportional to the TPR and the actual TPR is shown in the bar. FDR and TPR ranks are based on the average FDR and TPR score across signal densities and/or differential modes. The order of the method is arranged based on the sum of the FDR and TPR ranks.

Fig. S15 Performance of ZicoSeq under the global null setting for stool and vaginal data with different numbers of samples and taxa. Performance is assessed by the observed false discovery rate (FDR) level calculated as the percentage of the 1,000 simulation runs making any false discoveries. Error bars indicate the 95% confidence interval.

Fig. S16 Comparison of ZicoSeq to the top-scoring method under different settings for stool and vaginal data. (a) Balanced and (b) unbalanced change setting (sample size = 100, taxa number = 500). (c) Balanced and (d) unbalanced change setting (sample size = 50, taxa number = 50). (e) Balanced and (f) unbalanced change setting (sample size = 100, taxa number = 50), visualized using bar plots corresponding to Fig. 3. Error bars are mean±1.96×standard error. "Low", "Medium" and "High" refer to the signal densities, and "Abundant" and "Rare" refer to the differential modes (Table 1).

Fig. S17 Comparison of ZicoSeq to the top-scoring method in **Fig. S16ab** under different settings for stool and vaginal data at the sample size of 1000. (a-b) Balanced and (c-d) unbalanced change setting (sample size = 1000, taxa number = 500). Error bars are mean±1.96×standard error. "Low", "Medium" and "High" refer to the signal densities, and "Abundant" and "Rare" refer to the differential modes (Table 1).

Fig. S18 Performance of ZicoSeq in the presence of confounders under balanced change setting for (a) stool, (b) vaginal data, and unbalanced change setting for (c) stool, (d) vaginal data (sample size = 100, taxa number = 500), visualized using bar plots corresponding to Fig. 4. Error bars are mean±1.96×standard error. "Low", "Medium" and "High" refer to the signal densities, and "Abundant" and "Rare" refer to the differential modes (Table 1).

Fig. S19 Performance of ZicoSeq when the sequencing depth differs by 4-fold between the groups, visualized using bar plots corresponding to Fig. 5. The results are based on stool data under the balanced setting (sample size = 100, taxa number = 500). Error bars are mean±1.96×standard error. "Low", "Medium" and "High" refer to the signal densities, and "Abundant" and "Rare" refer to the differential modes (Table 1).

Fig. S20 Performance of ZicoSeq when the sequencing depth differs by 9-fold between the groups. The results are based on stool data under the balanced setting (100 samples and 500 taxa). Performance is assessed by the observed false discovery rate (FDR) level and average true positive rate (TPR) in comparison to evaluated methods. The color of the bar indicates the FDR control performance. The blue color indicates that the method controls the FDR at the 5% target level (the 95% confidence interval covers 5%). Yellow, red and gray colors indicate the observed FDR level in (0.05-0.1], (0.1, 0.2], and (0.2, 1], respectively. The length of the bar is proportional to the TPR and the actual TPR is also shown in the bar. FDR and TPR ranks are based on the average FDR and TPR score across signal densities and differential modes. The order of the method is arranged based on the sum of the FDR and TPR ranks.

Fig. S21 Run times (x86_64-pc-linux-gnu (64-bit) Red Hat Enterprise Linux Server 7.9, Intel(R) Xeon(R) CPU E5-2698 v4 @ 2.20GHz, 8GB running memory) of the evaluated differential abundance analysis methods over simulation runs (unbalanced setting, vaginal data, 100 samples and 500 taxa).

Fig. S22 Run times (x86_64-pc-linux-gnu (64-bit) Red Hat Enterprise Linux Server 7.9, Intel(R) Xeon(R) CPU E5-2698 v4 @ 2.20GHz, 8GB running memory) of ZicoSeq over simulation runs when sample size increases to 1000 and 5000 (unbalanced setting, vaginal data, 500 taxa).

Fig. S23 Box plots showing the distribution of Spearman correlation of p-values between no filtered datasets and filtered datasets (prevenance less than 40% or minimal abundance less than 0.002 are excluded for analysis) based on unbalanced change setting for vaginal data.

Fig. S24 The average percentage of significant taxa at 5% FDR of the 106 real datasets when the group labels are randomly shuffled.

Fig. S25 Ensemble methods at a consensus level of 20%, 40%, 60% and 80% (denoted as "pct20", "pct40", "pct60" and "pct80"). Performance is assessed by the observed false discovery rate (FDR) level and average true positive rate (TPR). The color of the bar indicates the FDR control performance. The blue color indicates that the method controls the FDR at the 5% target level (the 95% confidence interval covers 5%). Yellow, red and gray colors indicate the observed FDR level in (0.05-0.1], (0.1, 0.2], and (0.2, 1], respectively. The length of the bar is proportional to the TPR and the actual TPR is also shown in the bar.

Fig. S26 Performance comparison to recently developed methods - LinDA, fastANCOM and ZINQ under settings 2&6. (a) Balanced, stool, (b) Balanced, vaginal, (c) Unbalanced Stool, (d) Unbalanced, vaginal.

Table S1 Normalization methods reviewed in this study.

Table S2 Package version and source link for the differential abundance analysis methods evaluated in this study.

Table S3 Performance scoring system.

Table S4 The evaluation metrics used in the performance summary.

Table S5 Details of the experimental datasets.

Fig. S1

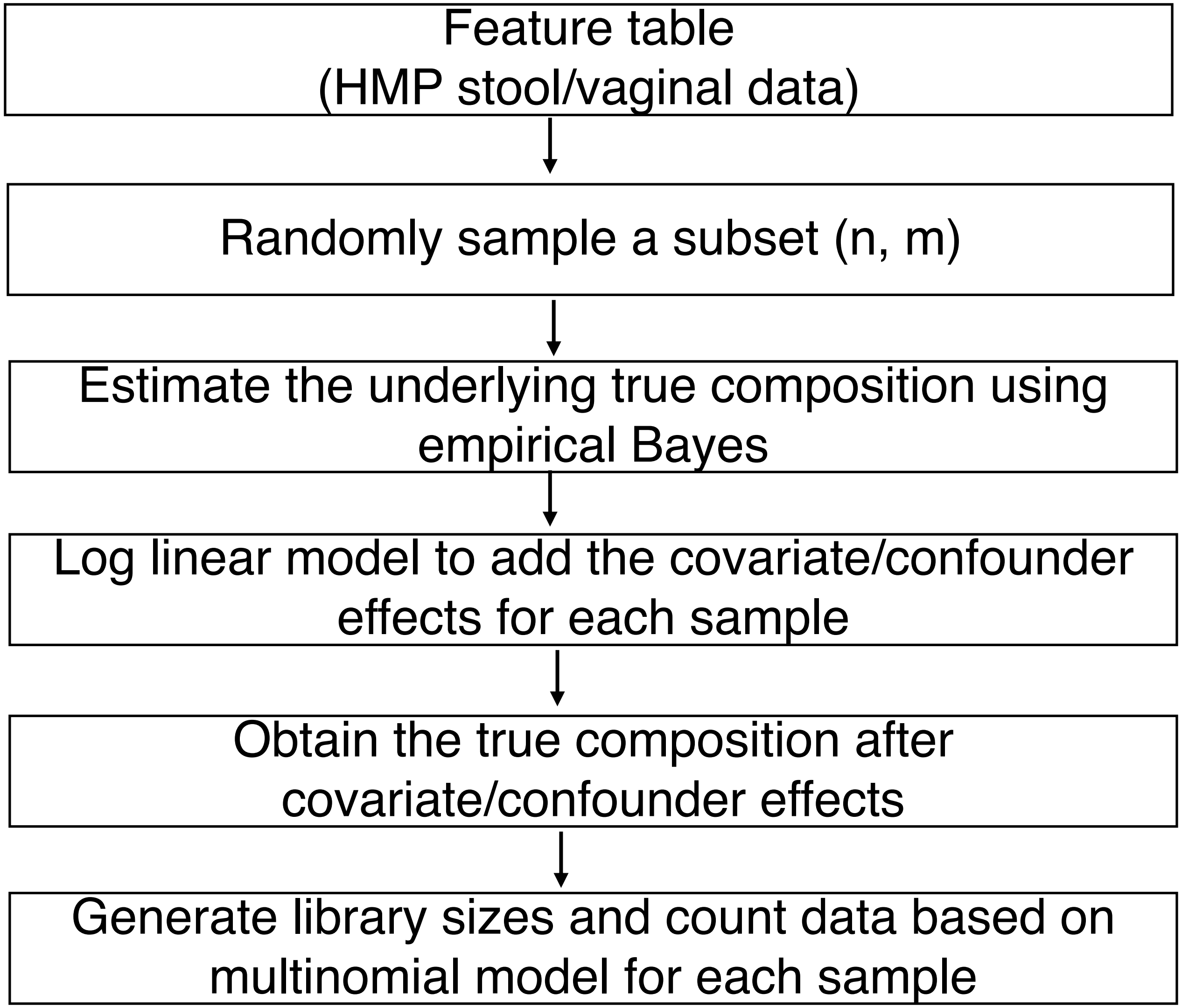
Feature table
(HMP stool/vaginal data)
Randomly sample a subset (n, m)
Estimate the underlying true composition using empirical Bayes
Log linear model to add the covariate/confounder effects for each sample
Obtain the true composition after covariate/confounder effects
Generate library sizes and count data based on multinomial model for each sample

Fig. S2

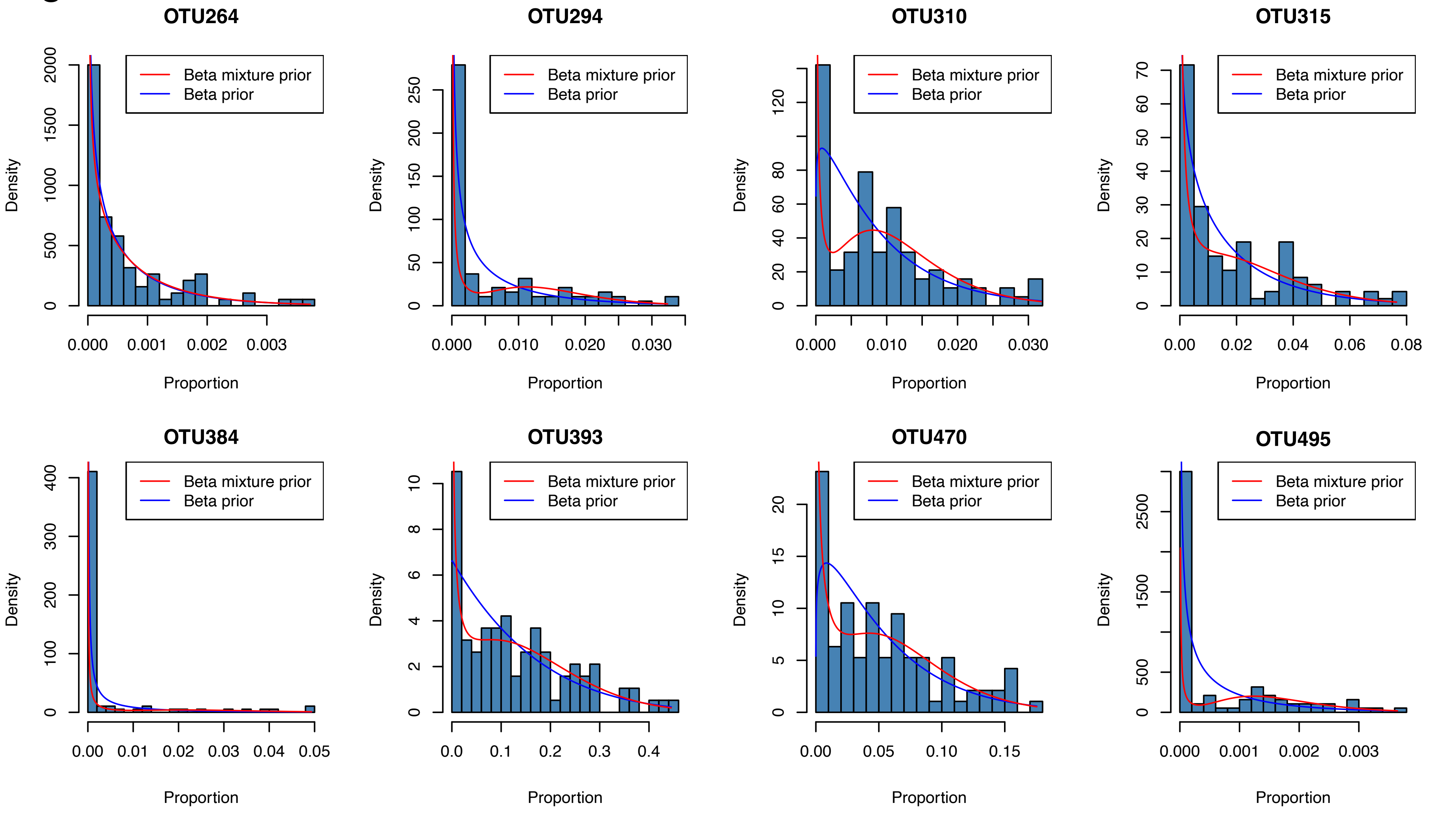
OTU264
OTU294
OTU310
OTU315
OTU384
OTU393
OTU470
OTU495
Beta mixture prior
Beta prior
Density
Proportion

Fig. S3

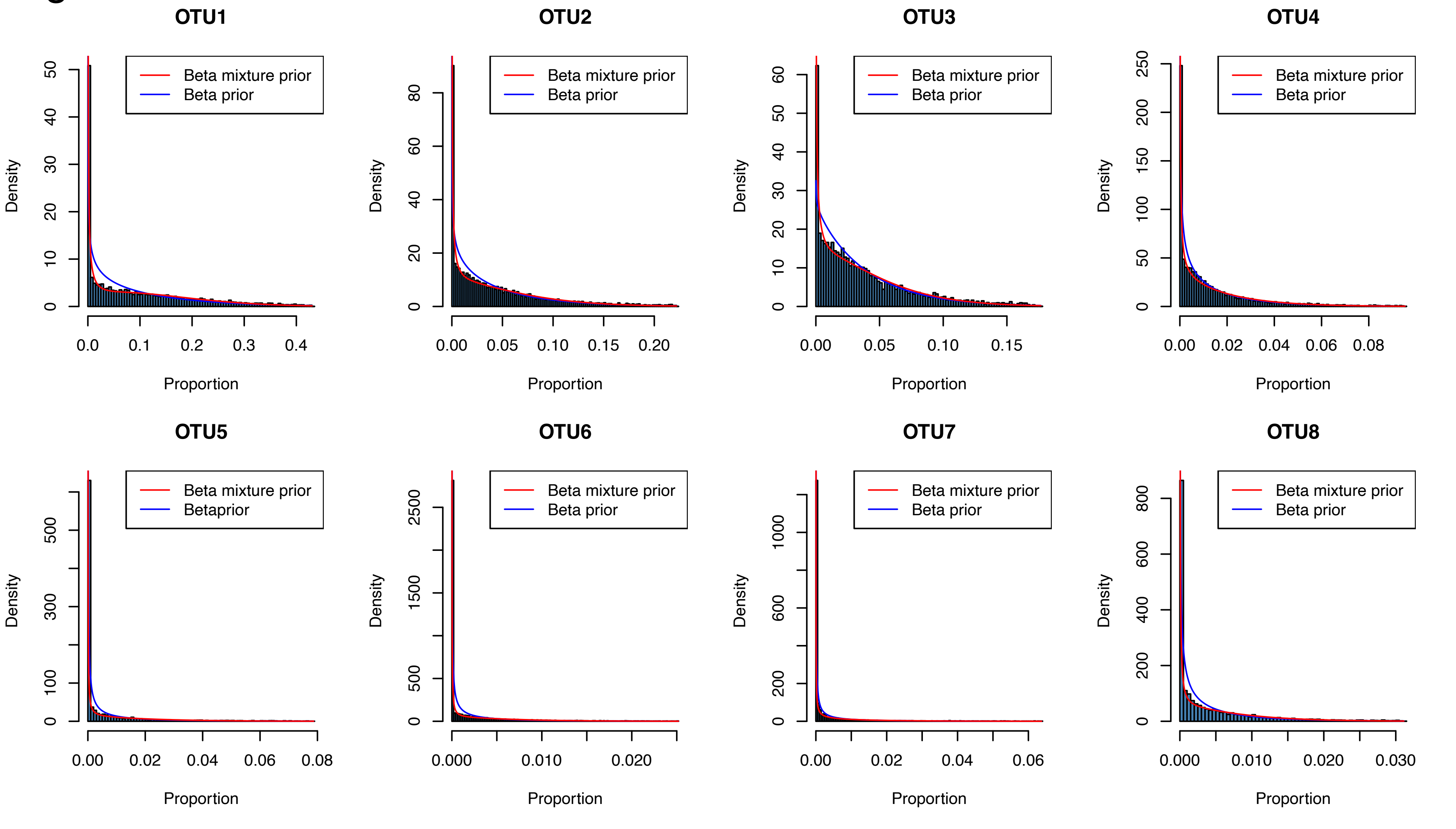
OTU1
OTU2
OTU3
OTU4
OTU5
OTU6
OTU7
OTU8
Beta mixture prior
Beta prior
Betaprior
Density
Proportion

Fig. S4

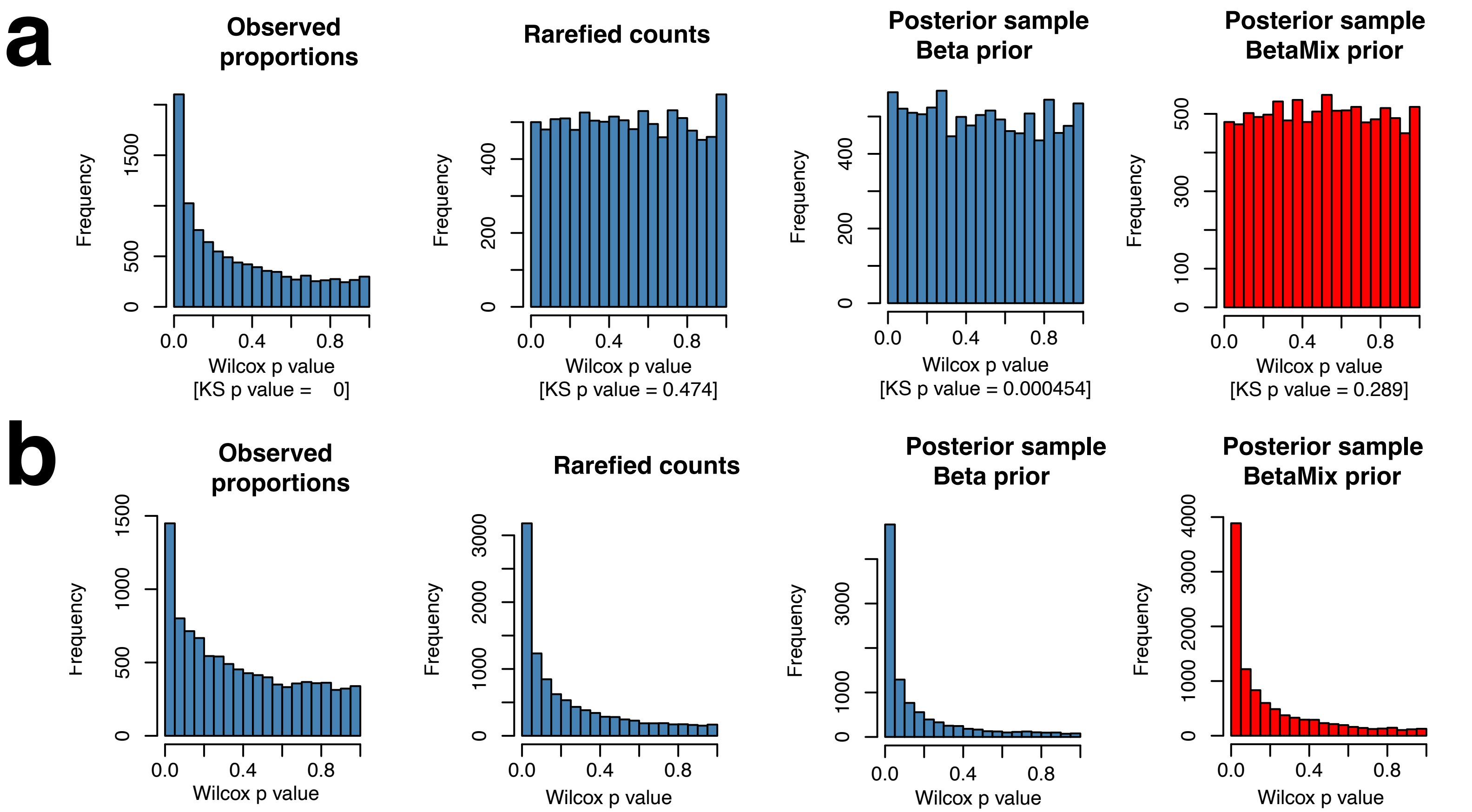

a
Observed proportions
Frequency
Wilcox p value
[KS p value = 0]
Rarefied counts
Frequency
Wilcox p value
[KS p value = 0.474]
Posterior sample Beta prior
Frequency
Wilcox p value
[KS p value = 0.000454]
Posterior sample BetaMix prior
Frequency
Wilcox p value
[KS p value = 0.289]
b
Observed proportions
Frequency
Wilcox p value
Rarefied counts
Frequency
Wilcox p value
Posterior sample Beta prior
Frequency
Wilcox p value
Posterior sample BetaMix prior
Frequency
Wilcox p value

Fig. S5

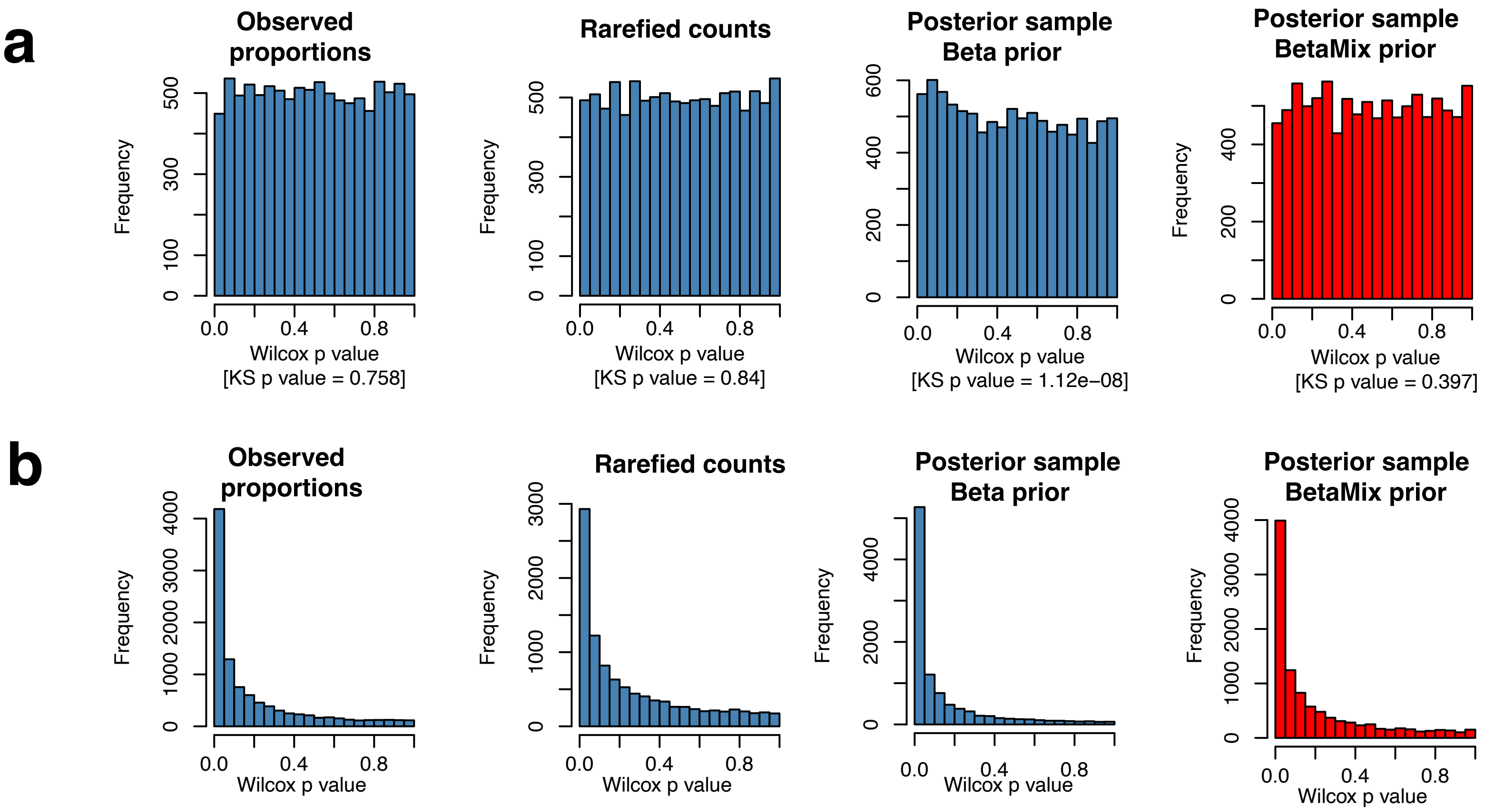
a
Observed proportions
Frequency
Wilcox p value
[KS p value = 0.758]
Rarefied counts
Frequency
Wilcox p value
[KS p value = 0.84]
Posterior sample Beta prior
Frequency
Wilcox p value
[KS p value = 1.12e−08]
Posterior sample BetaMix prior
Frequency
Wilcox p value
[KS p value = 0.397]
b
Observed proportions
Frequency
Wilcox p value
Rarefied counts
Frequency
Wilcox p value
Posterior sample Beta prior
Frequency
Wilcox p value
Posterior sample BetaMix prior
Frequency
Wilcox p value

Fig.S6

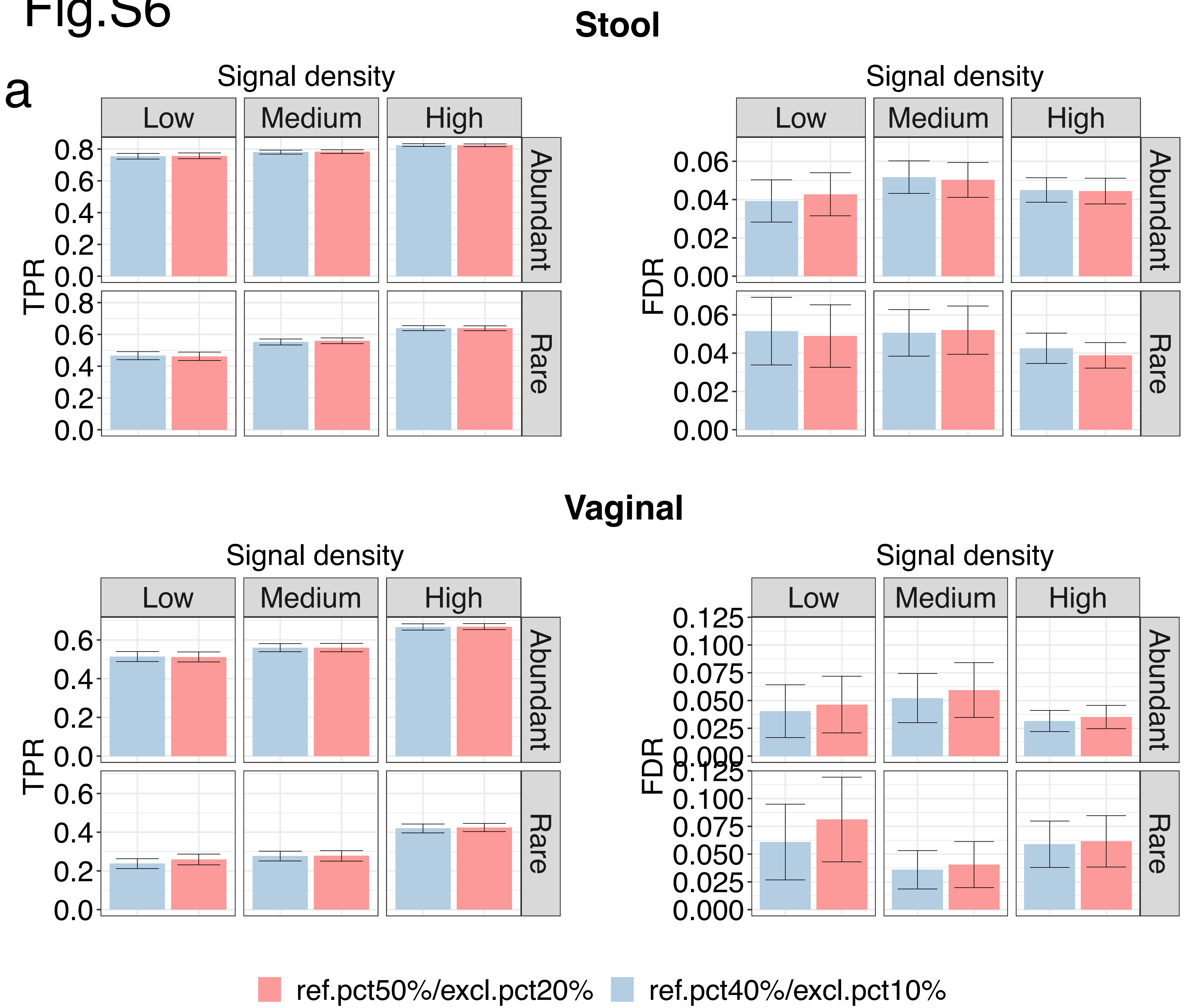

Stool
a
Signal density
Low
Medium
High
Abundant
Rare
TPR
0.0
0.2
0.4
0.6
0.8
FDR
0.00
0.02
0.04
0.06
Vaginal
Signal density
Low
Medium
High
Abundant
Rare
TPR
0.0
0.2
0.4
0.6
FDR
0.000
0.025
0.050
0.075
0.100
0.125
ref.pct50%/excl.pct20%
ref.pct40%/excl.pct10%

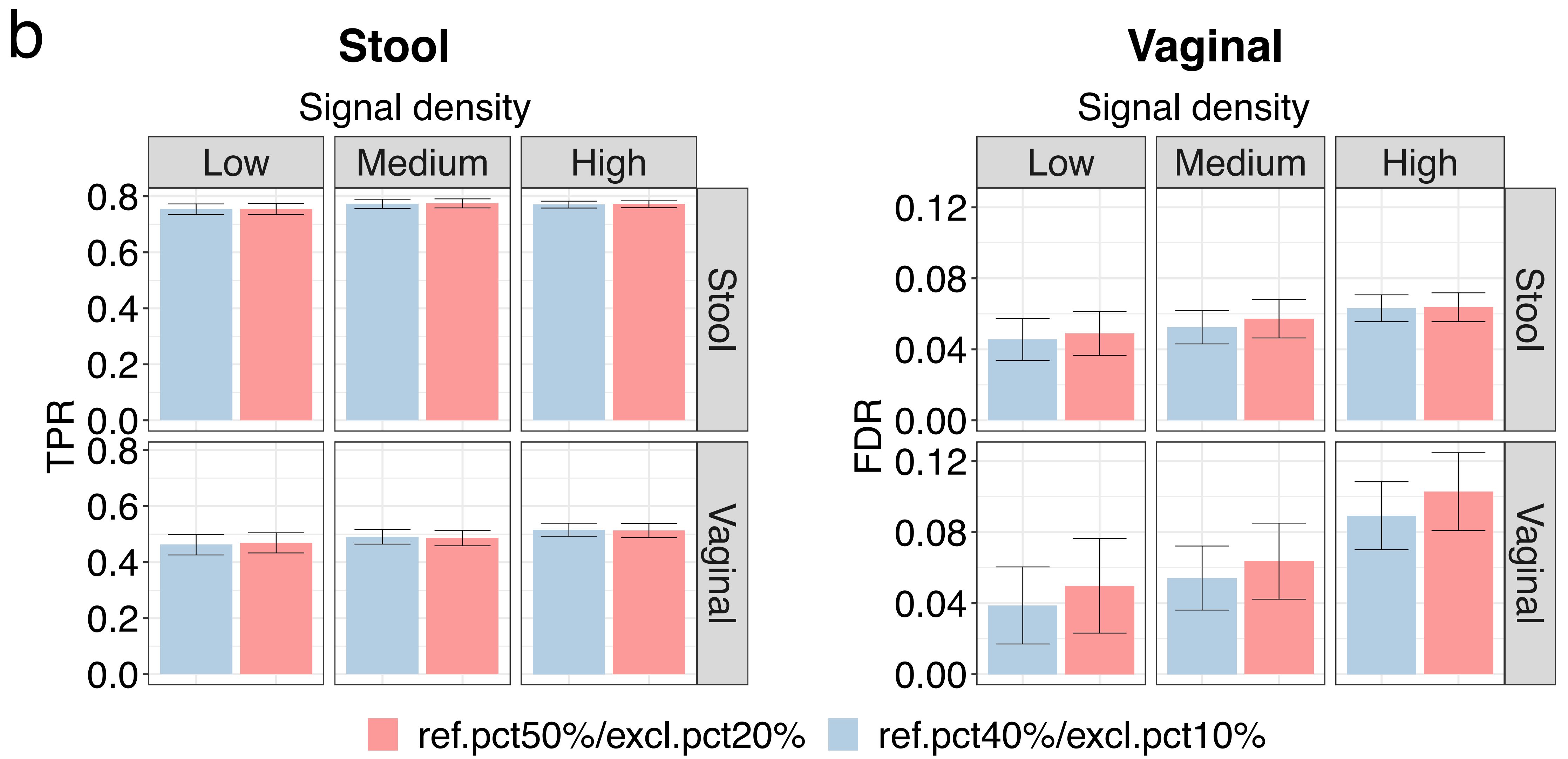
b
Stool
Vaginal
Signal density
Low
Medium
High
Stool
Vaginal
TPR
FDR
0.0
0.2
0.4
0.6
0.8
0.00
0.04
0.08
0.12
ref.pct50%/excl.pct20%
ref.pct40%/excl.pct10%

Fig. S7

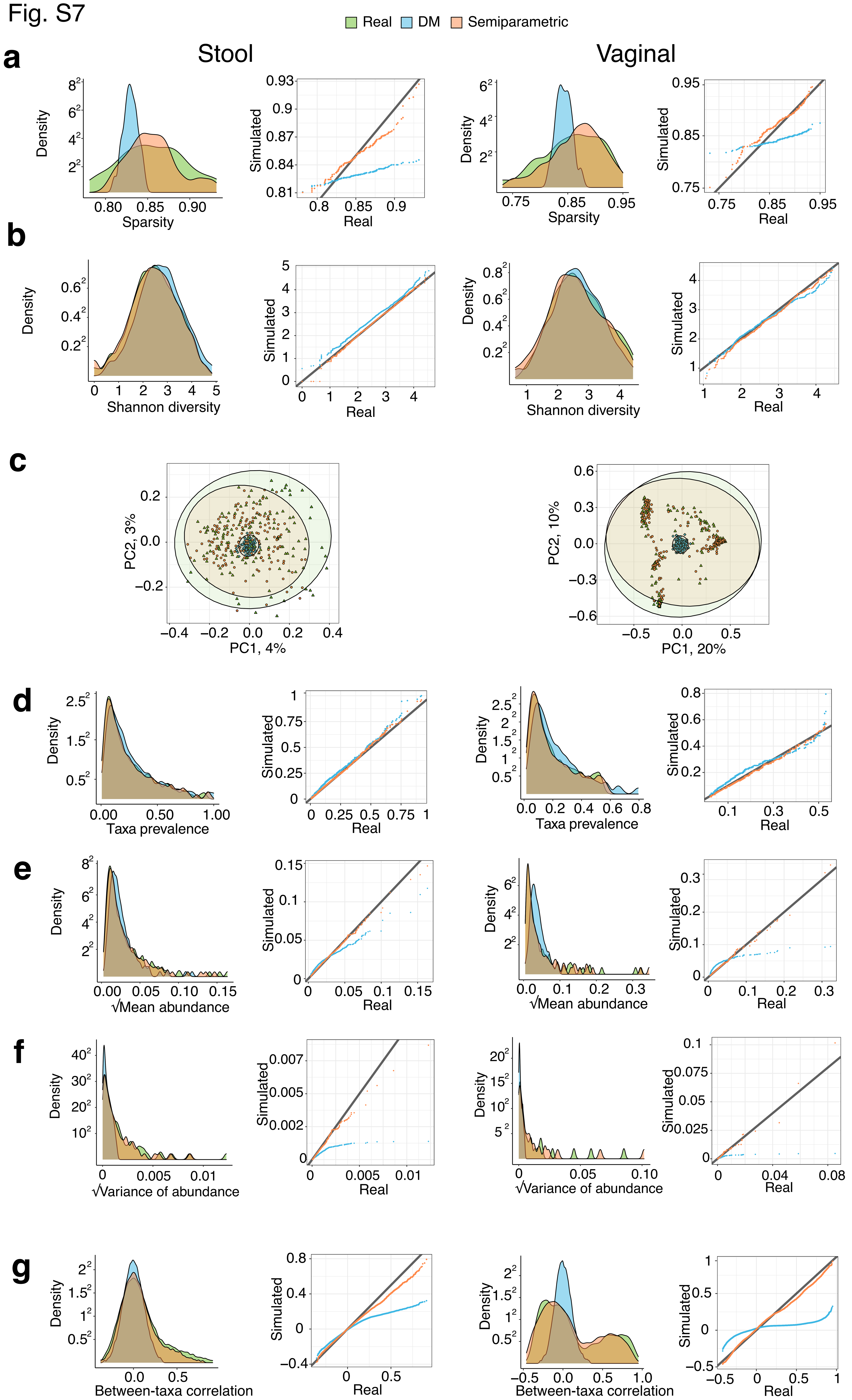

Real
DM
Semiparametric
a
Stool
Vaginal
Density
Sparsity
Simulated
Real
b
Shannon diversity
c
PC2, 3%
PC1, 4%
PC2, 10%
PC1, 20%
d
Taxa prevalence
e
√Mean abundance
f
√Variance of abundance
g
Between-taxa correlation

Fig. S8

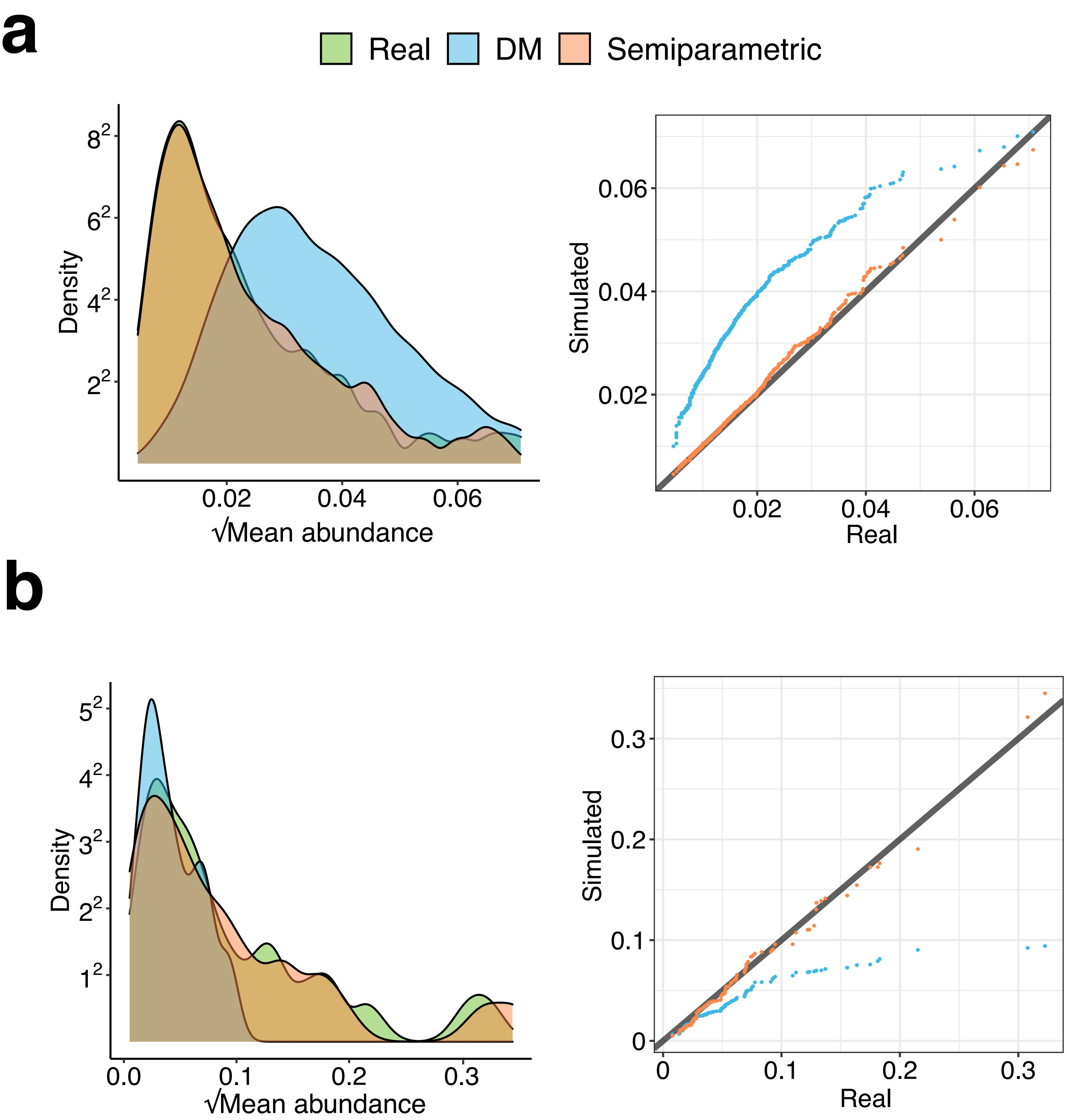

a
Real
DM
Semiparametric
Density
√Mean abundance
Simulated
Real
b
Density
√Mean abundance
Simulated
Real

Fig. S9

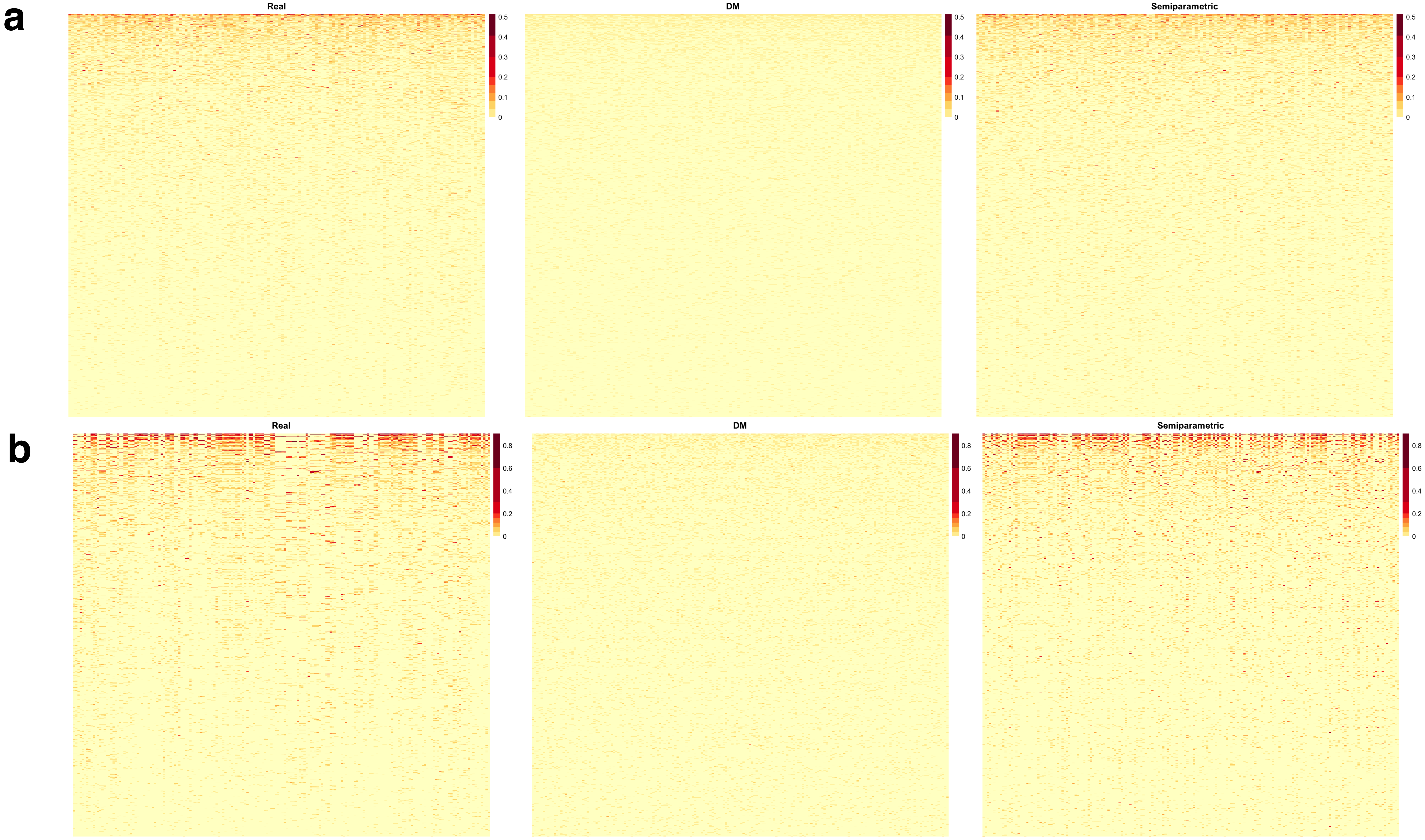

a
Real
DM
Semiparametric
0.5
0.4
0.3
0.2
0.1
0
b
Real
DM
Semiparametric
0.8
0.6
0.4
0.2
0

Fig.S10

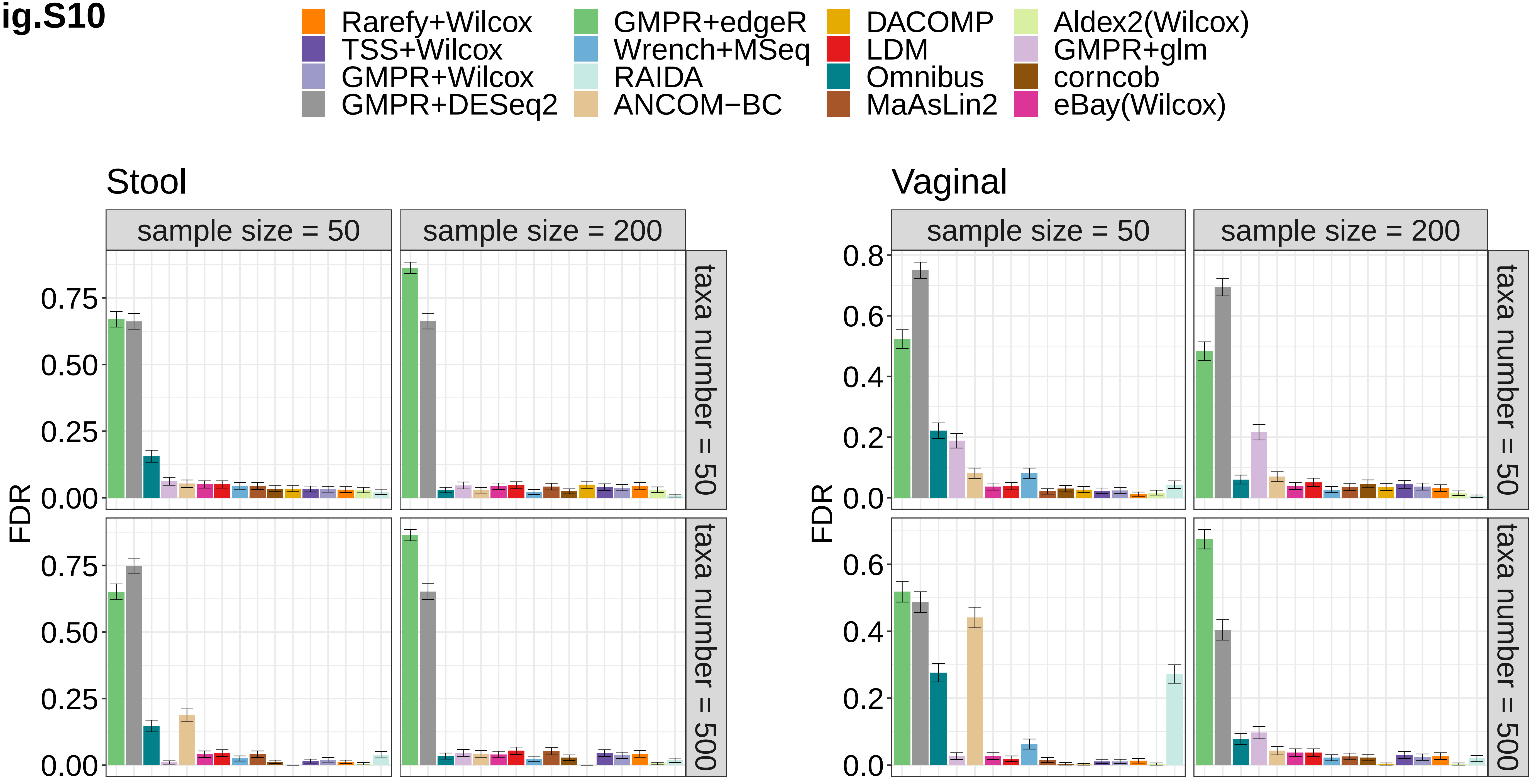

Rarefy+Wilcox
TSS+Wilcox
GMPR+Wilcox
GMPR+DESeq2
GMPR+edgeR
Wrench+MSeq
RAIDA
ANCOM−BC
DACOMP
LDM
Omnibus
MaAsLin2
Aldex2(Wilcox)
GMPR+glm
corncob
eBay(Wilcox)
Stool
Vaginal
sample size = 50
sample size = 200
taxa number = 50
taxa number = 500
FDR
0.00
0.25
0.50
0.75
0.0
0.2
0.4
0.6
0.8

Fig. S11

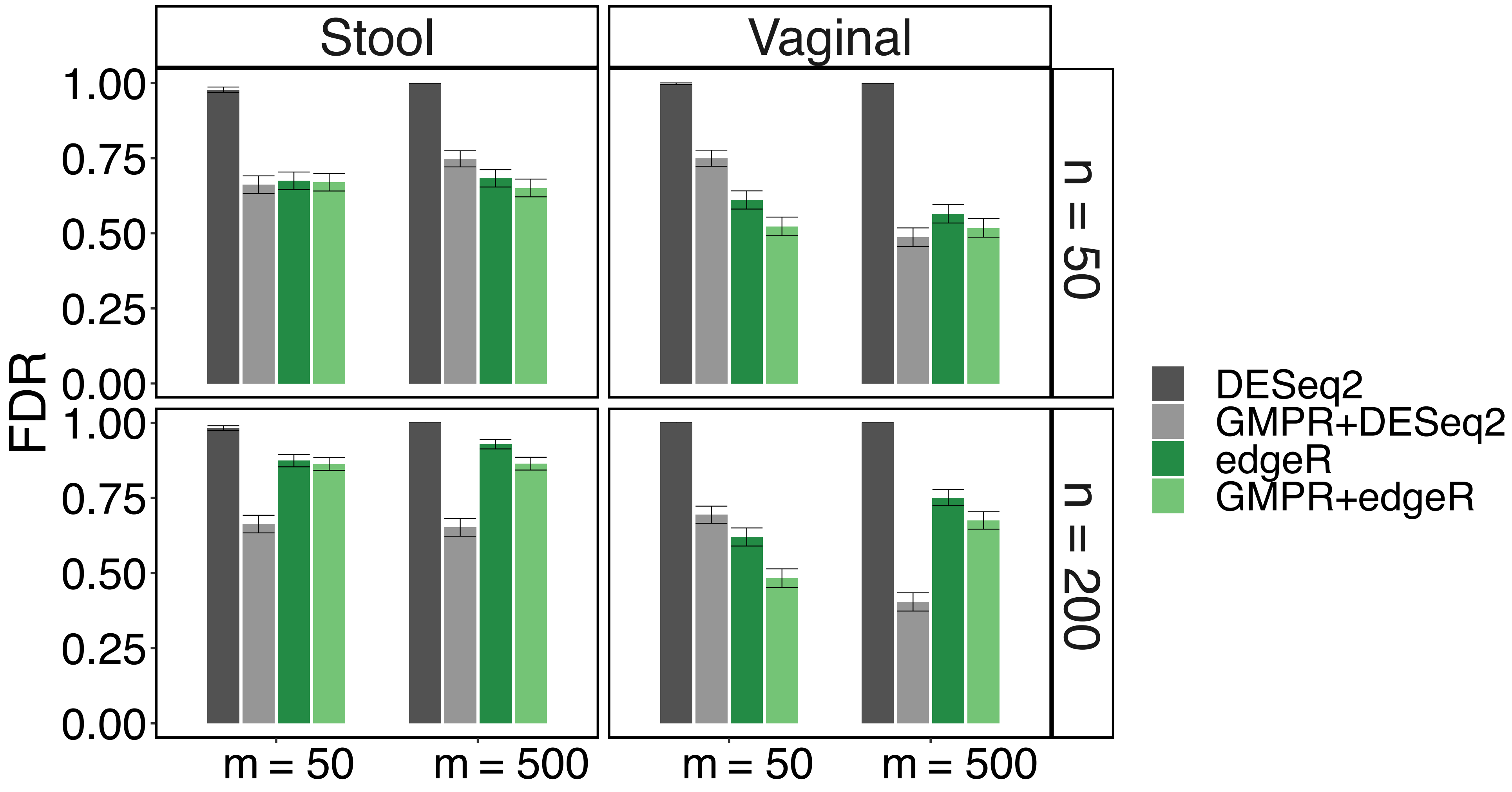

Stool
Vaginal
1.00
0.75
0.50
0.25
0.00
FDR
n = 50
n = 200
m = 50
m = 500
DESeq2
GMPR+DESeq2
edgeR
GMPR+edgeR

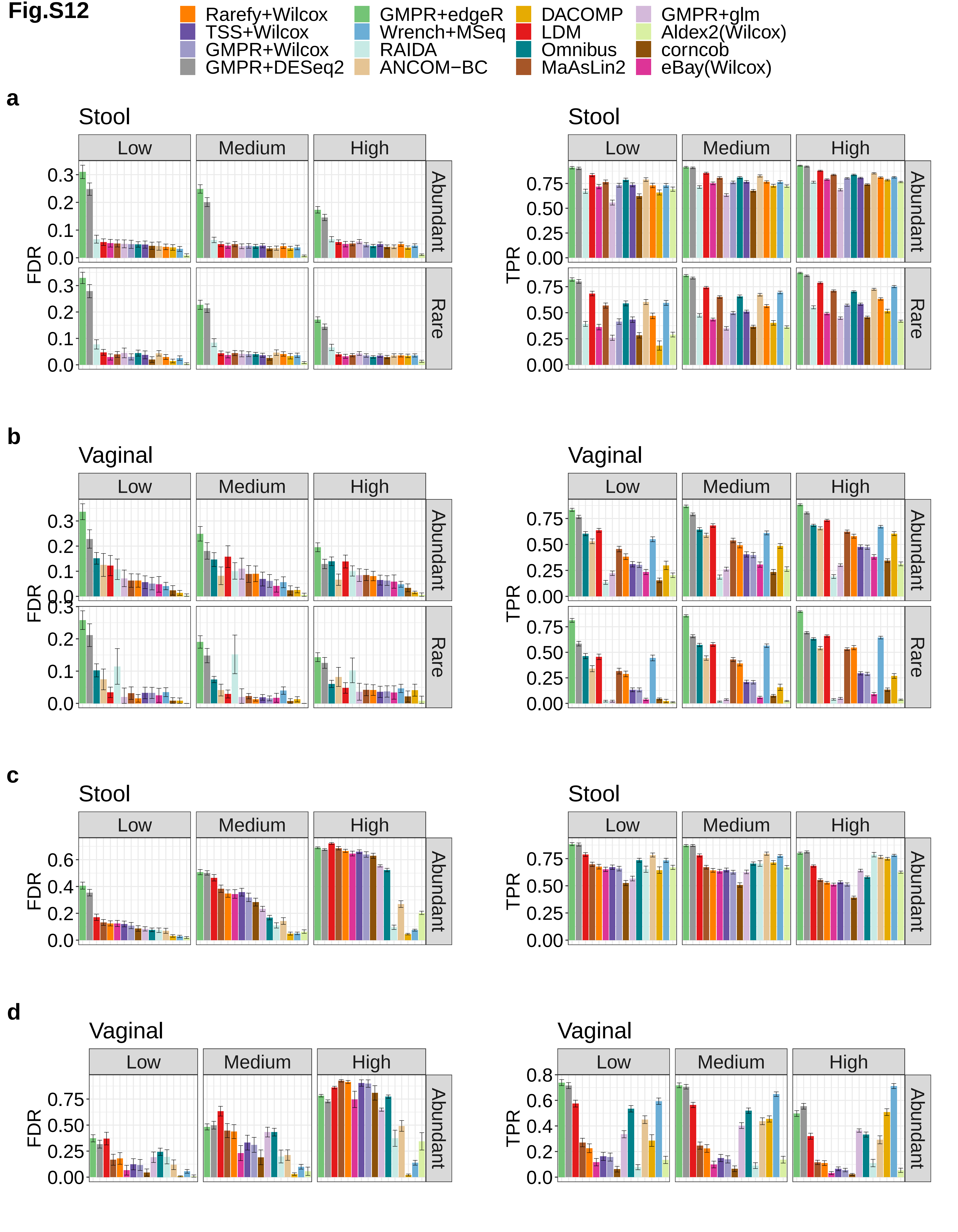
Fig.S12
Rarefy+Wilcox
TSS+Wilcox
GMPR+Wilcox
GMPR+DESeq2
GMPR+edgeR
Wrench+MSeq
RAIDA
ANCOM−BC
DACOMP
LDM
Omnibus
MaAsLin2
GMPR+glm
Aldex2(Wilcox)
corncob
eBay(Wilcox)
a
Stool
Low
Medium
High
Abundant
Rare
FDR
TPR
b
Vaginal
c
Stool
d
Vaginal

Fig. S13

a

Signal density	Abundant Low	Abundant Medium	Abundant High	Rare Low	Rare Medium	Rare High	Rank FDR	Rank TPR
LDM	0.56	0.62	0.69	0.25	0.34	0.43	12	14
Wrench+MSeq	0.42	0.48	0.56	0.17	0.25	0.35	12	10
Rarefy+Wilcox	0.43	0.48	0.56	0.09	0.15	0.23	12	8
TSS+Wilcox	0.43	0.48	0.56	0.08	0.12	0.21	12	7
GMPR+Wilcox	0.41	0.47	0.54	0.08	0.11	0.19	12	6
MaAsLin2	0.48	0.53	0.61	0.17	0.24	0.32	6.5	11
ANCOM-BC	0.56	0.60	0.67	0.24	0.31	0.38	4.5	13
Aldex2(Wilcox)	0.42	0.47	0.53	0.05	0.07	0.12	12	5
GMPR+edgeR	0.74	0.77	0.81	0.52	0.59	0.65	1.5	15.5
GMPR+DESeq2	0.75	0.78	0.80	0.55	0.58	0.63	1.5	15.5
eBay(Wilcox)	0.45	0.50	0.57	0.09	0.11	0.19	6.5	9
corncob	0.28	0.33	0.42	0.05	0.05	0.09	12	3
Omnibus	0.53	0.58	0.63	0.23	0.31	0.38	3	12
DACOMP	0.14	0.37	0.49	0.00	0.02	0.09	12	2
GMPR+glm	0.20	0.25	0.32	0.03	0.04	0.08	12	1
RAIDA	0.39	0.46	0.51	0.06	0.10	0.16	4.5	4

b

Signal density	Abundant Low	Abundant Medium	Abundant High	Rare Low	Rare Medium	Rare High	Rank FDR	Rank TPR
LDM	0.28	0.37	0.46	0.06	0.09	0.13	11	12
Wrench+MSeq	0.28	0.34	0.45	0.07	0.09	0.14	11	12
MaAsLin2	0.11	0.18	0.27	0.03	0.05	0.06	11	10
GMPR+DESeq2	0.57	0.62	0.64	0.30	0.33	0.40	4.5	16
Rarefy+Wilcox	0.07	0.13	0.19	0.03	0.02	0.03	11	9
eBay(Wilcox)	0.05	0.08	0.10	0.02	0.01	0.02	11	8
TSS+Wilcox	0.04	0.08	0.12	0.01	0.01	0.02	11	7
GMPR+Wilcox	0.05	0.08	0.11	0.01	0.01	0.02	11	6
Omnibus	0.35	0.42	0.49	0.13	0.18	0.23	3	14
ANCOM-BC	0.21	0.28	0.36	0.09	0.12	0.15	4.5	12
GMPR+edgeR	0.31	0.44	0.59	0.28	0.37	0.50	1.5	15
DACOMP	0.02	0.09	0.18	0.00	0.00	0.00	11	4
GMPR+glm	0.05	0.07	0.10	0.00	0.00	0.00	11	3
Aldex2(Wilcox)	0.04	0.06	0.07	0.00	0.00	0.00	11	2
corncob	0.02	0.03	0.04	0.00	0.00	0.00	11	1
RAIDA	0.03	0.06	0.08	0.03	0.02	0.04	1.5	5

c

Signal density	Stool Low	Stool Medium	Stool High	Rank FDR	Rank TPR
Wrench+MSeq	0.42	0.46	0.43	15.5	13
Aldex2(Wilcox)	0.37	0.35	0.26	14	10
ANCOM-BC	0.51	0.51	0.46	9.5	14
DACOMP	0.15	0.32	0.36	15.5	7
RAIDA	0.28	0.34	0.39	9.5	9
GMPR+DESeq2	0.72	0.69	0.54	1.5	16
GMPR+glm	0.24	0.26	0.22	13	4
Omnibus	0.45	0.40	0.24	6	11
GMPR+edgeR	0.69	0.64	0.49	1.5	15
LDM	0.48	0.43	0.28	4	12
TSS+Wilcox	0.33	0.26	0.15	9.5	5
Rarefy+Wilcox	0.33	0.26	0.14	9.5	3
MaAsLin2	0.37	0.30	0.18	4	8
GMPR+Wilcox	0.29	0.24	0.11	9.5	2
corncob	0.18	0.14	0.07	9.5	1
eBay(Wilcox)	0.35	0.29	0.16	4	6

d

Signal density	Vaginal Low	Vaginal Medium	Vaginal High	Rank FDR	Rank TPR
Wrench+MSeq	0.31	0.38	0.40	14	15
DACOMP	0.01	0.06	0.09	15.5	8
GMPR+glm	0.11	0.17	0.11	8.5	12
Aldex2(Wilcox)	0.02	0.02	0.01	15.5	4
GMPR+DESeq2	0.50	0.41	0.20	3	16
MaAsLin2	0.05	0.03	0.01	8.5	9
LDM	0.20	0.14	0.06	6	11
Omnibus	0.31	0.25	0.12	3	14
GMPR+edgeR	0.22	0.14	0.09	3	13
Rarefy+Wilcox	0.03	0.02	0.01	8.5	7
TSS+Wilcox	0.03	0.01	0.01	11.5	3
corncob	0.01	0.01	0.00	13	1
GMPR+Wilcox	0.03	0.01	0.01	11.5	2
eBay(Wilcox)	0.03	0.01	0.01	8.5	5
ANCOM-BC	0.15	0.13	0.07	3	10
RAIDA	0.02	0.02	0.02	3	6

Fig. S14

a

	Abundant			Rare			Rank	
Signal density	Low	Medium	High	Low	Medium	High	FDR	TPR
ANCOM-BC	0.92	0.91	0.91	0.72	0.80	0.80	15	12
Omnibus	0.92	0.91	0.91	0.76	0.82	0.81	12.5	13
Aldex2(Wilcox)	0.92	0.89	0.89	0.72	0.76	0.78	15	7
LDM	0.96	0.93	0.93	0.84	0.88	0.88	4.5	14
GMPR+DESeq2	0.97	0.96	0.95	0.91	0.93	0.93	1.5	16
RAIDA	0.82	0.81	0.85	0.44	0.58	0.66	15	2
corncob	0.86	0.86	0.87	0.54	0.68	0.68	12.5	4
TSS+Wilcox	0.92	0.89	0.91	0.70	0.79	0.79	7.5	9
GMPR+edgeR	0.94	0.95	0.94	0.91	0.93	0.90	1.5	15
DACOMP	0.89	0.88	0.86	0.62	0.73	0.74	10.5	5
MaAsLin2	0.91	0.90	0.91	0.72	0.81	0.81	4.5	11
GMPR+Wilcox	0.92	0.89	0.90	0.70	0.79	0.78	7.5	8
Rarefy+Wilcox	0.92	0.89	0.90	0.72	0.79	0.80	4.5	10
Wrench+MSeq	0.30	0.28	0.33	0.62	0.71	0.75	9	3
GMPR+glm	0.74	0.75	0.77	0.43	0.52	0.61	10.5	1
eBay(Wilcox)	0.92	0.89	0.90	0.70	0.78	0.79	4.5	6

b

	Abundant			Rare			Rank	
Signal density	Low	Medium	High	Low	Medium	High	FDR	TPR
Aldex2(Wilcox)	0.51	0.63	0.69	0.27	0.30	0.32	14	9
GMPR+Wilcox	0.42	0.61	0.67	0.28	0.36	0.38	14	7
MaAsLin2	0.44	0.61	0.67	0.38	0.50	0.51	10.5	10
ANCOM-BC	0.62	0.73	0.76	0.35	0.46	0.48	8	12
LDM	0.76	0.83	0.85	0.50	0.61	0.62	4.5	15
eBay(Wilcox)	0.45	0.60	0.65	0.23	0.31	0.31	14	5
TSS+Wilcox	0.44	0.60	0.68	0.28	0.38	0.38	10.5	8
Omnibus	0.65	0.74	0.77	0.38	0.50	0.50	4.5	13
DACOMP	0.49	0.57	0.64	0.12	0.11	0.12	14	3
Wrench+MSeq	0.62	0.69	0.70	0.34	0.45	0.47	6	11
GMPR+DESeq2	0.78	0.81	0.83	0.64	0.75	0.75	1	16
corncob	0.36	0.46	0.53	0.17	0.22	0.26	14	2
GMPR+edgeR	0.68	0.75	0.78	0.63	0.73	0.76	2	14
Rarefy+Wilcox	0.41	0.56	0.64	0.28	0.44	0.41	8	6
RAIDA	0.02	0.11	0.14	0.03	0.06	0.08	8	1
GMPR+glm	0.56	0.57	0.61	0.22	0.23	0.20	3	4

c

	Stool			Rank	
Signal density	Low	Medium	High	FDR	TPR
ANCOM-BC	0.87	0.90	0.92	15	14
Aldex2(Wilcox)	0.86	0.87	0.88	11.5	12
Omnibus	0.86	0.86	0.83	11.5	10
RAIDA	0.72	0.81	0.83	16	4
DACOMP	0.85	0.84	0.70	14	5
eBay(Wilcox)	0.86	0.86	0.84	6.5	11
GMPR+DESeq2	0.92	0.94	0.92	1.5	16
GMPR+edgeR	0.92	0.93	0.91	1.5	15
LDM	0.88	0.90	0.88	3	13
TSS+Wilcox	0.86	0.86	0.85	6.5	8.5
MaAsLin2	0.86	0.86	0.84	6.5	8.5
GMPR+glm	0.66	0.73	0.75	11.5	2
Rarefy+Wilcox	0.85	0.86	0.84	6.5	7
Wrench+MSeq	0.30	0.24	0.22	11.5	1
GMPR+Wilcox	0.86	0.85	0.84	6.5	6
corncob	0.82	0.81	0.74	6.5	3

d

	Vaginal			Rank	
Signal density	Low	Medium	High	FDR	TPR
Wrench+MSeq	0.59	0.71	0.72	14.5	13
DACOMP	0.43	0.53	0.53	16	8
Aldex2(Wilcox)	0.50	0.54	0.42	13	9
ANCOM-BC	0.62	0.65	0.56	11.5	10
GMPR+DESeq2	0.77	0.81	0.82	2	16
Omnibus	0.64	0.70	0.64	4.5	12
GMPR+edgeR	0.69	0.76	0.76	2	14.5
LDM	0.74	0.80	0.72	2	14.5
RAIDA	0.04	0.04	0.09	14.5	1
GMPR+glm	0.51	0.66	0.68	4.5	11
TSS+Wilcox	0.46	0.48	0.28	8	7
GMPR+Wilcox	0.47	0.46	0.24	8	6
corncob	0.30	0.26	0.11	11.5	2
eBay(Wilcox)	0.46	0.45	0.27	8	5
Rarefy+Wilcox	0.41	0.43	0.25	8	4
MaAsLin2	0.42	0.33	0.14	8	3

Fig. S15

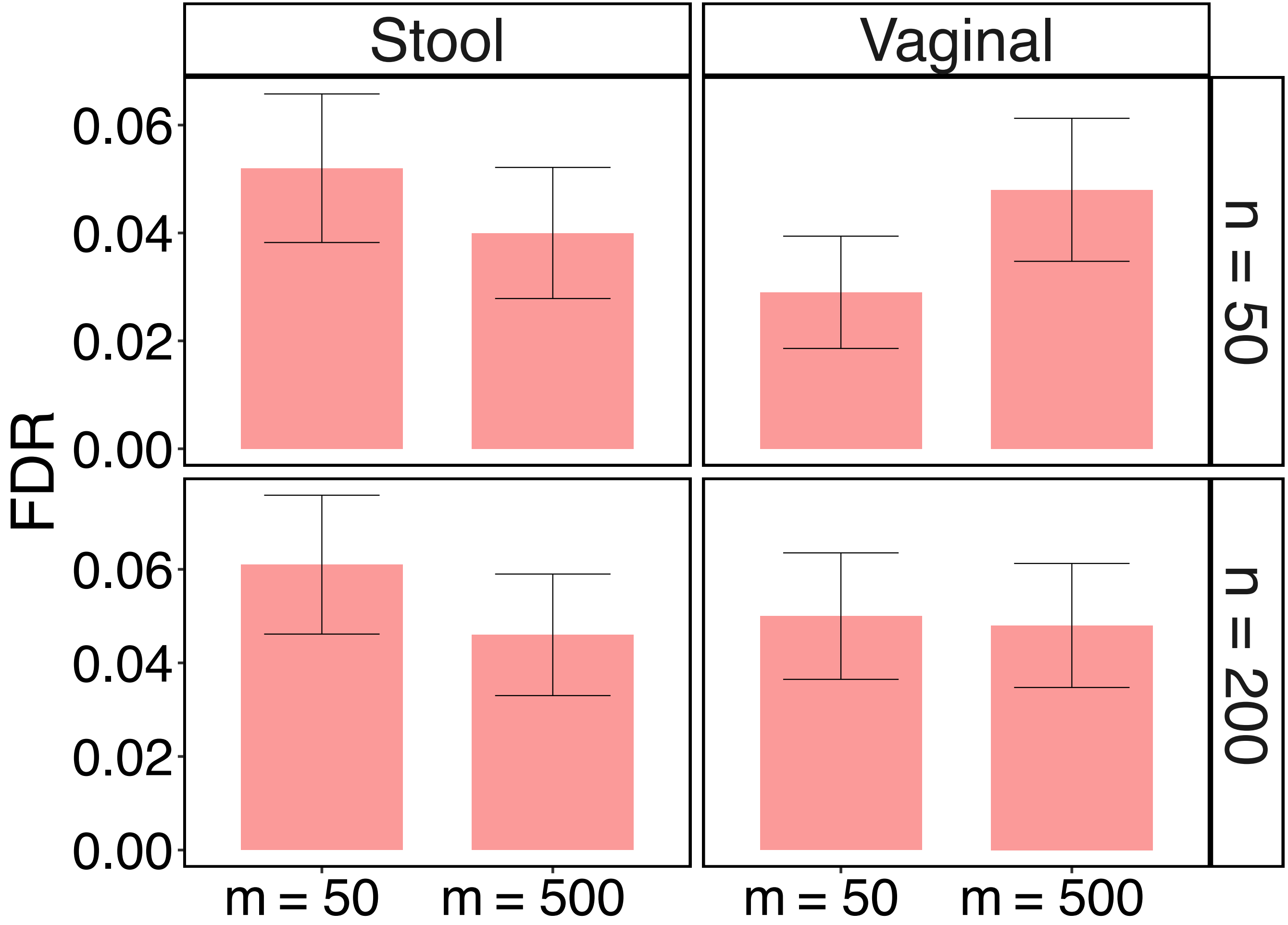

Stool
Vaginal
n = 50
n = 200
FDR
0.06
0.04
0.02
0.00
0.06
0.04
0.02
0.00
m = 50
m = 500
m = 50
m = 500

Fig.S16

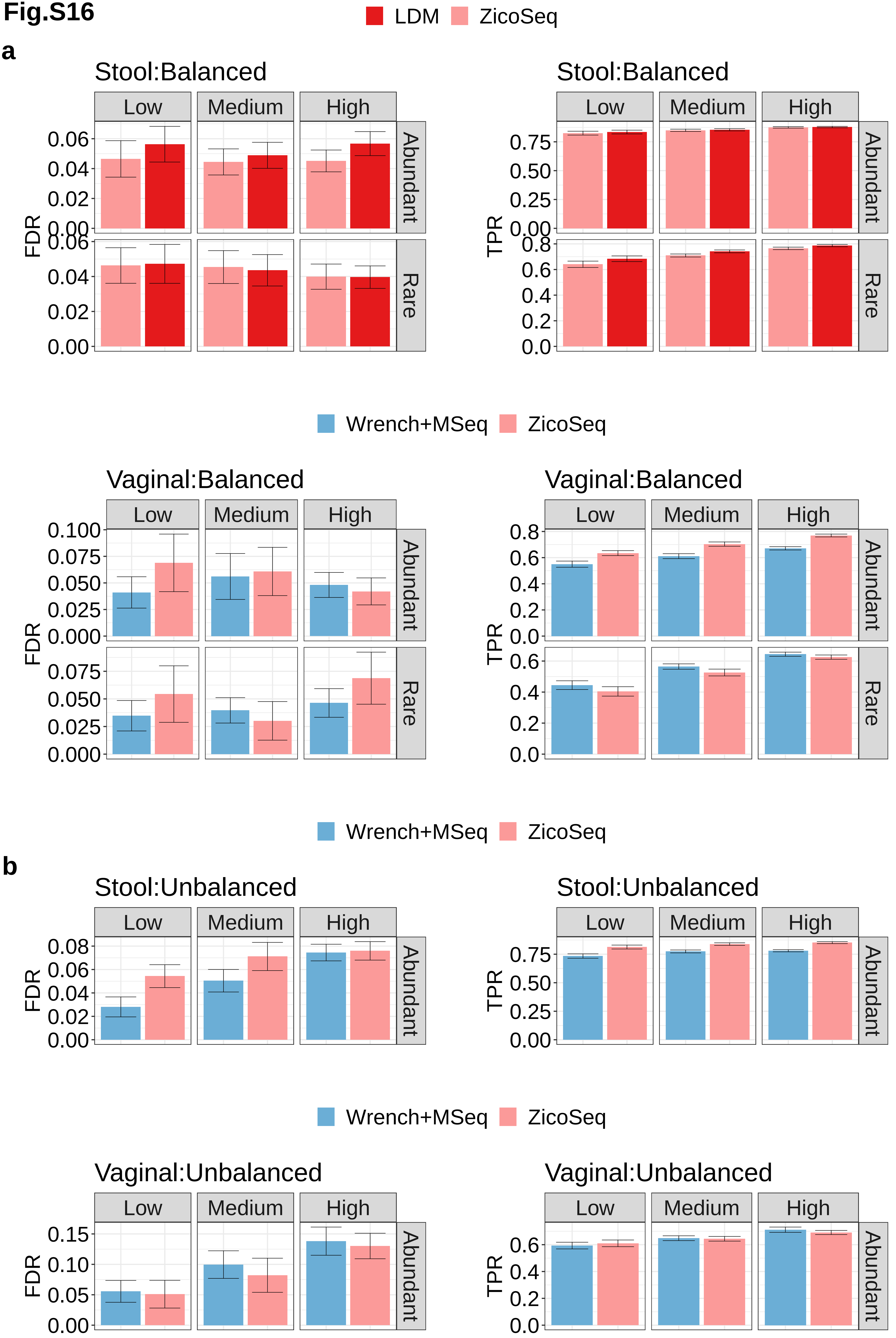
LDM
ZicoSeq
a
Stool:Balanced
Low
Medium
High
Abundant
Rare
FDR
0.06
0.04
0.02
0.00
TPR
0.75
0.50
0.25
0.8
0.6
0.4
0.2
0.0
Wrench+MSeq
ZicoSeq
Vaginal:Balanced
0.100
0.075
0.050
0.025
0.000
b
Stool:Unbalanced
0.08
Vaginal:Unbalanced
0.15
0.10
0.05

Fig.S16

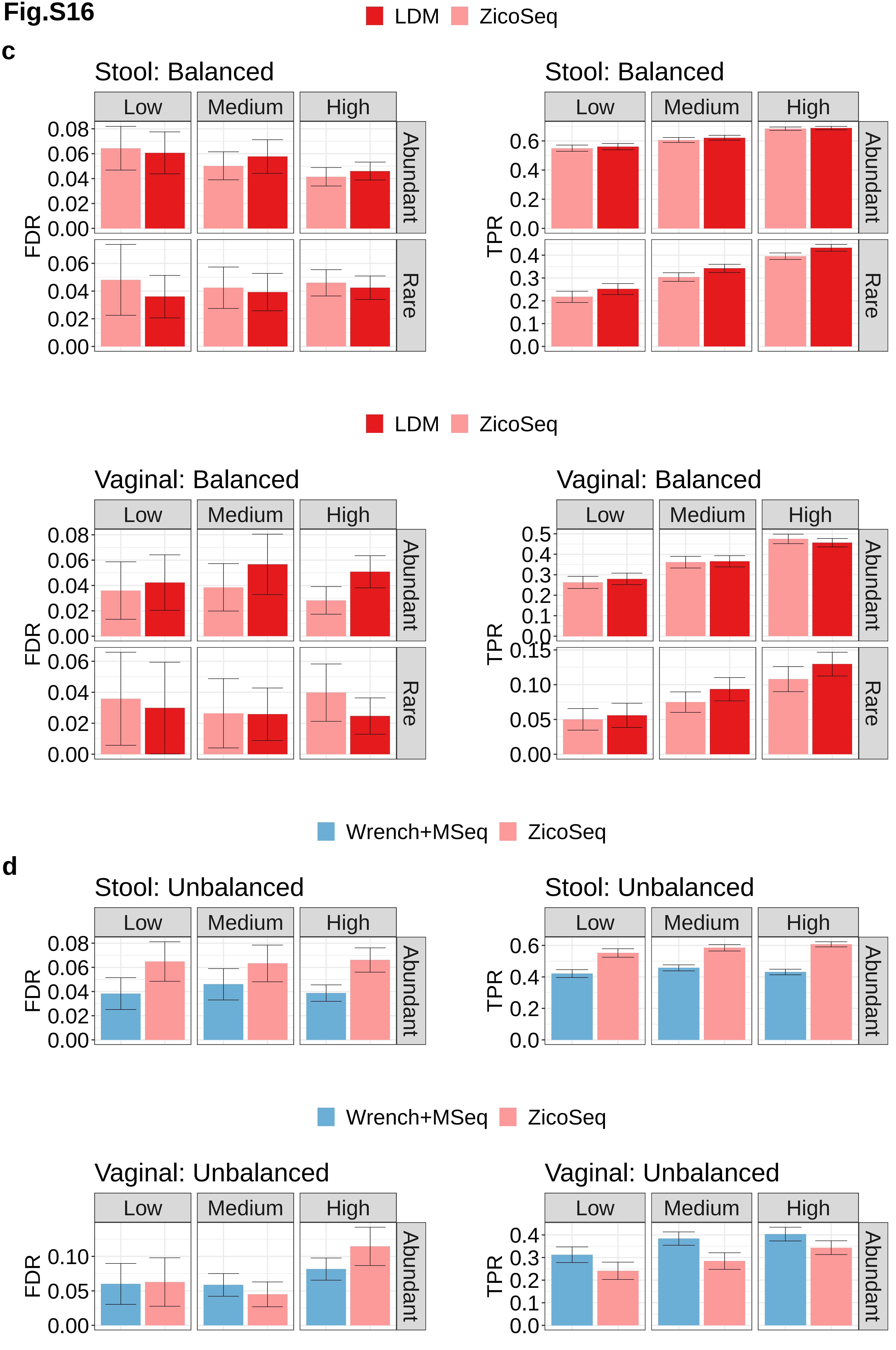
LDM
ZicoSeq
c
Stool: Balanced
Low
Medium
High
Abundant
Rare
FDR
TPR
Vaginal: Balanced
Wrench+MSeq
d
Stool: Unbalanced
Vaginal: Unbalanced
0.00
0.02
0.04
0.06
0.08
0.0
0.1
0.2
0.3
0.4
0.5
0.6
0.05
0.10
0.15

Fig.S16

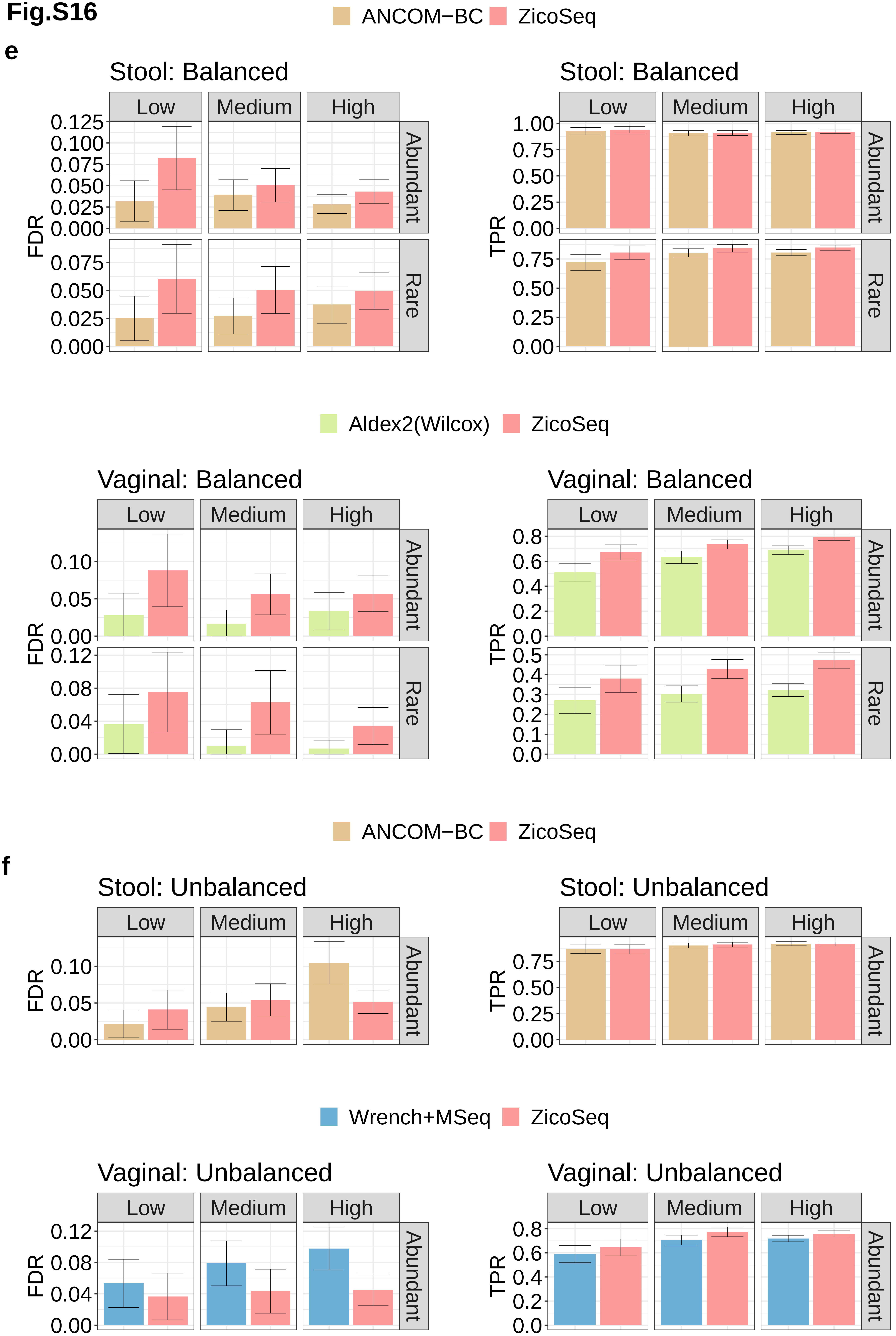

ANCOM−BC
ZicoSeq
e
Stool: Balanced
Low
Medium
High
Abundant
Rare
FDR
0.125
0.100
0.075
0.050
0.025
0.000
TPR
1.00
0.75
0.50
0.25
0.00
Aldex2(Wilcox)
ZicoSeq
Vaginal: Balanced
0.10
0.05
0.00
0.12
0.08
0.04
0.8
0.6
0.4
0.2
0.0
0.5
0.3
0.1
ANCOM−BC
ZicoSeq
f
Stool: Unbalanced
Wrench+MSeq
ZicoSeq
Vaginal: Unbalanced

Fig.S17

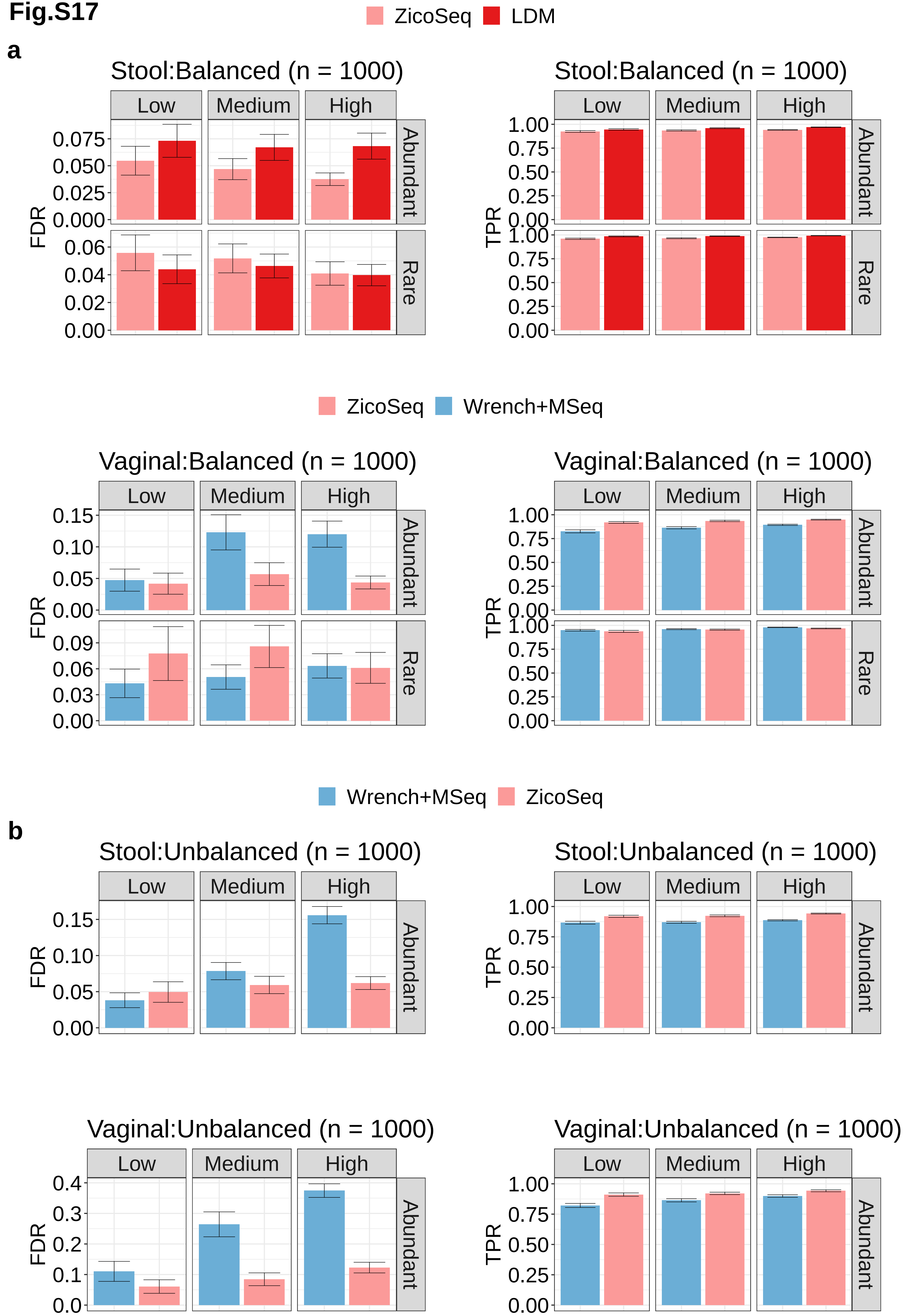

ZicoSeq
LDM
a
Stool:Balanced (n = 1000)
Stool:Balanced (n = 1000)
Low
Medium
High
Abundant
Rare
FDR
TPR
0.000
0.025
0.050
0.075
0.00
0.02
0.04
0.06
0.00
0.25
0.50
0.75
1.00
ZicoSeq
Wrench+MSeq
Vaginal:Balanced (n = 1000)
Vaginal:Balanced (n = 1000)
0.00
0.05
0.10
0.15
0.00
0.03
0.06
0.09
Wrench+MSeq
ZicoSeq
b
Stool:Unbalanced (n = 1000)
Stool:Unbalanced (n = 1000)
Vaginal:Unbalanced (n = 1000)
Vaginal:Unbalanced (n = 1000)
0.0
0.1
0.2
0.3
0.4

Fig.S18

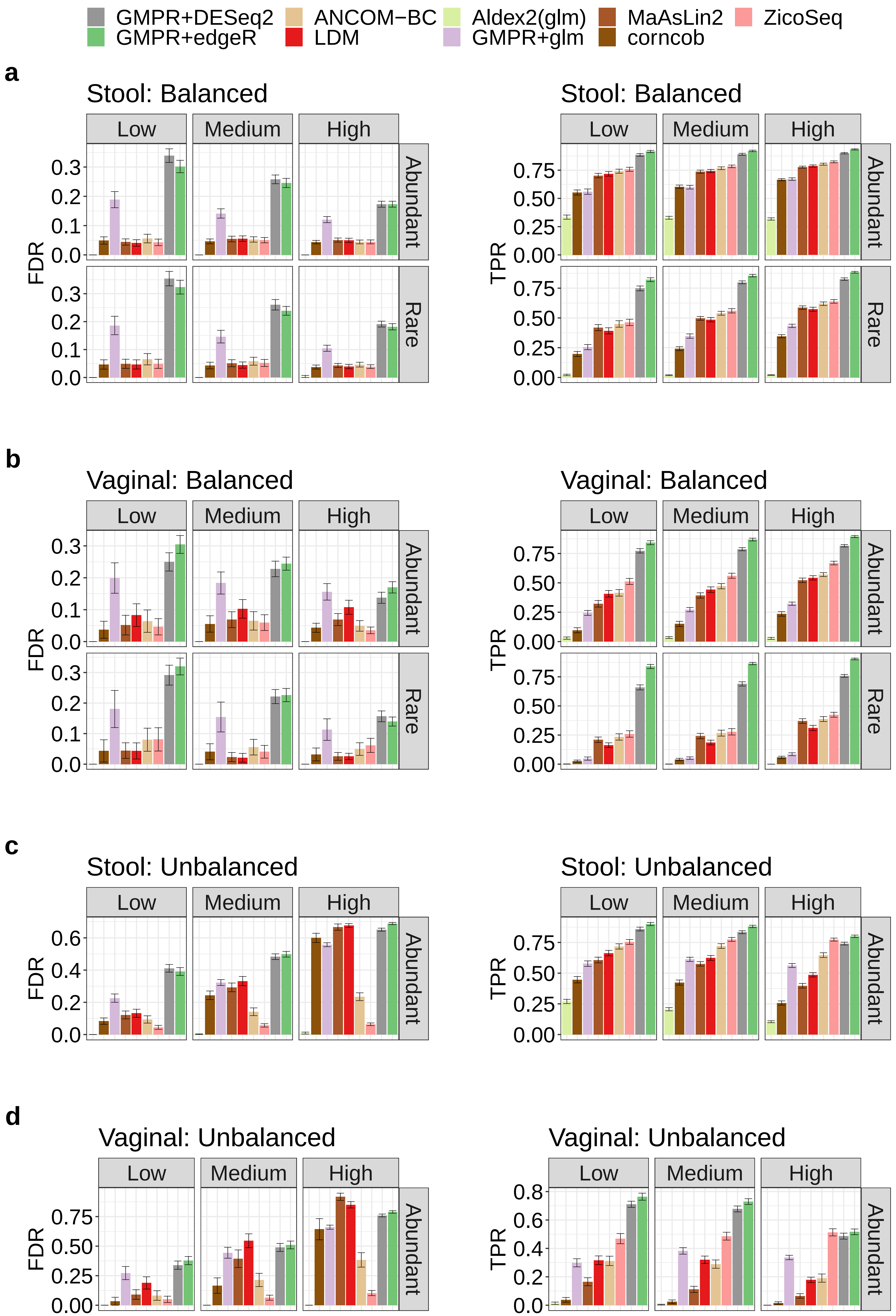

GMPR+DESeq2
GMPR+edgeR
ANCOM−BC
LDM
Aldex2(glm)
GMPR+glm
MaAsLin2
corncob
ZicoSeq
a
Stool: Balanced
b
Vaginal: Balanced
c
Stool: Unbalanced
d
Vaginal: Unbalanced
Low
Medium
High
Abundant
Rare
FDR
TPR

Fig.S19

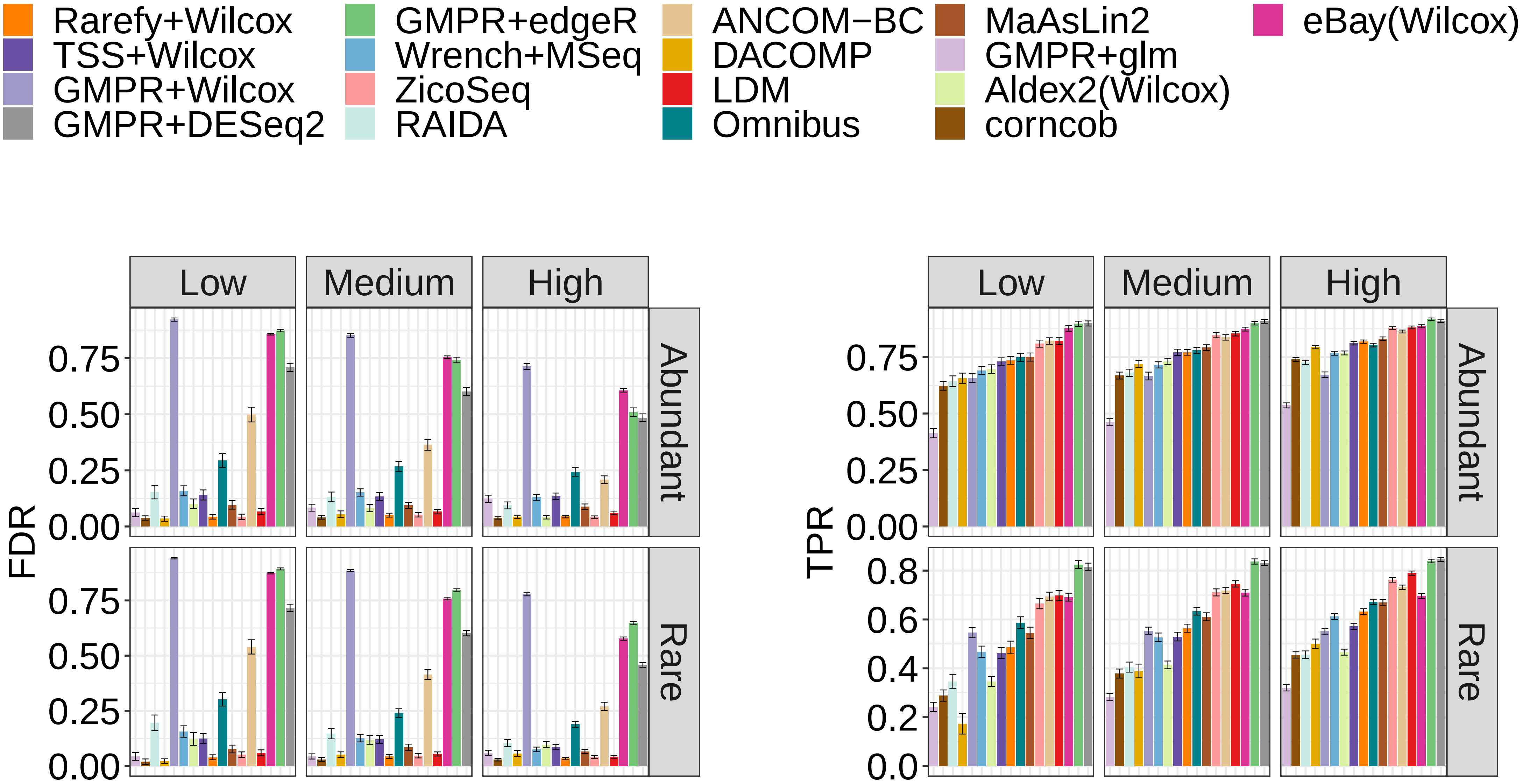

Rarefy+Wilcox
TSS+Wilcox
GMPR+Wilcox
GMPR+DESeq2
GMPR+edgeR
Wrench+MSeq
ZicoSeq
RAIDA
ANCOM−BC
DACOMP
LDM
Omnibus
MaAsLin2
GMPR+glm
Aldex2(Wilcox)
corncob
eBay(Wilcox)
Low
Medium
High
Abundant
Rare
FDR
TPR
0.00
0.25
0.50
0.75
0.0
0.2
0.4
0.6
0.8

Fig. S20

	Abundant			Rare			Rank	
Signal density	Low	Medium	High	Low	Medium	High	FDR	TPR
ZicoSeq	0.82	0.85	0.88	0.64	0.70	0.75	16	12
LDM	0.83	0.86	0.88	0.70	0.74	0.79	12	16
Rarefy+Wilcox	0.72	0.77	0.82	0.46	0.54	0.64	16	9
MaAsLin2	0.73	0.78	0.81	0.49	0.56	0.63	11	11
GMPR+DESeq2	0.89	0.88	0.88	0.82	0.82	0.82	4	17
corncob	0.60	0.67	0.73	0.28	0.36	0.45	16	3
GMPR+edgeR	0.83	0.85	0.86	0.74	0.75	0.75	4	15
DACOMP	0.63	0.71	0.77	0.12	0.36	0.50	13	4.5
ANCOM-BC	0.85	0.85	0.88	0.70	0.71	0.71	4	13.5
eBay(Wilcox)	0.86	0.87	0.88	0.68	0.69	0.70	4	13.5
Aldex2(Wilcox)	0.71	0.75	0.79	0.45	0.48	0.55	9	7
GMPR+glm	0.30	0.32	0.34	0.21	0.24	0.24	14	1
Wrench+MSeq	0.64	0.70	0.72	0.39	0.42	0.47	9	6
Omnibus	0.74	0.74	0.75	0.60	0.62	0.65	4	10
TSS+Wilcox	0.74	0.77	0.80	0.47	0.51	0.56	4	8
RAIDA	0.60	0.63	0.69	0.31	0.34	0.39	9	2
GMPR+Wilcox	0.50	0.49	0.51	0.49	0.49	0.50	4	4.5

Fig. S21

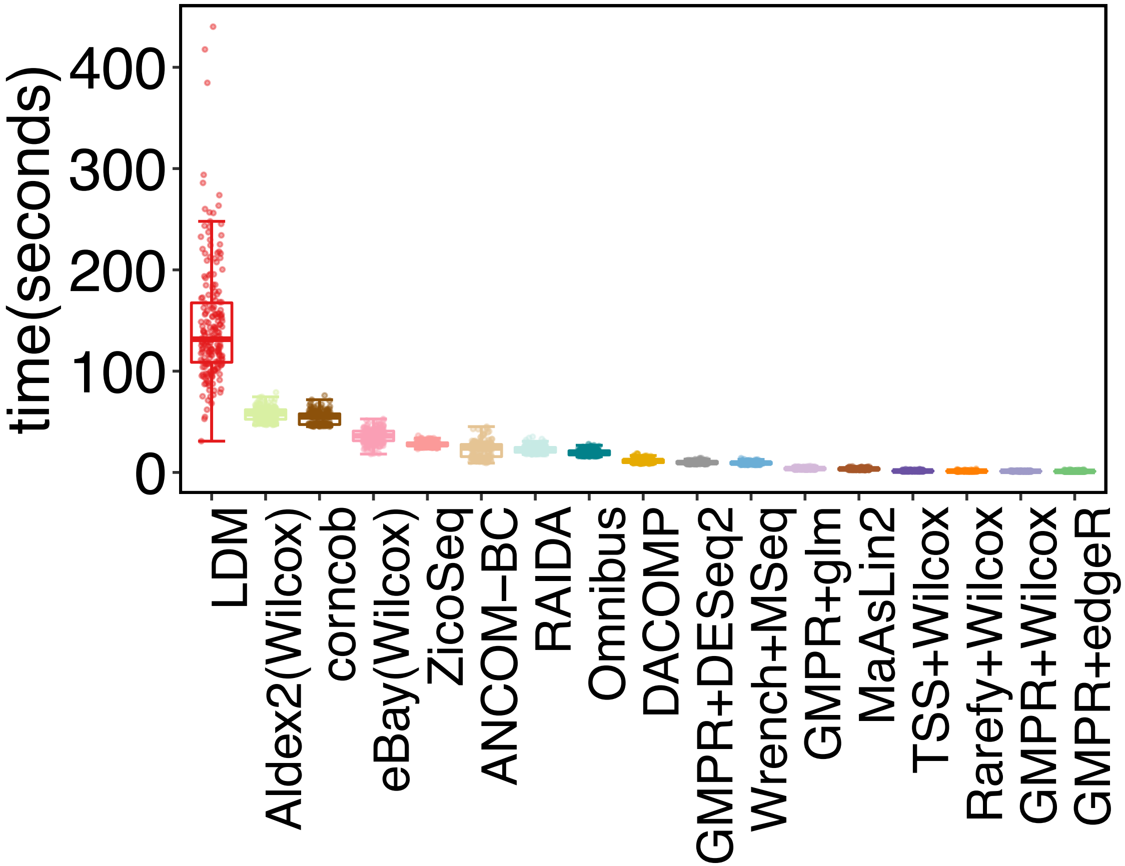
time(seconds)
400
300
200
100
0
LDM
Aldex2(Wilcox)
corncob
eBay(Wilcox)
ZicoSeq
ANCOM−BC
RAIDA
Omnibus
DACOMP
GMPR+DESeq2
Wrench+MSeq
GMPR+glm
MaAsLin2
TSS+Wilcox
Rarefy+Wilcox
GMPR+Wilcox
GMPR+edgeR

Fig.S22

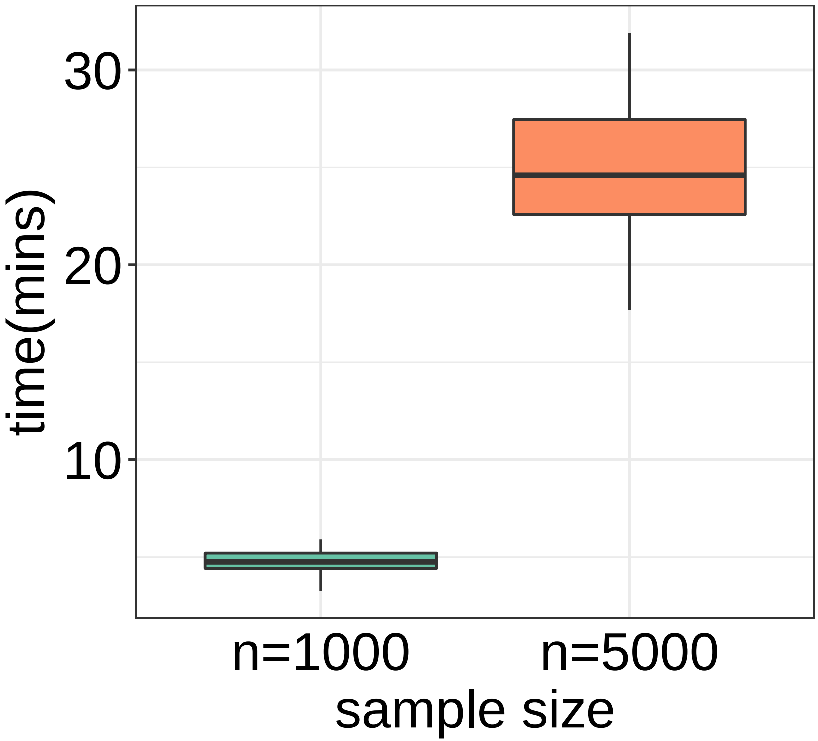

Fig. S23

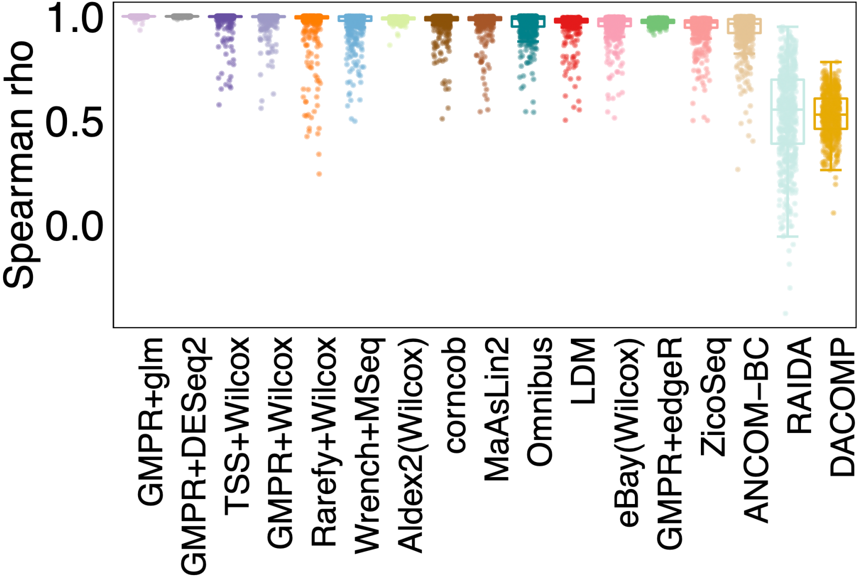
Spearman rho
1.0
0.5
0.0
GMPR+glm
GMPR+DESeq2
TSS+Wilcox
GMPR+Wilcox
Rarefy+Wilcox
Wrench+MSeq
Aldex2(Wilcox)
corncob
MaAsLin2
Omnibus
LDM
eBay(Wilcox)
GMPR+edgeR
ZicoSeq
ANCOM−BC
RAIDA
DACOMP

Fig. S24

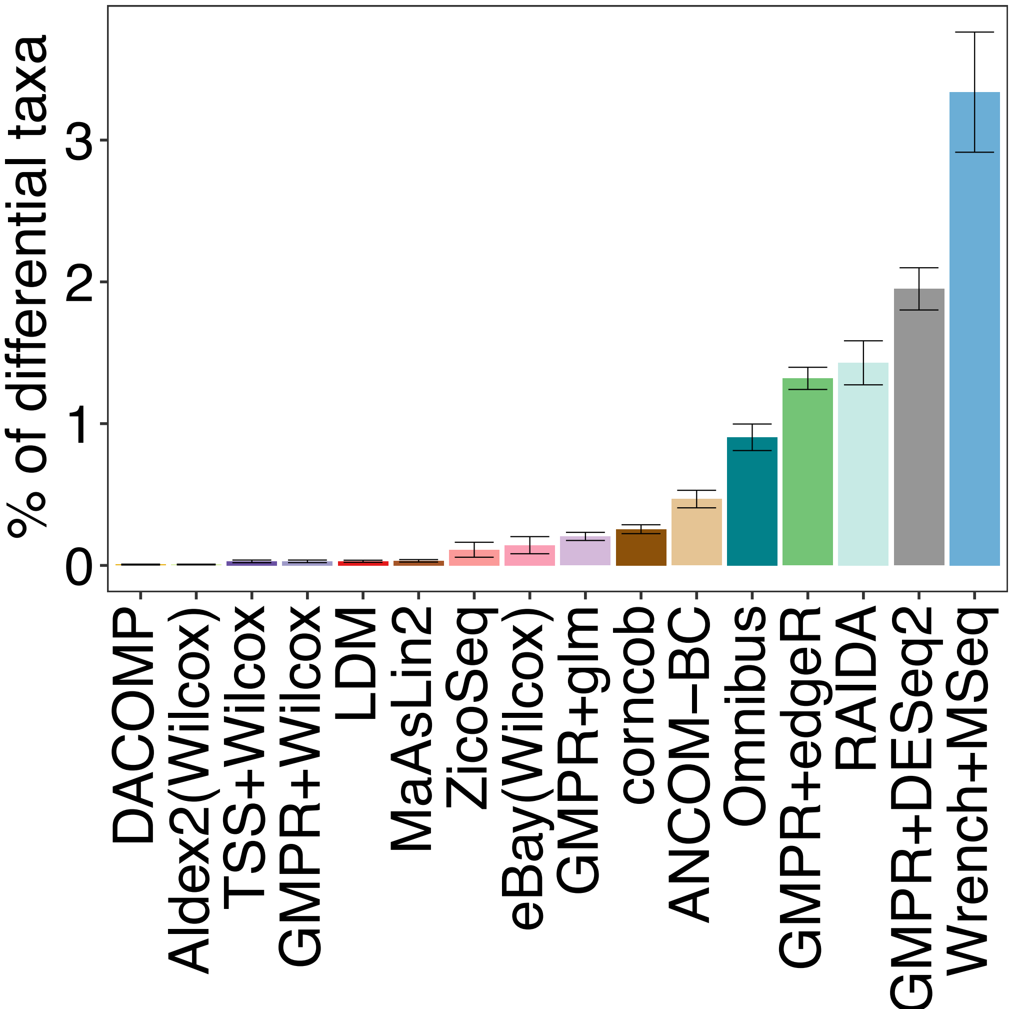
% of differential taxa
0
1
2
3
DACOMP
Aldex2(Wilcox)
TSS+Wilcox
GMPR+Wilcox
LDM
MaAsLin2
ZicoSeq
eBay(Wilcox)
GMPR+glm
corncob
ANCOM−BC
Omnibus
GMPR+edgeR
RAIDA
GMPR+DESeq2
Wrench+MSeq

Fig. S25

a

Signal density	Abundant			Rare			Rank FDR	Rank TPR
pct60	0.90	0.91	0.88	0.78	0.69	0.76	3.5	2
pct20	0.94	0.97	0.93	0.89	0.86	0.89	1	4
pct40	0.92	0.94	0.91	0.83	0.75	0.83	2	3
pct80	0.86	0.86	0.86	0.72	0.54	0.67	3.5	1

b

Signal density	Abundant			Rare			Rank FDR	Rank TPR
pct40	0.79	0.67	0.76	0.49	0.37	0.49	3	3
pct20	0.86	0.73	0.83	0.64	0.51	0.64	1	4
pct60	0.67	0.49	0.62	0.37	0.28	0.35	3	2
pct80	0.48	0.28	0.41	0.19	0.12	0.15	3	1

c

	Ensemble			Rank	
Signal density	High denisty	Low denisty	Medium denisty	FDR	TPR
pct20	0.85	0.83	0.85	1	4
pct40	0.74	0.77	0.77	2	3
pct60	0.61	0.68	0.68	3	2
pct80	0.49	0.59	0.59	4	1

d

	Ensemble			Rank	
Signal density	High denisty	Low denisty	Medium denisty	FDR	TPR
pct20	0.65	0.65	0.67	1	4
pct40	0.34	0.48	0.50	2	3
pct60	0.11	0.20	0.23	3	2
pct80	0.03	0.09	0.09	4	1

a

Signal density	Abundant Low	Abundant Medium	Abundant High	Rare Low	Rare Medium	Rare High	Rank FDR	Rank TPR
ZicoSeq	0.83	0.85	0.88	0.64	0.71	0.76	3	4
fastANCOM	0.77	0.80	0.83	0.49	0.58	0.66	3	2
ZINQ	0.54	0.60	0.68	0.17	0.24	0.35	3	1
LinDA	0.78	0.82	0.84	0.58	0.65	0.71	1	3

b

Signal density	Abundant Low	Abundant Medium	Abundant High	Rare Low	Rare Medium	Rare High	Rank FDR	Rank TPR
ZicoSeq	0.63	0.70	0.77	0.40	0.53	0.63	3.5	4
fastANCOM	0.40	0.46	0.54	0.13	0.18	0.23	3.5	2
LinDA	0.49	0.56	0.64	0.35	0.45	0.54	1	3
ZINQ	0.29	0.35	0.41	0.04	0.05	0.07	2	1

c

Signal density	Stool Low	Stool Medium	Stool High	Rank FDR	Rank TPR
ZicoSeq	0.81	0.84	0.85	4	4
fastANCOM	0.75	0.76	0.74	3	2
LinDA	0.79	0.81	0.85	1.5	3
ZINQ	0.45	0.39	0.26	1.5	1

d

Signal density	Vaginal Low	Vaginal Medium	Vaginal High	Rank FDR	Rank TPR
ZicoSeq	0.61	0.64	0.69	3.5	4
fastANCOM	0.29	0.30	0.15	3.5	2
LinDA	0.41	0.43	0.32	2	3
ZINQ	0.25	0.24	0.08	1	1

Supplementary Table 1 Normalization methods for microbiome data.

Methods	Description
Total sum scaling (TSS)	The TSS size factor is simply the total number of reads in the sample.
Trimmed mean of M values (TMM)	The TMM method first selects a reference sample, then all other samples are compared to the reference sample. The weighted trimmed mean of log-ratios between each pair of samples is then calculated as the TMM size factor.
Relative Log Expression (RLE)	The RLE method calculates the geometric means of all features as a "reference," and all samples are compared to the "reference" to produce ratios for all features. The median ratio is then taken to be the RLE size factor.
Cumulative sum scaling (CSS)	The CSS size factor is the cumulative sum of counts up to a percentile determined by a data-driven approach.
Centered log-ratio transformation (CLR)	For each sample, the counts are divided by their geometric mean, followed by log transformation. Thus, the CLR size factor is the geometric mean of the counts in a sample.
Geometric mean of pairwise ratios (GMPR)	For each sample, the GMPR method calculates the pairwise ratios to all other samples for each feature. The size factor is then the geometric mean of the median ratios for all features.
Wrench	Wrench models feature-wise proportion ratios to a reference sample using a hurdle log-normal model, where a compositional scale factor is included so that the log fold changes on the absolute abundance level is centered at 0 (the majority of the taxa do not change).

Supplementary Table 2 Package version and source link for the differential abundance analysis methods evaluated in this study

Method	Package version	Source link
GMPR+Wilcox<br>TSS+Wilcox<br>Rarefy+Wilcox	stats_3.6.2	https://www.rdocumentation.org/packages/stats/versions/3.6.2
DESeq2	DESeq2_1.26.0	http://bioconductor.org/packages/release/bioc/html/DESeq2.html
GMPR+DESeq2		https://github.com/jchen1981/GMPR
edgeR	edgeR_3.28.1	http://bioconductor.org/packages/release/bioc/html/edgeR.html
GMPR+edgeR		https://github.com/jchen1981/GMPR
Wrench+MSeq	metagenomeSeq_1.28.2	https://www.bioconductor.org/packages/release/bioc/html/metagenomeSeq.html
RAIDA	RAIDA_1.0	https://cals.arizona.edu/~anling/sbg/software.htm
ANCOM-BC	ANCOMBC_0.99.5	https://github.com/FrederickHuangLin/ANCOMBC
DACOMP	dacomp_1.23	https://github.com/barakbri/dacomp
LDM	LDM_1.0	https://github.com/yijuanhu/LDM
Omnibus	mbzinb_0.2	https://github.com/jchen1981/MicrobiomeDDA
Aldex2	ALDEx2_1.18.0	https://github.com/ggloor/ALDEx_bioc
GMPR+glm	stats_3.6.2	https://www.rdocumentation.org/packages/stats/versions/3.6.2
corncob	corncob_0.1.0	https://github.com/bryandmartin/corncob
MaAsLin2	Maaslin2_1.4.0	https://github.com/biobakery/biobakery/wiki/maaslin2
eBay	eBay_0.1	https://github.com/liudoubletian/eBay

Supplementary Table 3 Performance scoring system

Fasle positive control			Power scoring system	
FDR	Rating	FDR score	TPR rank (16 methods in ranking)	TPR score
[0,0.05)[#]	***	3	highest TPR among 16 methods	16
<= 0.1	**	2	2nd higest TPR among 16 methods	15
(0.1,0.2]	*	1	……	…
(0.2,1]	x	0	lowest TPR score among 16 methods	1

[#]Rating "***" is given when the 95% confidence interval of the FDR estimate covers 0.05.

Supplementary Table 4 The main evaluation criteria applied in the summary heatmap (Fig. 7)

	Good	Intermediate	Poor
FDR (observed false discovery rate, aveaged over all simulation runs)	(0,0.05]	(0.05,0.1]	(0.1,1]
TPR (true positive rate, averaged over all simulation runs)	> 10	(5,10]	(0,5]
Speed (mins)	< 1	[1-10]	> 10
Complex design (support continuous variable, covariate adjustment?)	continuous&covariate	continuous	neither
Stability (Spearman's rho: 0% v.s. 40% prevelance filter)	> 0.9	[0.7,0.9]	< 0.7
FDR (global null)	(0,0.05]	(0.05,0.1]	> 0.1

Supplementary Table 5 Details of the experimental datasets

Dataset name	Taxa number	Sample size	2-group	Body site	Links
ShiB_2015	154	48	periodontitis;SRP	oralcavity	
Castro_NallarE_2015	128	32	schizophrenia;control	oralcavity	
BritoIL_2016	218	1018	Fiji;USA	oralcavity;stool	
ChngKR_2016	105	78	AD;control	skin	
TettAJ_2016	52	83	control;psoriasis	skin	
JieZ_2017	192	385	ACVD;control	stool	
LoombaR_2017	145	86	advanced;moderate	stool	
ChengpingW_2017	131	211	AS;control	stool	
DhakanDB_2019	91	110	Bhopal;Kasaragod	stool	
LiJ_2014	148	249	DNK;ESP	stool	
TettAJ_2019_ab	113	112	TZA;GHA	stool	
TettAJ_2019_bc	113	112	GHA;ETH	stool	
TettAJ_2019_ac	118	94	ETH;TZA	stool	
Obregon_TitoAJ_2015	124	58	PER;USA	stool	
LiuW_2016	140	257	MNG;USA	stool	
PehrssonE_2016	105	191	PER;SLV	stool	
RampelliS_2015	89	38	TZA;ITA	stool	
KieserS_2018	145	207	control;acute_diarrhoea	stool	
FengQ_2015_1	159	108	control;adenoma	stool	
HanniganGD_2017_2	82	54	control;adenoma	stool	
ThomasAM_2018a_1	106	51	control;adenoma	stool	
YeZ_2018	103	65	control;BD	stool	
VatanenT_2016	107	785	newborn;child	stool	
VincentC_2016	176	229	control;CDI	stool	
RaymondF_2016	115	72	control;cephalosporins	stool	https://bioconductor.org/packages/release/data/experiment/html/curatedMetagenomicData.html
QinN_2014	166	237	control;cirrhosis	stool	
ZellerG_2014_1	168	108	control;CRC;adenoma	stool	
NielsenHB_2014	159	396	control;IBD	stool	
KosticAD_2015	104	120	control;T1D	stool	
LiSS_2016	108	45	FMT;control	stool	
LiJ_2017	117	140	hypertension;control	stool	
KarlssonFH_2013_1	141	92	IGT;control	stool	
DavidLA_2015	96	47	infectiousgastroenteritis;control	stool	
HansenLBS_2018	145	207	Low-gluten diet;Low-gluten diet	stool	
LiSS_2016_1	96	15	metabolic_syndrome;control	stool	
LeChatelierE_2013	134	292	obese;non-obese	stool	
LiJ_2017_1	108	97	pre-hypertension;control	stool	
Heitz_BuschartA_2016	121	53	T1D;control	stool	
FerrettiP_2018	112	215	newborn;adult	stool;oralcavity;vagina;skin	
FengQ_2015	166	107	CRC;healthy control	stool	
HanniganGD_2017	84	55	CRC;healthy control	stool	
KarlssonFH_2013	138	96	T2D;healthy control	stool	
QinJ_2012	155	344	T2D;healthy control	stool	
ThomasAM_2018a	120	53	CRC;healthy control	stool	
ThomasAM_2018b	144	60	CRC;healthy control	stool	
VogtmannE_2016	155	104	CRC;healthy control	stool	
YuJ_2015	179	128	CRC;healthy control	stool	
ZellerG_2014	182	157	CRC;healthy control	stool	
Airways_Skin	801	797	Airways;Skin	Airways;Skin	
Airways_Urogenital_Tract	541	313	Airways;Urogenital Tract	Airways;Urogenital Tract	
Attached_Keratinized_Gingiva_Palatine_Tonsils	1699	364	Attached Keratinized Gingiva;Palatine Tonsils	Attached Keratinized Gingiva;Palatine Tonsils	
Attached_Keratinized_Gingiva_Subgingival_Plaque	1804	363	Attached Keratinized Gingiva;Subgingival Plaque	Attached Keratinized Gingiva;Subgingival Plaque	
Attached_Keratinized_Gingiva_Supragingival_Plaque	1806	368	Attached Keratinized Gingiva;Supragingival Plaque	Attached Keratinized Gingiva;Supragingival Plaque	
Attached_Keratinized_Gingiva_Throat	1661	350	Attached Keratinized Gingiva;Throat	Attached Keratinized Gingiva;Throat	
Buccal_Mucosa_Attached_Keratinized_Gingiva	1559	361	Buccal Mucosa;Attached Keratinized Gingiva	Buccal Mucosa;Attached Keratinized Gingiva	
Buccal_Mucosa_Palatine_Tonsils	1946	365	Buccal Mucosa;Palatine Tonsils	Buccal Mucosa;Palatine Tonsils	
Buccal_Mucosa_Subgingival_Plaque	2087	364	Buccal Mucosa;Subgingival Plaque	Buccal Mucosa;Subgingival Plaque	
Buccal_Mucosa_Supragingival_Plaque	1374	277	Buccal Mucosa;Supragingival Plaque	Buccal Mucosa;Supragingival Plaque	
Buccal_Mucosa_Throat	1925	351	Buccal Mucosa;Throat	Buccal Mucosa;Throat	
Gastrointestinal_Tract_Airways	865	258	Gastrointestinal Tract;Airways	Gastrointestinal Tract;Airways	
Gastrointestinal_Tract_Oral	1192	1346	Gastrointestinal Tract;Oral	Gastrointestinal Tract;Oral	
Gastrointestinal_Tract_Skin	489	619	Gastrointestinal Tract;Skin	Gastrointestinal Tract;Skin	
Gastrointestinal_Tract_Urogenital_Tract	1115	446	Gastrointestinal Tract;Urogenital Tract	Gastrointestinal Tract;Urogenital Tract	
Hard_Palate_Attached_Keratinized_Gingiva	1628	356	Hard Palate;Attached Keratinized Gingiva	Hard Palate;Attached Keratinized Gingiva	
Hard_Palate_Buccal_Mucosa	1855	357	Hard Palate;Buccal Mucosa	Hard Palate;Buccal Mucosa	
Hard_Palate_Palatine_Tonsils	1873	360	Hard Palate;Palatine Tonsils	Hard Palate;Palatine Tonsils	
Hard_Palate_Subgingival_Plaque	2171	359	Hard Palate;Subgingival Plaque	Hard Palate;Subgingival Plaque	
Hard_Palate_Supragingival_Plaque	2140	364	Hard Palate;Supragingival Plaque	Hard Palate;Supragingival Plaque	
Hard_Palate_Throat	1888	346	Hard Palate;Throat	Hard Palate;Throat	
Left_Retroauricular_Crease_Left_Antecubital_Fossa	416	241	Left Retroauricular Crease;Left Antecubital Fossa	Left Retroauricular Crease;Left Antecubital Fossa	
Left_Retroauricular_Crease_Right_Antecubital_Fossa	446	237	Left Retroauricular Crease;Right Antecubital Fossa	Left Retroauricular Crease;Right Antecubital Fossa	
Left_Retroauricular_Crease_Right_Retroauricular_Crease	748	368	Left Retroauricular Crease;Right Retroauricular Crease	Left Retroauricular Crease;Right Retroauricular Crease	
Mid_Vagina_Posterior_Fornix	594	174	Mid Vagina;Posterior Fornix	Mid Vagina;Posterior Fornix	
Oral_Airways	1221	1327	Oral;Airways	Oral;Airways	
Oral_Skin	974	1688	Oral;Skin	Oral;Skin	https://bioconductor.org/packages/release/data/experiment/html/HMP16SData.html
Oral_Urogenital_Tract	1106	1390	Oral;Urogenital Tract	Oral;Urogenital Tract	
Palatine_Tonsils_Subgingival_Plaque	2281	367	Palatine Tonsils;Subgingival Plaque	Palatine Tonsils;Subgingival Plaque	
Palatine_Tonsils_Supragingival_Plaque	2164	372	Palatine Tonsils;Supragingival Plaque	Palatine Tonsils;Supragingival Plaque	
Palatine_Tonsils_Throat	1886	354	Palatine Tonsils;Throat	Palatine Tonsils;Throat	
Right_Retroauricular_Crease_Left_Antecubital_Fossa	400	242	Right Retroauricular Crease;Left Antecubital Fossa	Right Retroauricular Crease;Left Antecubital Fossa	
Right_Retroauricular_Crease_Right_Antecubital_Fossa	442	238	Right Retroauricular Crease;Right Antecubital Fossa	Right Retroauricular Crease;Right Antecubital Fossa	
Saliva_Attached_Keratinized_Gingiva	1759	339	Saliva;Attached Keratinized Gingiva	Saliva;Attached Keratinized Gingiva	
Saliva_Buccal_Mucosa	1989	340	Saliva;Buccal Mucosa	Saliva;Buccal Mucosa	
Saliva_Hard_Palate	1938	335	Saliva;Hard Palate	Saliva;Hard Palate	
Saliva_Palatine_Tonsils	2021	343	Saliva;Palatine Tonsils	Saliva;Palatine Tonsils	
Saliva_Subgingival_Plaque	2231	342	Saliva;Subgingival Plaque	Saliva;Subgingival Plaque	
Saliva_Supragingival_Plaque	1404	260	Saliva;Supragingival Plaque	Saliva;Supragingival Plaque	
Saliva_Throat	1976	329	Saliva;Throat	Saliva;Throat	
Saliva_Tongue_Dorsum	1250	262	Saliva;Tongue Dorsum	Saliva;Tongue Dorsum	
Skin_Urogenital_Tract	395	674	Skin;Urogenital Tract	Skin;Urogenital Tract	
Supragingival_Plaque_Subgingival_Plaque	2115	371	Supragingival Plaque;Subgingival Plaque	Supragingival Plaque;Subgingival Plaque	
Throat_Subgingival_Plaque	2165	353	Throat;Subgingival Plaque	Throat;Subgingival Plaque	
Throat_Supragingival_Plaque	2163	358	Throat;Supragingival Plaque	Throat;Supragingival Plaque	
Tongue_Dorsum_Attached_Keratinized_Gingiva	1716	370	Tongue Dorsum;Attached Keratinized Gingiva	Tongue Dorsum;Attached Keratinized Gingiva	
Tongue_Dorsum_Buccal_Mucosa	1409	278	Tongue Dorsum;Buccal Mucosa	Tongue Dorsum;Buccal Mucosa	
Tongue_Dorsum_Hard_Palate	1862	366	Tongue Dorsum;Hard Palate	Tongue Dorsum;Hard Palate	
Tongue_Dorsum_Palatine_Tonsils	1865	374	Tongue Dorsum;Palatine Tonsils	Tongue Dorsum;Palatine Tonsils	
Tongue_Dorsum_Subgingival_Plaque	2199	373	Tongue Dorsum;Subgingival Plaque	Tongue Dorsum;Subgingival Plaque	
Tongue_Dorsum_Supragingival_Plaque	2198	378	Tongue Dorsum;Supragingival Plaque	Tongue Dorsum;Supragingival Plaque	
Tongue_Dorsum_Throat	1852	360	Tongue Dorsum;Throat	Tongue Dorsum;Throat	
Vaginal_Introitus_Mid_Vagina	657	174	Vaginal Introitus;Mid Vagina	Vaginal Introitus;Mid Vagina	
Vaginal_Introitus_Posterior_Fornix	622	172	Vaginal Introitus;Posterior Fornix	Vaginal Introitus;Posterior Fornix	
USA_Malawi	815	430	USA;Malawi	stool	
USA_Venezuela	846	415	USA;Venezuela	stool	https://github.com/FrederickHuangLin/ANCOM-BC-Code-Archive/tree/master/data/global_gut
Malawi_Venezuela	662	213	Malawi;Venezuela	stool	
Morton_2019	163	32	before;after	oral	https://github.com/knightlab-analyses/reference-frames/tree/master/data
